# A direct role for a mitochondrial targeting sequence in signaling stress

**DOI:** 10.1038/s41586-025-09834-x

**Published:** 2025-12-10

**Authors:** Zixuan Yuan, Megan Balzarini, Marina Volpe, Madeleine Goldstein, Tony Shengzhe Peng, Elizabeth Hui, Nancy Neng Fang, Waleed S Albihlal, Melika Hajimohammadi, Kevin Wei, Calvin K. Yip, Folkert J van Werven, Thibault Mayor, Hilla Weidberg

**Affiliations:** 1 Life Sciences Institute, Department of Cellular and Physiological Sciences, University of British Columbia, Vancouver, BC, Canada; 2 Department of Biochemistry and Molecular Biology, Michael Smith Laboratories, University of British Columbia, Vancouver, BC, Canada; 3 The Francis Crick Institute, Cell Fate and Gene Regulation Laboratory, London, UK; 4 Life Sciences Institute, Department of Zoology, University of British Columbia, Vancouver, BC, Canada; 5 Life Sciences Institute, Department of Biochemistry and Molecular Biology, University of British Columbia, Vancouver, BC, Canada; 6 Edwin SH Leong Centre for Healthy Aging, University of British Columbia, Vancouver, BC, Canada

## Abstract

Mitochondrial protein import is required for maintaining organellar function^1^. Perturbations in this process are associated with various physiological and disease conditions^2^. Several stress responses, including the mitoCPR, combat damage caused by mitochondrial protein import defects^2^. However, how this defect is sensed remains largely unknown. Here, we reveal that the conserved mitochondrial Hsp70 co-chaperone, Mge1, acts as a stress messenger in budding yeast. During mitochondrial stress, unimported Mge1 entered the nucleus and triggered the transcription of mitoCPR target genes. This was mediated by Mge1's interaction with the transcription factor Pdr3 on DNA regulatory elements. Mge1's mitochondrial targeting sequence was both sufficient and essential for mitoCPR induction, demonstrating that in addition to their roles in mitochondrial protein import, targeting sequences can also function as signaling molecules. As protein import defects are a common consequence of various types of mitochondrial damage^3,4^, these findings suggest a novel function for Mge1's targeting sequence as an indicator of mitochondrial health.

Mitochondria are essential organelles and hubs for cellular metabolism and signaling ^5^. Their proteome includes over 1,000 proteins, most of which are synthesized on cytosolic ribosomes and post-translationally translocated into the organelle^1^. The majority of these precursor proteins contain a 15-55-residue N-terminal mitochondrial targeting sequence (MTS, or presequence)^1,6,7^. These presequences mediate protein translocation through the mitochondrial outer and inner membrane translocases, TOM and TIM, and are cleaved upon entry into the matrix^1^. Complete import of precursors requires an ATP-dependent Hsp70 motor composed of TIM subunits, mitochondrial Hsp70, and its cochaperones^1,8^.

Inefficient protein import not only depletes mitochondria of essential factors, but also leads to the accumulation of precursors in other parts of the cell, compromising both mitochondrial and cellular homeostasis^1,2,9,10^. As such, protein import defects were shown to impact various diseases, including neurodegenerative and bioenergetic disorders^2,5,10^. Cells counteract protein import stress through transcriptional programs that limit cytosolic translation and enhance protein quality control, or through mitochondria-specific pathways such as the unfolded protein response (UPR^mt^)^2–4,9,11–22^. While the mechanisms of UPR^mt^ activation are well characterized, how other mitochondrial stresses are communicated to the nucleus remains unclear.

It was recently demonstrated that impaired mitochondrial protein import can lead to the stalling of precursor proteins within TOM^11,21,23–27^. This defect can be repaired by the ATPases Cdc48/p97 and Msp1/ATAD1, which extract stalled polypeptides and clear the translocase^21,25,27^. In budding yeast, efficient recruitment of Msp1 to clogged translocases requires the stress-induced adaptor, *CIS1*^21^. This gene is a target of the mitochondrial compromised protein import response (mitoCPR), a surveillance pathway controlled by the transcription factor Pdr3^21^. How a mitochondrial stress signal is transmitted to activate Pdr3 remains unknown.

Here, we identify the mitochondrial Hsp70 co-chaperone Mge1/GrpEL as a central regulator of the mitoCPR. When protein import is compromised, the precursor of Mge1 translocates to the nucleus to activate Pdr3. This function is mediated by Mge1's N-terminal presequence. Our work revealed that mitochondrial targeting peptides can act as transmitters of mitochondrial stress in addition to their traditional role in protein targeting and import.

## Overexpressed Mge1 activates the mitoCPR

**Extended Data Fig. 1:**
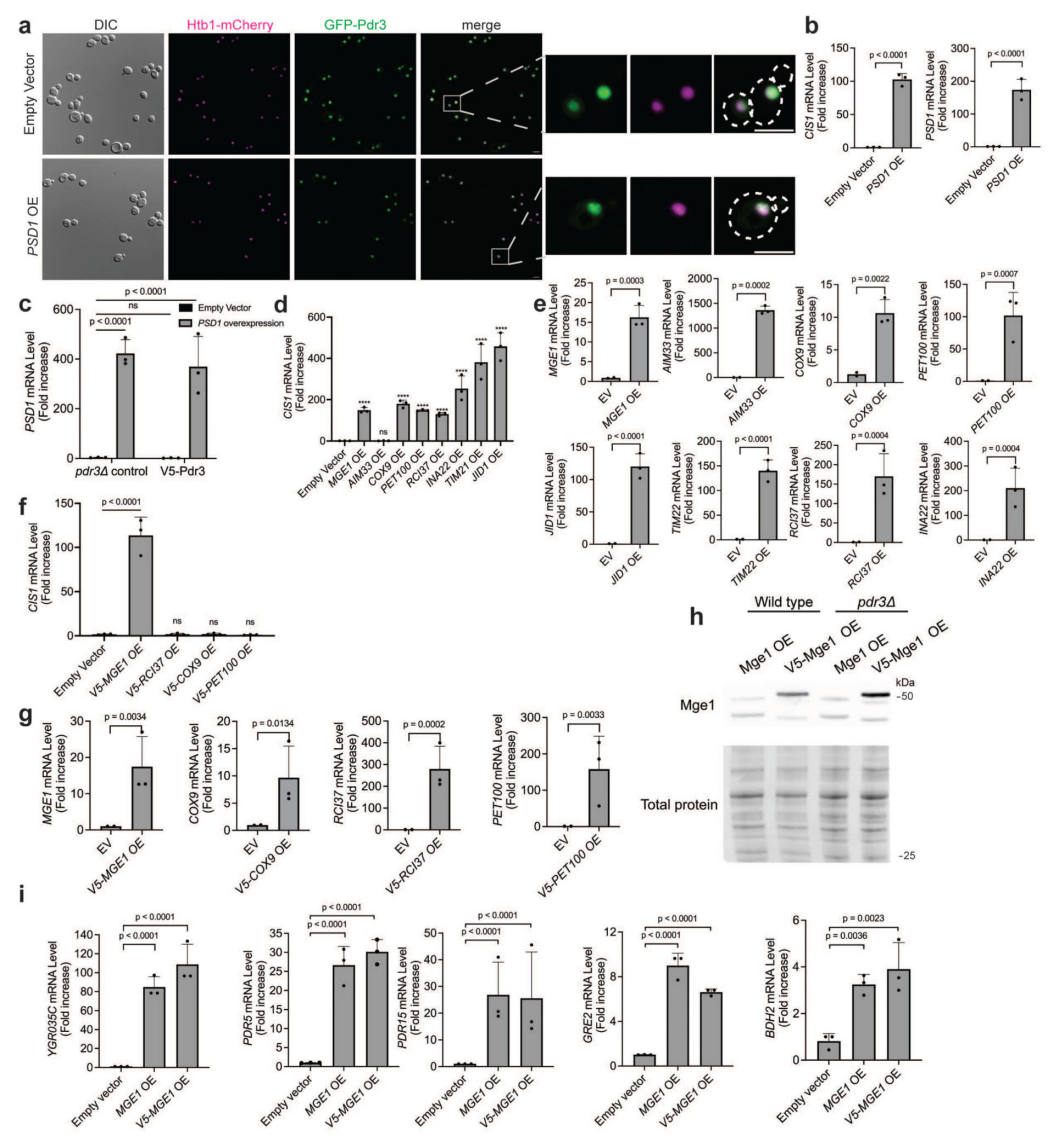
Overexpression of Mge1 leads to upregulation of mitoCPR target genes. **(a)** Live-cell fluorescence imaging of cells expressing *P_TEF2_-GFP-PDR3* and the nuclear marker *HTB1-mCherry* under control (empty vector) and impaired mitochondrial protein import (*PSD1* overexpression) conditions. Zoom-in images of representative cells are shown on the right. Scale bars, 5 μm. OE, overexpression. (**b**) *CIS1* and *PSD1* mRNA level in cells used in (a) and main Figure 1a. n=3 biological replicates; Two-tailed t-test. OE, overexpression. (**c**) *PSD1* mRNA level in cells used for ChIP analysis (Fig. 1b). One-way ANOVA followed by Tukey's test. (**d**) *CIS1* mRNA levels in control cells (empty vector, n=3 biological repeats) and cells overexpressing the indicated mitochondrial genes (n=3 biological repeats). Overexpression was induced by the addition of galactose for 4 hours. One-way ANOVA followed by Dunnett's test; **** P ≤ 0.0001. OE, overexpression. (**e**) mRNA levels of the indicated overexpressed genes (n=3 biological repeats) compared to empty vector control (n=2 biological repeats) following 4-hour galactose induction. Two-tailed t-test. EV, empty vector, OE, overexpression (**f**) Same as (d). The indicated overexpressed mitochondrial genes were tagged with 2XV5 at the N-terminus. (**g**) Same analysis as (e). (**h**) Immunoblot of overexpressed Mge1-HA-ProtA and 2V5-Mge1-HA-ProtA following 4-hour galactose induction in wild-type and *PDR3* deleted cells. OE, overexpression. (**i**) mRNA levels of *YGR035C, PDR5, PDR15, GRE2* and *BDH2* in cells overexpressing *MGE1* and 2V5-*MGE1* for 4 hours. n=3 biological replicates; One-way ANOVA followed by Dunnett's test. OE, overexpression. (**b-g, i**) Data represent mean +/- SD; ns, not significant.

**Fig. 1:**
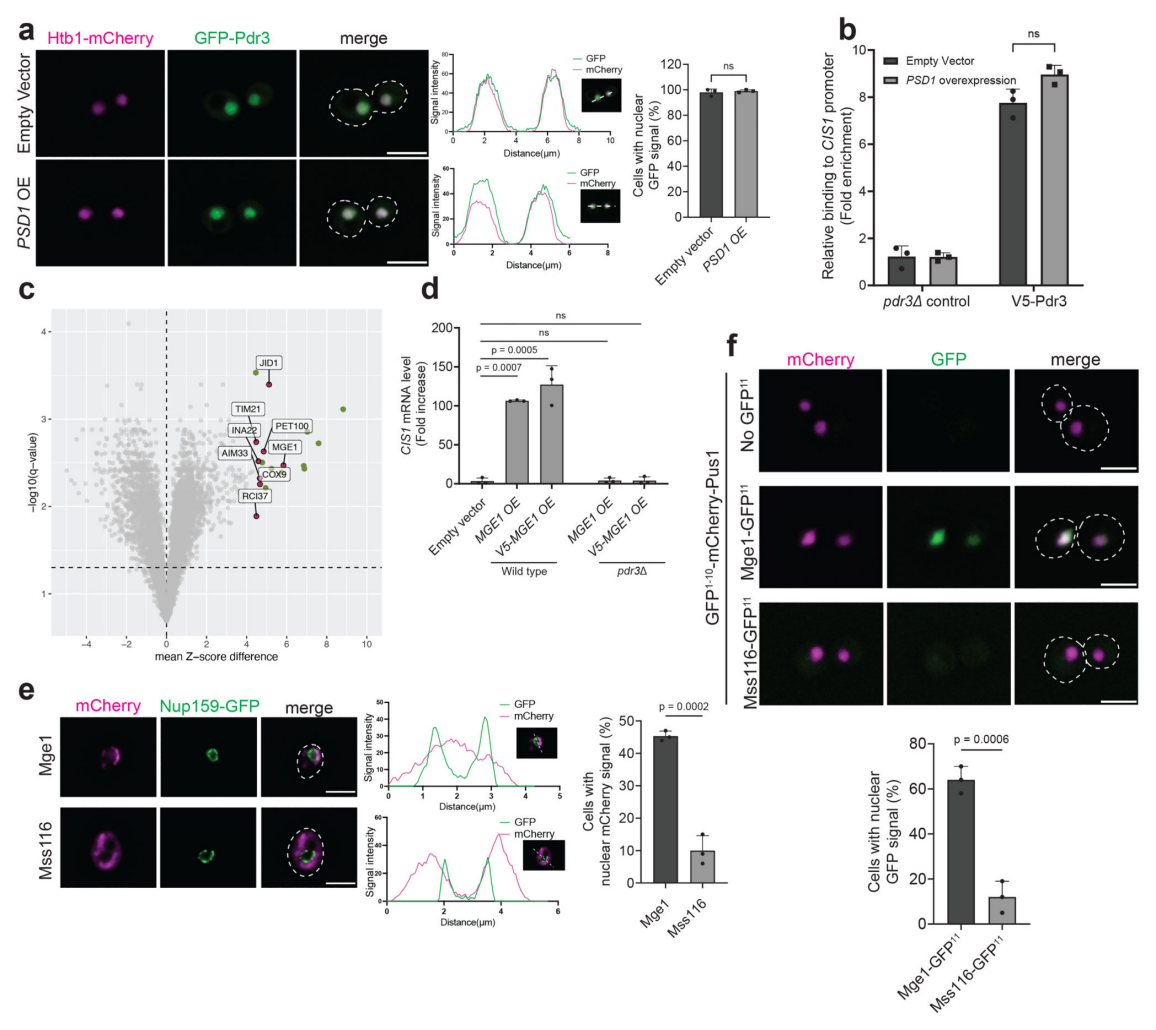
Genetic screen identifies Mge1 as a potential activator of Pdr3. (**a**) Images of cells expressing *P_TEF2_-GFP-PDR3* and the nuclear marker *HTB1-mCherry* under control (empty vector) and impaired mitochondrial protein import (*PSD1^OE^*) conditions. Fluorescence intensity profile along the dashed line is shown. Scale bars, 5 μm. Quantification of cells with nuclear GFP is shown on the right. Images were acquired from three independent experiments (100 cells each); Two-tailed t-test. ns, not significant. OE, overexpression. (**b**) ChIP analysis of *pdr3*Δ cells or cells expressing *P_TEF2_-V5-PDR3* under control (empty vector) and impaired protein import (*PSD1^OE^*) conditions. Pdr3's binding to the promoter of *CIS1* was normalized over *HMR*. n = 3 biological replicates; One-way analysis of Variance (ANOVA) followed by Tukey's test. ns, not significant. (**c**) Volcano plot of the overexpression screen. Positive values of mean Z-score difference indicate increased expression of the mitoCPR reporter. The top 20 ORFs are highlighted: green=non-mitochondrial, magenta=mitochondrial. (**d**) *CIS1* mRNA levels were assessed in control cells (empty vector), or cells overexpressing either *MGE1* or *2V5-MGE1* for 4 hours in the presence or absence of *PDR3*. n=3 biological replicates; One-way ANOVA followed by Dunnett's test. ns, not significant; OE, overexpression. (**e**) Images of cells expressing the nuclear envelope marker Nup159-GFP and either Mge1-mCherry or Mss116-mCherry (Overexpressed from the *GAL1-10* promoter for 4 hours). Fluorescence intensity profile along the dashed line is shown. Scale bars, 5 μm. Quantification of cells with nuclear mCherry from three independent experiments (30 cells each; Two-tailed t-test) is shown on the right. (**f**) Images of cells expressing the nuclear protein GFP^1-10^-mCherry-Pus1 alone or with either Mge1-GFP^11^ or Mss116-GFP^11^ (Overexpressed from the *GAL1-10* promoter for 4 hours). Scale bars, 5 μm. Quantification are from three independent experiments (50 cells each; Two-tailed t-test). (**a,b,d,e,f**) Data represent mean +/- SD.

We previously demonstrated that defects in mitochondrial protein import trigger the mitoCPR via activation of the transcription factor Pdr3^21^. However, the signal that induces Pdr3-mediated transcription remains unknown. Past work demonstrated that Pdr3 and its paralog, Pdr1, constitutively reside in the nucleus and bind DNA regulatory motifs of target genes^28,29^. Indeed, GFP-Pdr3 localized to the nucleus under basal conditions (Fig. 1a and Extended Data Fig. 1a). Overexpression of Psd1, which impairs mitochondrial protein import and activates the mitoCPR, did not affect the nuclear localization of Pdr3 (Fig. 1a and Extended Data Fig. 1a-b)^21^. Moreover, chromatin immunoprecipitation (ChIP) demonstrated that Pdr3 was bound to the promoter of the mitoCPR target gene *CIS1* in both control and *PSD1*-overexpressing cells (Fig. 1b and Extended Data Fig. 1c). These results suggest that Pdr3 does not directly sense mitochondrial defects but instead responds to a signal transmitted to the nucleus when protein import is impaired.

How does Pdr3 sense damage that originates in a different cellular compartment? To address this, we performed a genome-wide overexpression screen for genes that activate the mitoCPR. We used a reporter in which the *LEU2* gene was placed under the promoter of the mitoCPR target, *CIS1*^21^, allowing cells with induced mitoCPR to grow without leucine. The top hits of the screen were enriched for mitochondrial ORFs (8 out of 20) (Fig. 1c and Supplementary Table 1). QPCR analysis confirmed that overexpression of these mitochondrial ORFs upregulated *CIS1*, except for the false-positive genes *AIM33* (Extended Data Fig. 1d-e).

When protein import is impaired, mitochondrial precursors can accumulate in various cellular compartments, including the nucleus^9,16,18^. Overexpression of mitochondrial proteins could result in a similar effect by exceeding the import capacity, leading to some accumulation of overexpressed precursors outside the mitochondria. We hypothesized that one or more of the mitochondrial hits can signal protein import defects to the nucleus. However, overexpression can also indirectly induce the mitoCPR by perturbing mitochondrial functions, as shown previously for proteins containing a bipartite signal, such as Psd1. *TIM21, JID1*, and *INA22*, which are likely to fall into the bipartite category, were therefore excluded from secondary analyses^21,30,31^. To eliminate additional indirect activators, we inserted N-terminal tandem V5 tags upstream of each candidate's MTS. As previously shown for Psd1, these tags mask the MTS and block translocation, thereby preventing mitochondrial damage^13,21^. Unlike their untagged equivalents, the overexpression of most N-terminally tagged ORFs did not trigger *CIS1* upregulation (Extended Data Fig. 1f-g). This result demonstrated that the presence of these proteins outside the mitochondria is not sufficient for Pdr3 activation. We conclude that the over-abundance of these proteins at mitochondria led to organellar defects, including impaired protein import, and thus indirect activation of the mitoCPR. Importantly, overexpression of V5-Mge1 induced *CIS1* expression to a similar level as untagged Mge1, suggesting a direct role in Pdr3 activation (Fig. 1d and Extended Data Fig. 1f-h). Mge1 is a matrix co-chaperone homologous to bacterial GrpE^32^. This co-chaperone mediates nucleotide release from the mitochondrial Hsp70, and thus contributes to protein translocation into the matrix as well as protein folding^32^. To confirm that Mge1 and V5-Mge1 overexpression induced *CIS1* upregulation via the canonical mitoCPR, we validated that this induction was Pdr3-dependent (Fig. 1d and Extended Data Fig. 1h). Additional Pdr3 target genes, including the ABC transporters *PDR5* and *PDR15* were also induced by Mge1 overexpression (Extended Data Fig. 1i).

**Extended Data Fig. 2:**
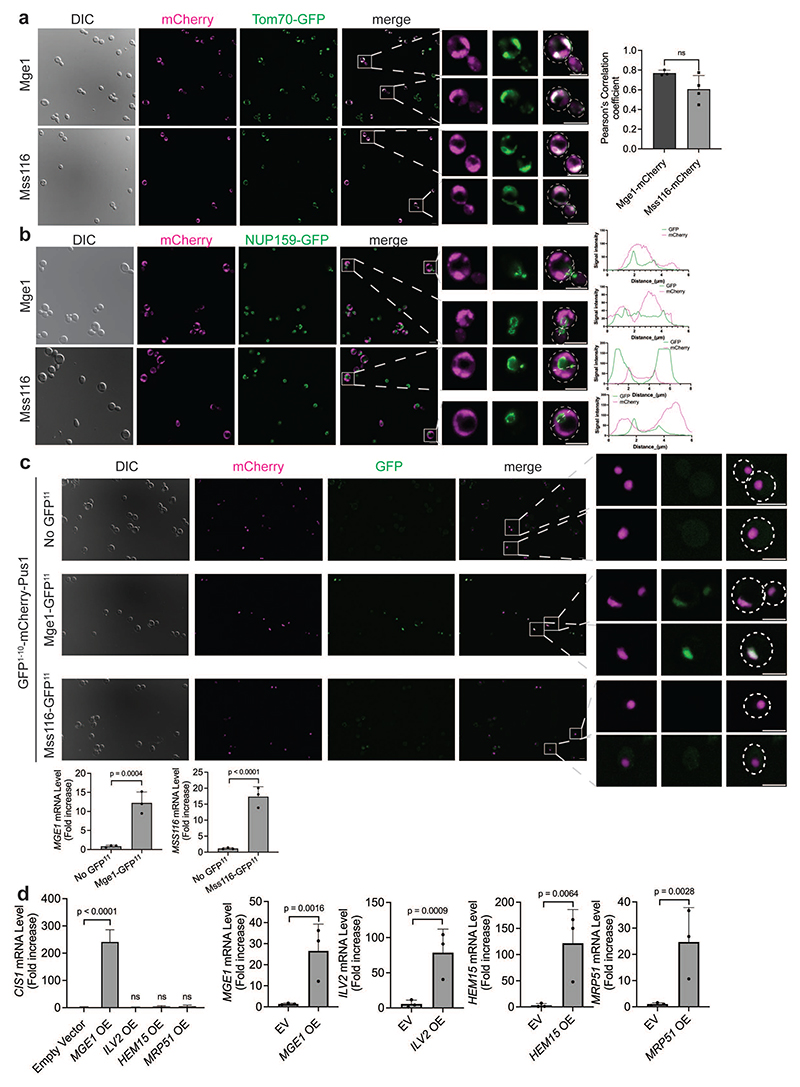
Overexpressed Mge1 localizes to both mitochondria and the nucleus. **(a)** Live-cell fluorescence imaging of cells expressing the mitochondrial marker Tom70-GFP and either Mge1-mCherry (top) or Mss116-mCherry (bottom). Overexpression of *MGE1-mCherry* and *MSS116-mCherry* from the *GAL1-10* promoter was induced by the addition of galactose for 4 hours. Zoom-in images of representative cells are shown on the right. Scale bars, 5 μm. Mean Pearson's correlation coefficients is shown on the right (n=3 independent experiments; in total of 60 cells per strain; Two-tailed t-test). (**b**) Large fields of cells analyzed in Figure 1e, overexpressing Mge1-mCherry (top) or Mss116-mCherry (bottom) as well as the nuclear envelope marker Nup159-GFP. Representative zoom-in images and corresponding fluorescence intensity profiles along the white line are shown on the right. Scale bars, 5 μm. (**c**) Large fields of cells analyzed in Figure 1f, expressing the nuclear protein GFP^1-10^-mCherry-Pus1 alone or with either Mge1-GFP^11^ or Mss116-GFP^11^. Zoom-in images of representative cells are shown on the right. Scale bars, 5 μm. Overexpression of *MGE1-GFP^11^* and *MSS116-GFP^11^* was confirmed by measuring their mRNA levels relative to control cells (bottom panels). n=3 biological replicates; two-tailed t-test. (**d**) *CIS1* mRNA levels in cells overexpressing *MGE1, ILV2, HEM15* or *MRP51* from the *GAL1-10* promoter for 4 hours (left panel; one-way ANOVA followed by Dunnett's test). Overexpression was confirmed by qPCR analysis (right panels; two-tailed t-test). n=3 biological replicates. EV, empty vector; OE, overexpression. (**a, c, d**) Data represent mean +/- SD; ns, not significant.

Overexpressed Mge1-mCherry co-localized with mitochondria, however, unlike the control mitochondrial protein, Mss116, Mge1 was also detected inside nuclei (Fig. 1e and Extended Data Fig. 2a-b). To confirm that overexpressed Mge1 can localize to the nucleus, we used a split-GFP system in which GFP^1-10^ was fused to a nuclear protein and GFP^11^ was fused to Mge1 or Mss116. While no GFP signal was detected in cells expressing the nuclear GFP^1-10^ alone or together with Mss116-GFP^11^, its co-expression with Mge1-GFP^11^ resulted a fluorescent signal in approximately 65% of the cells (Fig. 1f and Extended Data Fig. 2c). These data support the notion that not all Mge1 precursors are imported into the mitochondria upon overexpression, and more importantly that unimported Mge1 may translocate to the nucleus.

A recent study identified a group of mitochondrial precursors that localize to the nucleus under protein import stress conditions^18^. We overexpressed representative proteins from this group to test whether they induce the mitoCPR. These proteins did not trigger *CIS1* upregulation (Extended Data Fig. 2d), suggesting that not all nuclear-localized mitochondrial precursors can activate Pdr3 when overexpressed.

## Mge1 enters the nucleus and binds Pdr3

**Extended Data Fig. 3:**
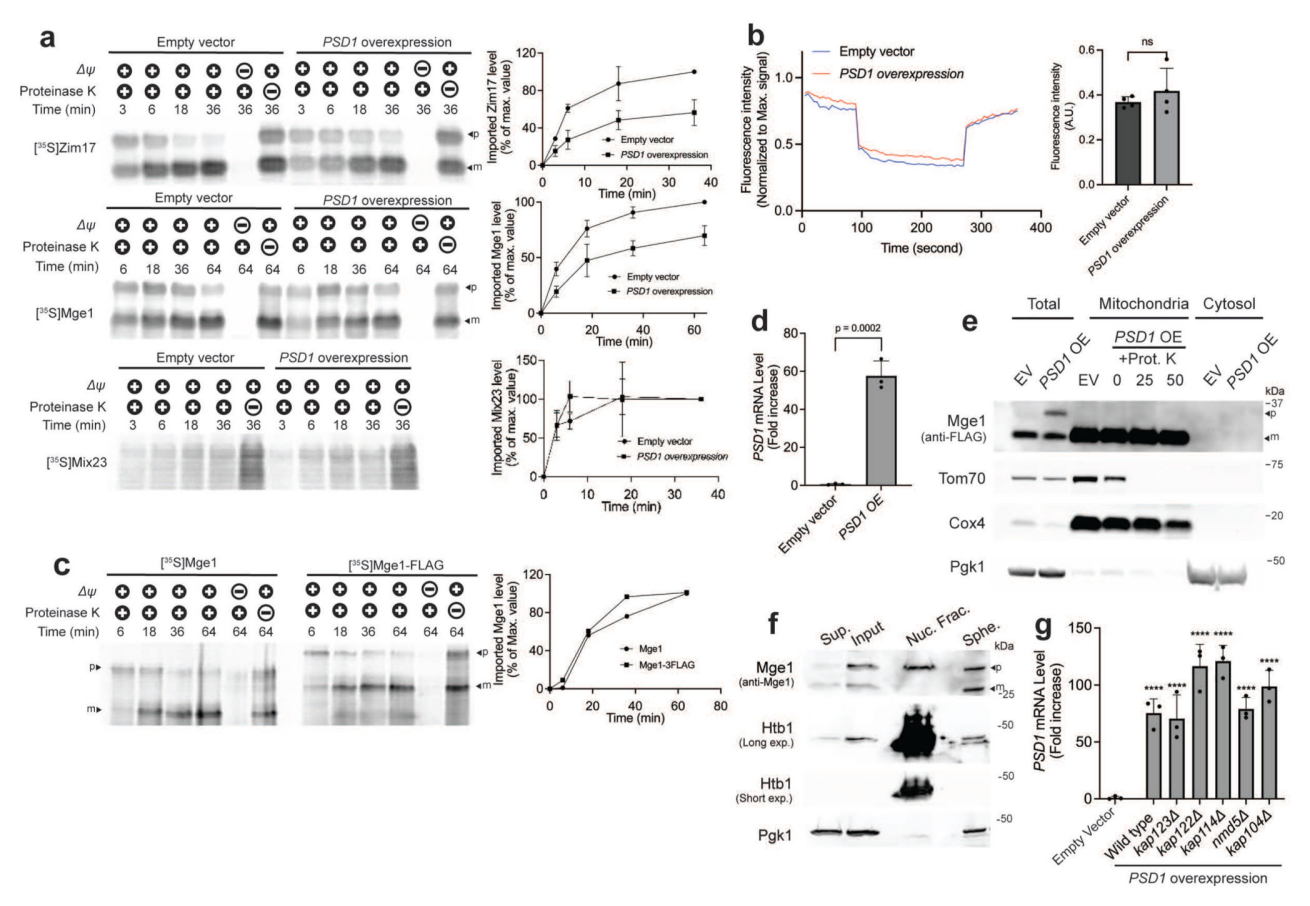
Mge1 precursor accumulates in the nucleus and not inside mitochondria when protein import is impaired. **(a)** *In vitro* protein import assay confirming protein import defects in cells overexpressing *PSD1*. Mitochondria were isolated from control cells (empty vector) or cells overexpressing *PSD1* (4 hours). Mitochondria were incubated with the radiolabeled MTS-containing matrix proteins ^35^S-Zim17 and ^35^S-Mge1 for the indicated durations. Membrane potential (Δψ) was dissipated by preincubation with antimycin A, oligomycin, and valinomycin. Import rate of the intermembrane space protein ^35^S-Mix23, which does not depend on the TIM23 translocase, was used as a control. Quantifications from three biological replicates are shown on the right; protein levels imported into control mitochondria at the final time point was set to 100%. p, precursor; m, mature. **(b)** DiSC3(5) fluorescence measurement of membrane potential in mitochondria isolated from control cells (empty vector) or cells overexpressing *PSD1* (4 hours). Mitochondria and 2 μM of valinomycin were added as indicated. Fluorescent intensity was normalized to the maximal signal for each trace (n = 4 biological replicates; Data are shown as mean). Quantification of mitochondrial membrane potential is shown on the right (Data are shown as mean +/− SD; Two-tailed t-test). ns, not significant. **(c)** *In vitro* protein import assay of radiolabeled ^35^S-Mge1 and ^35^S-Mge1-FLAG into mitochondria isolated from wild-type cells. Membrane potential (Δψ) was dissipated by preincubation with antimycin A, oligomycin, and valinomycin. Quantification of two biological replicates is shown on the right; level of imported ^35^S-Mge1 at the final time point was set to 100%. Data are shown as mean. p, precursor; m, mature. (**d**) Confirmation of *PSD1* overexpression by qPCR analysis for the samples used in Figure 2a. n=3 biological replicates; two-tailed t-test. OE, overexpression. (**e**) Mitochondria were isolated by differential centrifugation from empty vector (EV) control cells or cells overexpressing *PSD1* for 4 hours. Immunoblot analysis was used for detecting the mature and precursor forms of Mge1-FLAG in the mitochondrial and cytosolic fractions. Mitochondria were treated with 25 or 50 μg/ml proteinase K (Prot. K). Tom70-Myc served as an outer membrane control protein, Cox4 as a matrix control protein, and Pgk1 as a cytosolic control protein. OE, overexpression. (**f**) Immunoblot analysis of untagged Mge1 in cellular fractions from cells overexpressing *PSD1* for 4 hours. Htb1-mCherry and Pgk1serve as nuclear and cytosolic markers, respectively. Input and spheroplasts (Sphe.) represent total cell lysates, before and after cell wall digestion. Supernatant (Sup.) =post-nuclear supernatant; Nuc. Frac, nuclear fraction; p, precursor form; m, mature form. (**g**) Confirmation of *PSD1* overexpression by qPCR analysis for the samples used in Figure 2d. n=3 biological replicates; One-way ANOVA followed by Dunnett's test. (**a, d, g**) Data represent mean +/- SD.

Our data showed that overexpressed Mge1 can translocate to the nucleus and induce the mitoCPR. Yet, whether endogenous Mge1 has a similar function under conditions of impaired mitochondrial protein import remained unclear. We previously demonstrated that transient overexpression of the mitochondrial protein Psd1 does not affect membrane polarization but leads to the accumulation of mitochondrial precursors, suggesting that it inhibits protein import^21^. This inhibition was confirmed *in vitro* using isolated mitochondria, where *PSD1* overexpression (*PSD1^OE^*) led to a reduced import rate of Zim17 and Mge1 (Extended Data Fig. 3a-b).

**Fig. 2:**
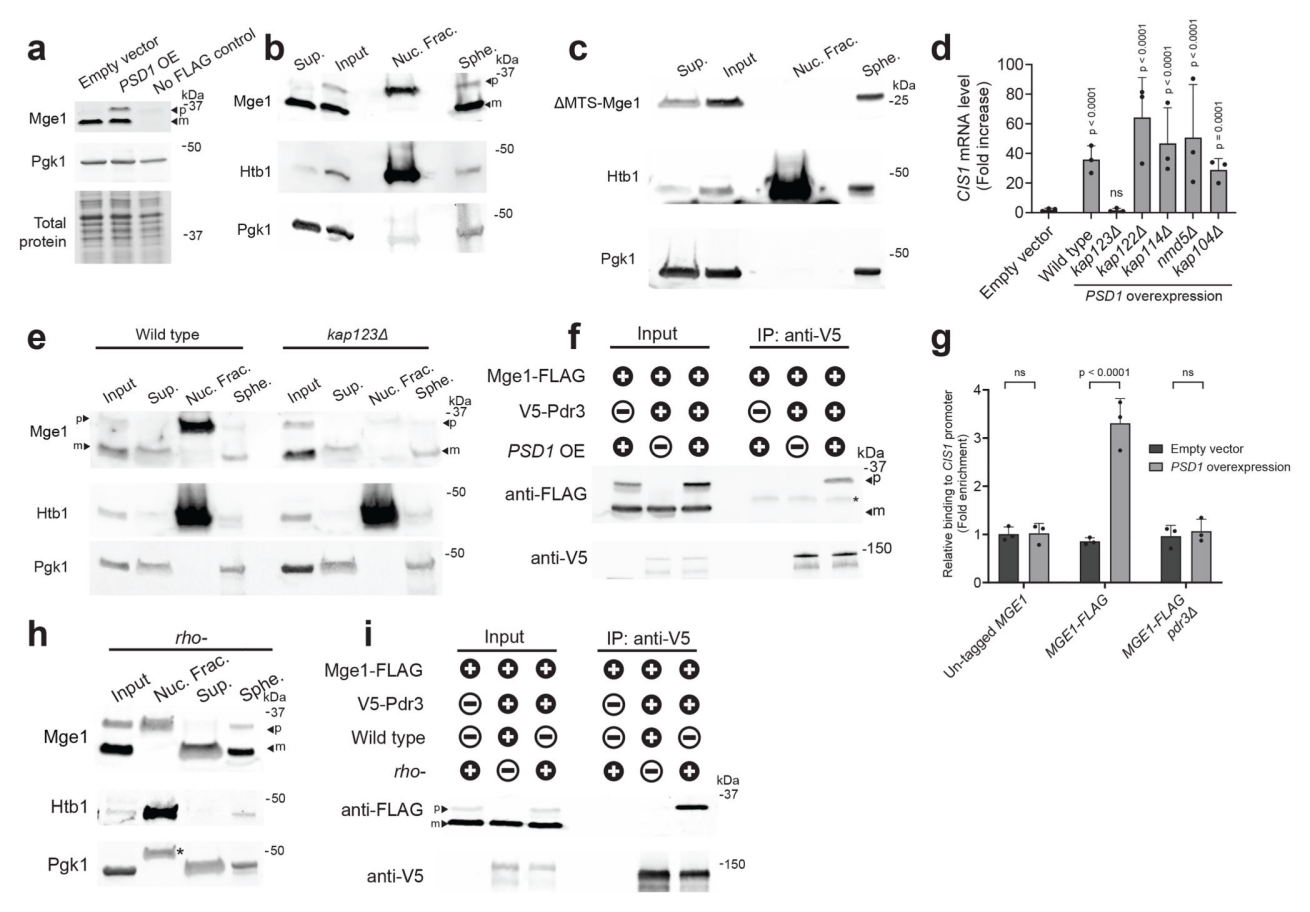
The endogenous Mge1 precursor translocates to the nucleus and binds pdr3. **(a)** Immunoblot of Mge1-FLAG in empty vector and *PSD1^OE^* cells. Cells with no FLAG tag served as a control. p, precursor; m, mature; OE, overexpression. (**b,c**) Immunoblot of Mge1-FLAG (b) and ΔMTS-Mge1-FLAG (c) in cellular fractions from *PSD1^OE^* cells (4 hours). Htb1-mCherry and Pgk1serve as nuclear and cytosolic markers, respectively. Input and spheroplasts (Sphe.) represent total cell lysates, before and after cell wall digestion. Supernatant (Sup.) =post-nuclear supernatant; Nuc. Frac, nuclear fraction; p, precursor; m, mature. (**d**) *CIS1* mRNA levels in wild-type and importin β-deletion strains under conditions with impaired protein import (*PSD1^OE^* for 4 hours). Wild-type strain under control (empty vector) conditions is included. n=3 biological replicates; One-way ANOVA followed by Dunnett's test. (**e**) same as in (b); untagged Mge1 was detected using Mge1 antiserum in wild-type and *kap123*Δ cells. (**f**) V5-Pdr3 immunoprecipitation from cells expressing *MGE1-FLAG* under control or protein import stress conditions (*PSD1^OE^*). The asterisk indicates a nonspecific band. OE, overexpression; p, precursor form; m, mature form. (**g**) ChIP analysis of cells expressing *MGE1* or *MGE1-FLAG* under control (empty vector) and protein import stress (*PSD1^OE^*) conditions. Mge1's association with the *CIS1* promoter was normalized over the *HMR* locus. n = 3 biological replicates; two-way ANOVA followed by Šídák's test. (**h**) Immunoblot of Mge1-FLAG in cellular fractions from *rho-* cells. Htb1-HA-turboID and Pgk1 serve as nuclear and cytosolic markers, respectively. Input and spheroplasts (Sphe.) represent total cell lysates, before and after cell wall digestion. Supernatant (Sup.) =post-nuclear supernatant; Nuc. Frac, nuclear fraction; p, precursor; m, mature. The asterisk indicates Htb1-HA signal residue on the Pgk1 blot. (**i**) Same as in (f). V5-Pdr3 was immunoprecipitated from wild-type and *rho-* cells. (**d, g**) Data represent mean +/- SD; ns, not significant.

We used the *PSD1^OE^* system to impair protein import and monitored the localization of the Mge1 precursor in cells. A FLAG tag was fused to the C-terminus of Mge1 at its endogenous locus, which did not affect the protein's import rate (Extended Data Fig. 3c). In control cells, only a single form of Mge1-FLAG was detected, corresponding to the size of the mature cleaved protein (Fig. 2a). As expected, *PSD1^OE^* led to the accumulation of Mge1-FLAG precursor (Fig. 2a and Extended Data Fig. 3d). To confirm that this precursor did not accumulate inside mitochondria, we performed cellular fractionation. In contrast to the mature form of Mge1, its precursor was not detected in the mitochondrial-enriched fraction (Extended Data Fig. 3e). Notably, the precursor, but not mature form of Mge1-FLAG was detected in nuclei isolated from *PSD1^OE^* cells (Fig. 2b). To exclude the possibility that the FLAG tag altered Mge1 localization, we repeated the analysis using untagged Mge1, which yielded similar results (Extended Data Fig. 3f). These findings suggest that the precursor of Mge1 can translocate into the nucleus when its mitochondrial import is impaired.

No canonical nuclear localization signal (NLS) was identified in Mge1 using established prediction tools^33^. We therefore tested whether the N-terminal MTS that targets Mge1 to the mitochondria is also required for its nuclear localization. Interestingly, unlike full-length Mge1, ΔMTS-Mge1-FLAG was absent from the nuclei of *PSD1^OE^* cells and detected in the non-nuclear fraction (Fig. 2c), suggesting that Mge1's MTS might be involved in facilitating its nuclear translocation. To further investigate how the Mge1 precursor translocates into the nucleus, we deleted several non-essential importin β genes. Importin β receptors, which mediate the import of most nuclear proteins, exhibit some redundancy in their cargo but also possess distinct cargo specificity^34^. While *PSD1^OE^* led to upregulation of *CIS1* in cells lacking *KAP114, KAP122, KAP104*, or *NMD5*, no upregulation was observed in *kap123*Δ cells (Fig. 2d and Extended Data Fig. 3g). Importantly, the precursor of Mge1 was barely detectable in the nuclei of *kap123*Δ cells (Fig. 2e), suggesting that Kap123 is required for the nuclear localization of Mge1 precursor.

**Extended Data Fig. 4:**
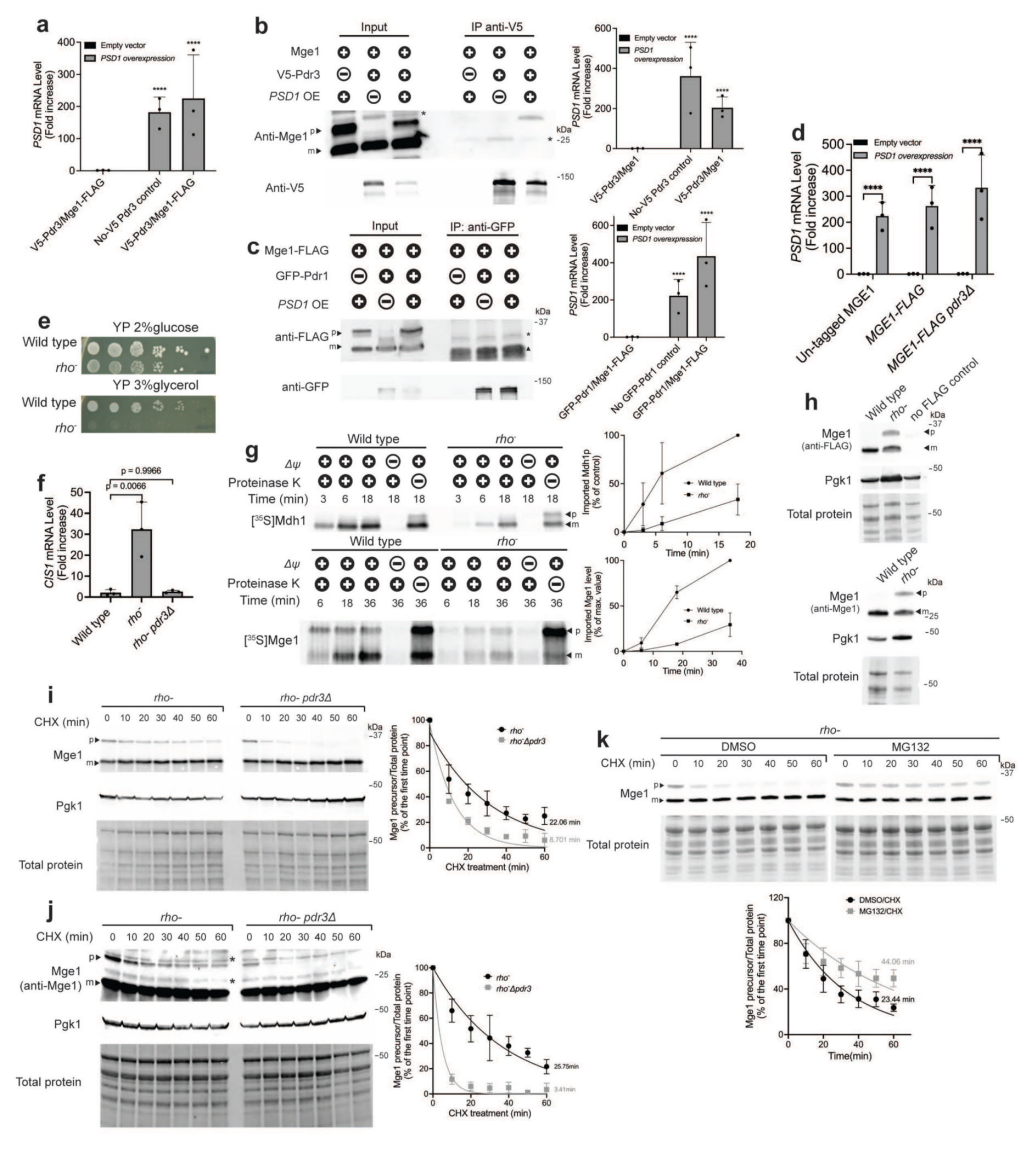
Mge1 precursor accumulates in the nucleus and not inside mitochondria when protein import is impaired. **(a**) Confirmation of *PSD1* overexpression by qPCR analysis for the samples used in Figure 2f. n=3 biological replicates; One-way ANOVA followed by Dunnett's test. (**b**) Cells expressing *P_TEF2_-V5-PDR3* and control no-V5 cells (*P_TEF2_-GFP-PDR3*) were incubated for 4 hours under control or protein import stress conditions (induced by *PSD1* overexpression). V5-Pdr3 was immunoprecipitated using V5-Trap beads. Untagged Mge1 was detected using Mge1 antiserum. The asterisks indicate nonspecific bands. OE, overexpression. p, precursor form. m, mature form. The corresponding *PSD1* mRNA levels are shown on the right panel. n=3 biological replicates; one-way ANOVA followed by Dunnett's test. (**c**) Cells expressing *MGE1-FLAG* and cells expressing *MGE1-FLAG* and *P_TEF2_-GFP-PDR1* were incubated for 4 hours under control or protein import stress conditions. GFP-Pdr1 was immunoprecipitated using anti-GFP antibodies coupled to IgG beads from cells expressing *P_TEF2_-GFP-PDR1*. The asterisk indicates a nonspecific band and ▲ indicates the GFP antibody's light chain. OE, overexpression; p, precursor form; m, mature form. The corresponding *PSD1* mRNA levels are shown on the right panel. n=3 biological replicates; one-way ANOVA followed by Dunnett's test. (**d**) Confirmation of *PSD1* overexpression by qPCR analysis for the samples used in Figure 2g. n=3 biological replicates; one-way ANOVA followed by Tukey's test. Serial dilutions of wild-type and *rho^-^* cells. Cells were grown to logarithmic phase and spotted on YP (1% yeast extract, 2% bactopeptone) plates supplemented by 2% glucose or 3% glycerol. (**f**) *CIS1* mRNA levels in wild-type and *rho^-^* cells in the presence of absence of *PDR3*. n=3 biological replicates; one-way ANOVA followed by Dunnett's test. (**g**) *In vitro* protein import assay using mitochondria isolated from wild-type and *rho-* cells. Mitochondria were incubated with radiolabeled ^35^S-Mdh1 and ^35^S-Mge1 for the indicated durations. Membrane potential (Δψ) was dissipated by preincubation with antimycin A, oligomycin, and valinomycin. Quantifications from three biological replicates are shown on the right; protein levels imported into wild-type mitochondria at the final time point were set to 100%. p, precursor; m, mature. (**h**) Immunoblot analysis of Mge1-FLAG and untagged Mge1 in wild-type and *rho*-cells. Wild-type cells that do not contain a FLAG tag were used as a control for Mge1-FLAG. p, precursor form. m, mature forms. (**i**) Half-life analysis of the Mge1-FLAG precursor in *rho^-^* and *PDR3*-deleted *rho^-^* cells. Cells were treated with cycloheximide (CHX, 0.5 mg/ml) and samples were collected at the indicated time points. Quantification of 4 biological replicates is shown on the right. p, precursor form. m, mature form. (**j**) Same as (i). Half-life analysis of the untagged Mge1 precursor in *rho^-^* and *PDR3* deleted *rho^-^* cells (n = 3 biological replicates). p, precursor form. m, mature form. Asterisks indicate nonspecific bands. (**k**) Same as in (i), *rho^-^* cells were treated with DMSO (vehicle control) or MG132 (40μM) for 1 hour prior to the addition of cycloheximide (n = 3 biological replicates). (**a-d, f, g, i-k**) Data represent mean +/- SD. (**a-d**) **** P ≤ 0.0001.

The nuclear localization of Mge1 suggested that it directly impacts the mitoCPR transcription regulator Pdr3. Co-immunoprecipitation revealed an interaction between V5-Pdr3 and the precursor, but not the mature form, of both FLAG-tagged and untagged Mge1 under protein import stress (Fig. 2f and Extended Data Fig. 4a-b). Notably, no interaction was observed with the paralog of Pdr3, Pdr1, which is dispensable for mitoCPR induction (Extended Data Fig. 4c)^21^. To investigate whether Mge1 can interact with DNA-bound Pdr3, we performed a ChIP assay by pulling down Mge1-FLAG. Mge1 associated with the *CIS1* promoter only under conditions of defective mitochondrial protein import (Fig. 2g and Extended Data Fig. 4d). Notably, this association was not detected in *pdr3*Δ cells, suggesting that Mge1's chromatin binding depends on its interaction with this transcription factor (Fig. 2g).

These findings were validated in an alternative yeast model with impaired mitochondrial protein import. Mutants with partially or fully deleted mitochondrial DNA (i.e., *rho^-^* or *rho^0^*) are respiration-deficient and exhibit reduced mitochondrial membrane potential and protein import defects (Extended Data Fig. 4e)^35^. As previously shown, the mitoCPR was constitutively activated in *rho*^-^ cells in a Pdr3-dependent manner (Extended Data Fig. 4f)^21,36^. *In organello* import assays confirmed reduced import efficiency for *rho^-^* mitochondria, including impaired import of Mge1 (Extended Data Fig. 4g). Consistently, both FLAG-tagged and untagged Mge1 precursors were detected in *rho^-^* but not wild-type cells (Extended Data Fig. 4h). Similarly to the *PSD1^OE^* system, Mge1 precursor was detected in isolated nuclei from *rho^-^* cells and co-immunoprecipitated with Pdr3 (Fig. 2h-i). Thus, Mge1 interacts with Pdr3 in both acute and chronic models with mitochondrial protein import defects.

We next used the *rho^-^* model to investigate whether Pdr3 binding stabilizes the Mge1 precursor. In *PDR3* expressing cells, Mge1 and Mge1-FLAG precursors exhibited a half-life of ~22–25 minutes (Extended Data Fig. 4i-j). The observed decline in precursor levels over time could result from either proteasomal degradation or mitochondrial import followed by presequence cleavage. To distinguish between these possibilities, we used the proteasome inhibitor MG132, which led to a twofold increase in precursor half-life (Extended Data Fig. 4k). This result suggests that at least a portion of the precursor population is targeted to proteasomal degradation Interestingly, in *PDR3-*deleted cells, Mge1 precursors were destabilized, exhibiting half-lives of less than 9 minutes (Extended Data Fig. 4i-j). These results suggest that binding to Pdr3 protects Mge1 from degradation. Together, our data show that Mge1 can enter the nucleus during mitochondrial stress and interact with Pdr3 on DNA regulatory elements.

## Mge1 is essential for Pdr3 activation

**Extended Data Fig. 5:**
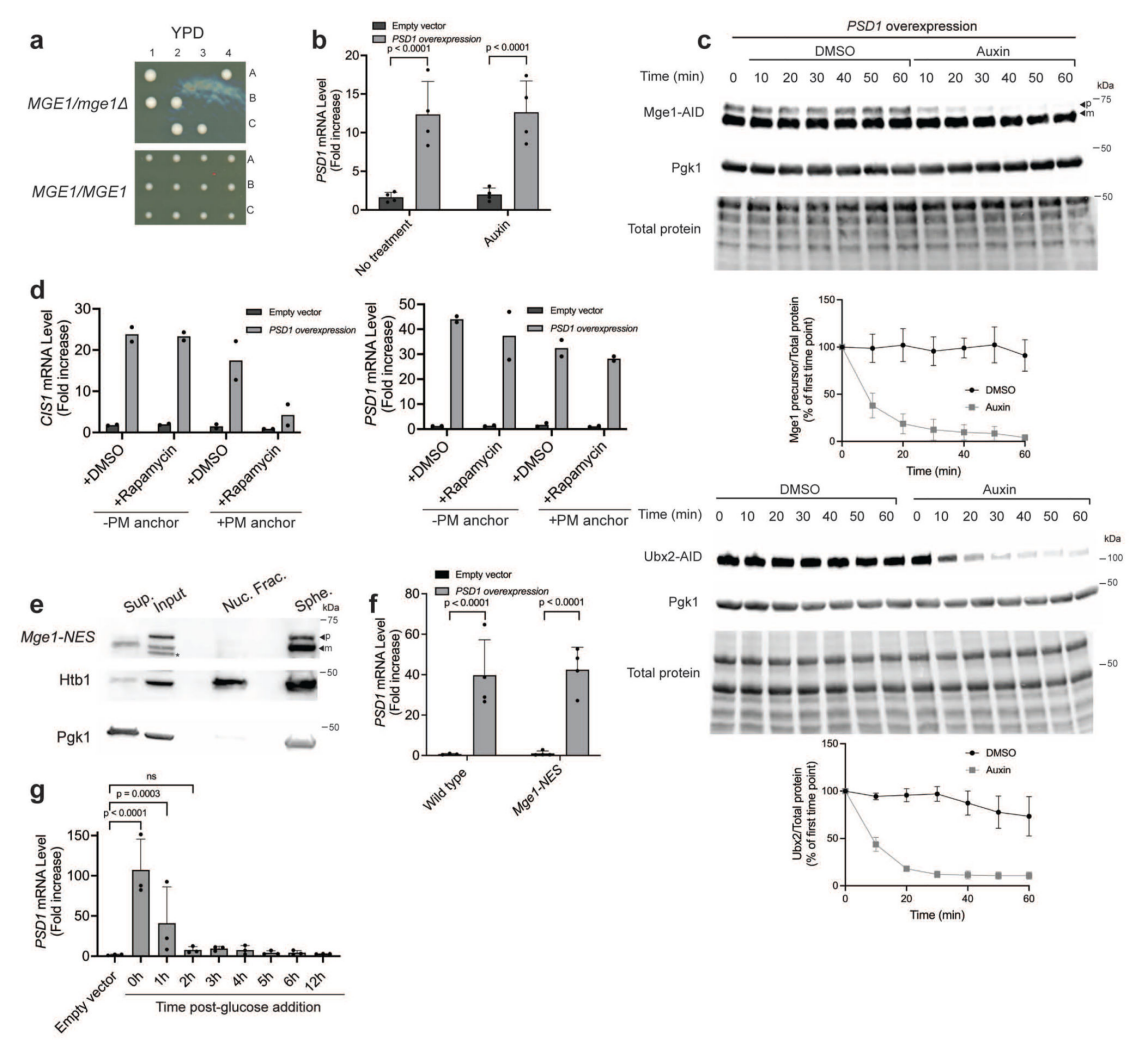
Activation of the mitoCPR requires the presence of Mge1 precursor in the nucleus. (**a**) Tetrad dissection of wild-type and *MGE1/mge1*Δ diploid strains showing that haploid yeast deleted of *MGE1* are inviable. (**b**) Confirmation of *PSD1* overexpression by qPCR analysis for the samples used in Figure 3a-b. n=4 biological replicates; one-way ANOVA followed by Tukey's test. (**c**) Cells expressing Mge1-Myc-AID or Ubx2-Myc-AID were treated with DMSO or auxin (1mM indole-3-acetic acid) following 4 hours induction of *PSD1* overexpression by galactose. Samples were collected at the indicated time points. Levels of the Mge1 precursor as well as Ubx2 were quantified from 3 biological replicates. (**d**) *CIS1* and *PSD1* mRNA levels in cell expressing *MGE1-FRB* with or without the plasma membrane (PM) anchor Pil1-FKBP under control or import stress conditions (*PSD1* overexpression for 4 hours). Anchoring of the Mge1 precursor was induced by adding 50nM rapamycin for the duration of *PSD1* overexpression. n=2 biological replicates; data represent mean; one-way ANOVA followed by Dunnett's test; ns, not significant. (**e**) Immunoblot analysis of Mge1-NES-mCherry in cellular fractions from cells overexpressing *PSD1* for 4 hours. Htb1-mCherry and Pgk1serve as nuclear and cytosolic markers, respectively. Input and spheroplasts (Sphe.) represent total cell lysates, before and after cell wall digestion. Supernatant (Sup.)=post-nuclear supernatant. The asterisk indicates a band likely to be a degradation product. p, precursor form. m, mature form. (**f**) Confirmation of *PSD1* overexpression by qPCR analysis for the samples used in Figure 3c. n=4 biological replicates; one-way ANOVA followed by Tukey's test. (**g**) *PSD1* overexpression was induced by galactose and terminated following 4 hours by glucose addition. *PSD1* mRNA levels were monitored over 12 hours post-glucose addition (same samples as in Fig. 3e). n=3 biological replicates; one-way ANOVA followed by Dunnett's test; ns, not significant. (**b, c, f, g**) Data represent mean +/- SD.

**Fig. 3:**
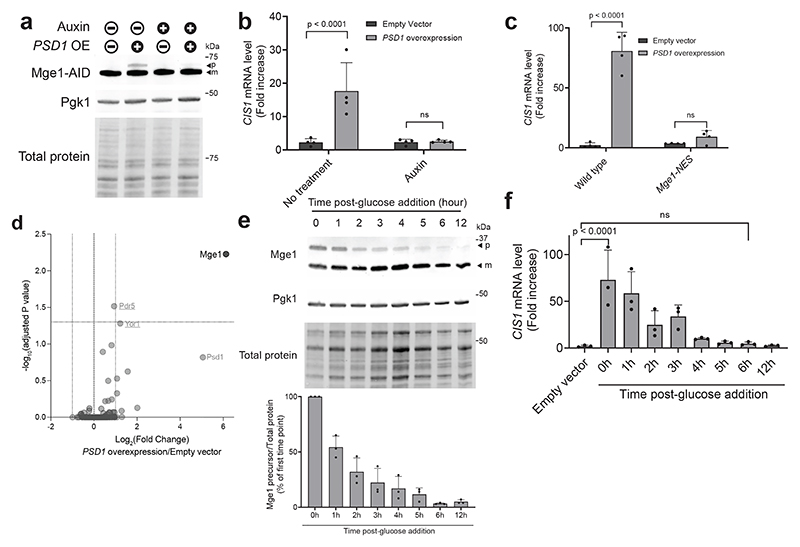
Mge1 is essential for mitoCPR activation. **(a)** Immunoblot of Mge1-Myc-AID under control (empty vector) and protein import stress (*PSD1^OE^*) conditions. Degradation of Mge1 was induced by auxin treatment (1mM indole-3-acetic acid) for one hour followed by 4 hours of *PSD1^OE^* in the presence of auxin. p, precursor. m, mature. OE, overexpression. (**b**) Analysis of *CIS1* mRNA levels in the same strains and conditions as in (a). n=4 biological replicates; one-way ANOVA followed by Tukey's test. (**c**) Analysis of *CIS1* mRNA levels in cells expressing Mge1-FLAG or Mge1-NES-FLAG under control (empty vector) and protein import stress conditions (*PSD1^OE^* for 4 hours). n=4 biological replicates; one-way ANOVA followed by Tukey's test. (**d**) V5-Pdr3 was immunoprecipitated using V5-Trap beads and the samples were analyzed by mass spectrometry. The volcano plot displays high-confidence Pdr3 candidate interactors, comparing stressed (*PSD1^OE^*) and non-stressed control (empty vector) conditions. Two-tailed t-test followed by Šidák test. Pdr3-targets which are close to the cutoff values are underlined. (**e,f**) *PSD1* overexpression was induced by galactose and terminated following 4 hours by glucose addition. Mge1-FLAG precursor (e) and *CIS1* mRNA levels (f; One-way ANOVA followed by Dunnett's test) were monitored over 12 hours post-glucose addition. n=3 biological replicates. OE, overexpression; p, precursor; m, mature. (**b, c, e, f**) Data represent mean +/- SD; ns, not significant.

Our results demonstrate that unimported Mge1 binds Pdr3, however they do not exclude the possibility that Mge1 is one of several precursors that act redundantly to induce the mitoCPR. To address this, we tested whether Mge1 is required for mitoCPR activation under protein import stress conditions. Since *MGE1* is an essential gene (Extended Data Fig. 5a)^32^, we utilized the auxin-inducible degron (AID) system. Under basal conditions, only the mature form of endogenously expressed Mge1-AID was detected, whereas inhibition of protein import resulted in accumulation of its precursor (Fig. 3a and Extended Data Fig. 5b). Auxin treatment eliminated most of the Mge1-AID precursor, similar to the unrelated Ubx2-AID control protein (Fig. 3a and Extended Data Fig. 5c). Mature Mge1-AID remained detectable following this treatment, consistent with the fact that proteasomal degradation does not occur inside mitochondria and therefore affects only precursors on their way to the organelles (Fig. 3a). Importantly, depletion of Mge1 significantly impaired *CIS1* upregulation in response to protein import stress (Fig. 3b and Extended Data Fig. 5b). A similar defect in mitoCPR activation was observed when the Mge1 precursor was sequestered at the plasma membrane using the anchor-away system (Extended Data Fig. 5d). To further test whether nuclear localization of Mge1 is essential for mitoCPR activation, we fused a nuclear export signal (NES) to Mge1, which prevented its nuclear accumulation (Extended Data Fig. 5e). Cells expressing this non-nuclear variant failed to induce *CIS1* transcription upon *PSD1^OE^* (Fig. 3c and Extended Data Fig. 5f).

To examine whether other mitochondrial precursors bind Pdr3, we characterized its interactome by mass spectrometry following immunoprecipitation. Stress-specific interactors were identified by comparing Pdr3-containing complexes from basal and protein import stress conditions. Mge1 was the only stress-specific interactor significantly enriched in these complexes, with an increase of more than twofold (Fig. 3d and Supplementary Table 2). Enrichment just below the cutoff was observed for proteins encoded by Pdr3-target genes, likely reflecting their increased abundance in whole-cell lysates (Fig. 3d and Supplementary Table 2). Psd1, which was overexpressed to induce mitochondrial damage, was also detected, although its enrichment did not reach the significance threshold. This analysis suggests that Mge1, but not other mitochondrial precursors, specifically interacts with Pdr3.

Finally, we used the galactose-inducible *PSD1^OE^* system to monitor Mge1 precursor during recovery from stress. Upon glucose addition, which terminated *PSD1* overexpression (Extended Data Fig. 5g), we observed a gradual decrease in both Mge1 precursor and *CIS1* mRNA levels within 6 hours (Fig. 3e-f). This result indicates that the *PSD1^OE^*-induced accumulation of Mge1 precursor is reversible.

## Mge1's MTS signals mitochondrial stress

**Extended Data Fig. 6:**
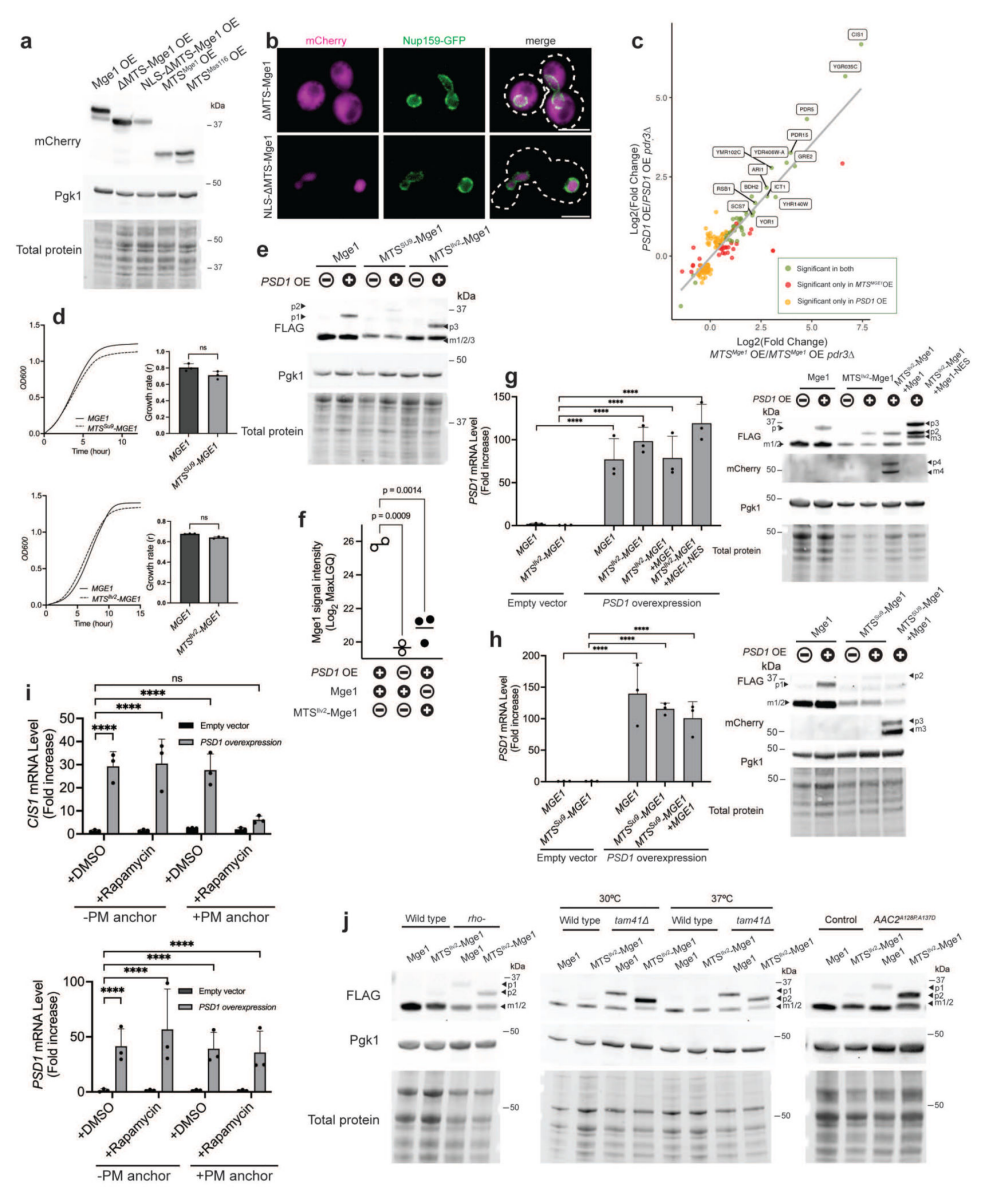
Mge1's presequence is required for inducing the mitoCPR. (**a**) Immunoblot of overexpressed mCherry-tagged Mge1 truncation mutants and the Mss116's MTS (4 hours following galactose induction). OE, overexpression. (**b**) Live-cell fluorescence images of cells expressing the nuclear envelope marker Nup159-GFP and ΔMTS-Mge1-mCherry or NLS-ΔMTS-Mge1-mCherry. Expression of *MGE1* variants from the *GAL1-10* promoter was induced by the addition of galactose for 4 hours. Scale bars, 5 μm. (**c**) Pdr3-dependent upregulated genes were identified by differential gene expression analysis of: 1. wild-type versus *pdr3*Δ cells, both overexpressing *MTS^Mge1^-mCherry*, and 2. wild-type versus *pdr3*Δ cells, both overexpressing *PSD1*. The scatter plot displays genes with an adjusted p value ≤ 0.05 (Wald test in *DESeq2)* in at least one of analyses. *PDR3* was removed from both datasets prior to the correlation analysis. (**d**) Growth curves of wild-type, *MTS^Su9^-MGE1*, and *MTS^Ilv2^-MGE1* cells were plotted on a semilogarithmic scale. Corresponding growth rate (r) was calculated from the growth curves of three biological replicates, each with three technical replicates; two-tailed t-test. ns, not significant. (**e**) Immunoblot of cells expressing Mge1-FLAG, MTS^Su9^-Mge1-FLAG, or MTS^Ilv2^-Mge1-FLAG under control and *PSD1* overexpression conditions. p1, Mge1-FLAG precursor; p2, MTS^Su9^-Mge1-FLAG precursor; p3, MTS^Ilv2^-Mge1-FLAG precursor. m1/2/3, mature form Mge1-FLAG/MTS^Su9^-Mge1-FLAG/MTS^Ilv2^-Mge1-FLAG (**f**) V5-Pdr3 was immunoprecipitated using V5-trap beads from the following: 1. cells under basal conditions (n=2 biological repeats), 2. cells under impaired mitochondrial protein import conditions (*PSD1* overexpression; n=2 biological repeats), or 3. *MTS^Ilv2^-MGE1* cells under impaired mitochondrial protein import conditions (n=3 biological repeats). Mge1 intensities were measured by mass spectrometry analysis. Data are shown as mean; one-way ANOVA followed by Dunnett's test. (**g**) Left- Confirmation of *PSD1* overexpression by qPCR analysis for the samples used in Figure 4e. n=3 biological replicates; one-way ANOVA followed by Tukey's test. Right- Immunoblot of Mge1-FLAG and MTS^Ilv2^-Mge1-FLAG under control or import stress (*PSD1* overexpression for 4 hours) conditions. Addbacks of Mge1-mCherry and Mge1-NES-FLAG into the *MTS^Ilv2^-MGE1* background are also presented. p1, Mge1-FLAG precursor; p2, MTS^Ilv2^-Mge1-FLAG precursor; p3, Mge1-NES-FLAG precursor; p4, Mge1-mCherry precursor; m1/2, mature Mge1-FLAG/MTS^Ilv2^-Mge1-FLAG; m3, mature Mge1-NES-FLAG; m4, mature Mge1-mCherry. OE, overexpression. (**h**) Left- Confirmation of *PSD1* overexpression by qPCR analysis for the samples used in Figure 4f. n=3 biological replicates; one-way ANOVA followed by Tukey's test. Right- Immunoblot of Mge1-FLAG and MTS^Su9^-Mge1-FLAG under control or import stress (*PSD1* overexpression for 4 hours) conditions. Addback of Mge1-mCherry into the *MTS^Su9^-MGE1* background is also presented. p1, Mge1-FLAG precursor; p2, MTS^Su9^-Mge1-FLAG precursor; p3, Mge1-mCherry precursor; m1/2, mature Mge1-FLAG/MTS^Su9^-Mge1-FLAG; m3, mature Mge1-mCherry. OE, overexpression. (**i**) *CIS1* and *PSD1* mRNA levels in cell expressing *MTS^Ilv2^-MGE1-FLAG* and *MGE1-FRB* with or without the plasma membrane (PM) anchor Pil1-FKBP. Cells were grown under control or import stress (*PSD1* overexpression for 4 hours) conditions. Anchoring of the Mge1 precursor was induced by adding 50nM rapamycin for the duration of *PSD1* overexpression. n=3 biological replicates; one-way ANOVA followed by Tukey's test. (**j**) Immunoblot of Mge1-FLAG and MTS^Ilv2^-Mge1-FLAG in wild-type cells, *rho-* cells, *tam41*Δ cells (at both 30°C and 37°C), and cells expressing *aac2^A128P, A137D^* for 4 hours. p1, Mge1-FLAG precursor; p2, MTS^Ilv2^-Mge1-FLAG precursor; m1/2, mature form Mge1-FLAG/MTS^Ilv2^-Mge1-FLAG. (**d, g-i**) Data represent mean +/- SD; ns, not significant; **** P ≤ 0.0001.

**Fig. 4:**
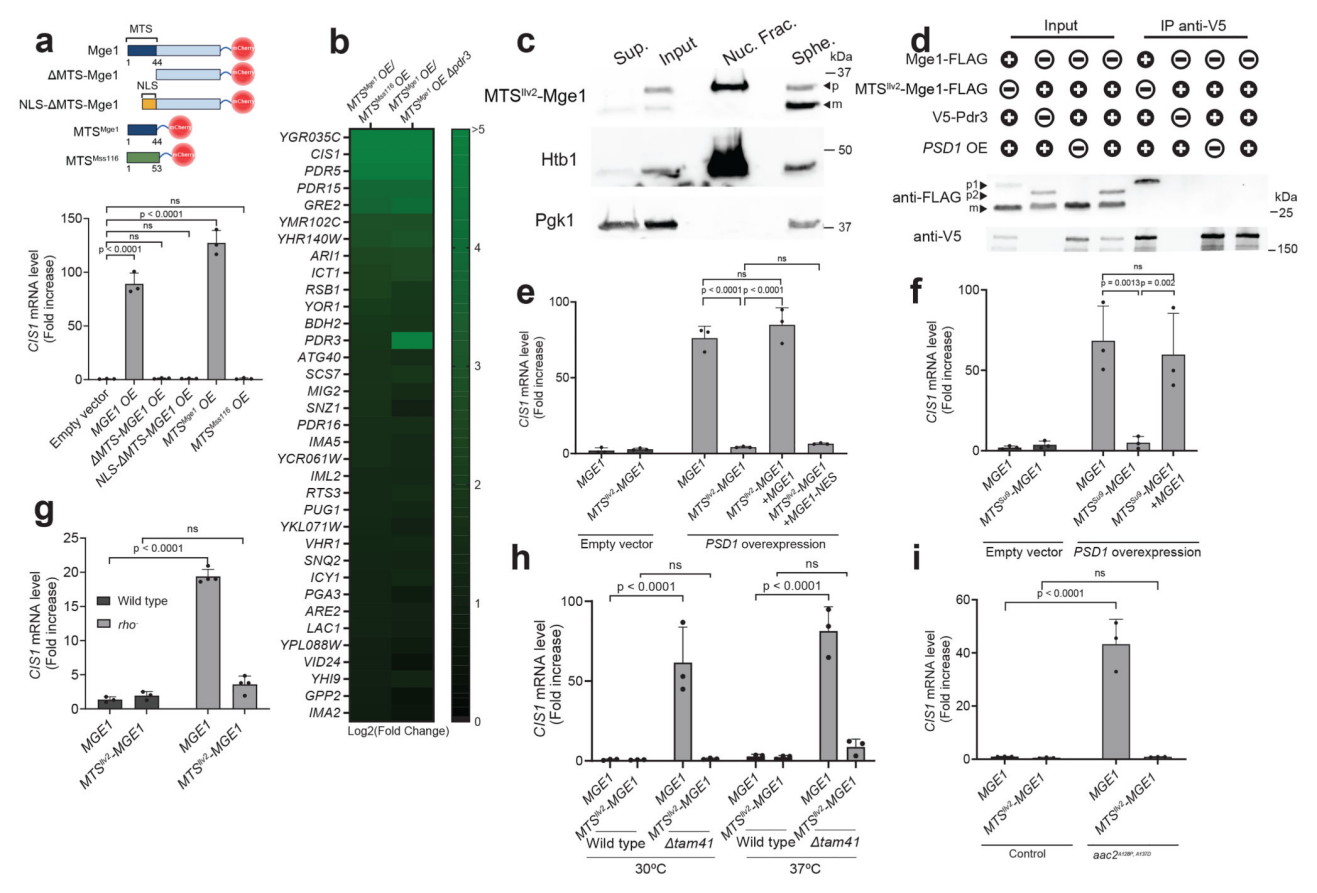
The presequence of Mge1 functions in mitochondria-to-nucleus signaling. (**a**) *CIS1* mRNA levels in empty vector cells and in cells overexpressing Mge1 truncation mutants or the MTS^Mss116^ (4 hours). n=3 biological replicates; one-way ANOVA followed by Dunnett's test. OE, overexpression. (**b**) Heatmap of genes upregulated (log2 fold change ≥ 1, adjusted p value ≤ 0.05; Wald test in *DESeq2*) in cells overexpressing *MTS^Mge1^-mCherry* compared to *MTS^Mss116^-mCherry*. The differential expression of these genes in wild-type versus *pdr3*Δ backgrounds overexpressing *MTS^Mge1^-mCherry* is shown on the right. Dubious ORFs and *MGE1* were excluded from the analysis. OE, overexpression. (**c**) Immunoblot analysis of MTS^Ilv2^-Mge1-FLAG in cellular fractions from *PSD1^OE^* cells. Htb1-mCherry and Pgk1serve as nuclear and cytosolic markers, respectively. Input and spheroplasts (Sphe.) represent total cell lysates, before and after cell wall digestion. Supernatant (Sup.) =post-nuclear supernatant; Nuc. Frac, nuclear fraction; p, precursor; m, mature. (**d**) V5-Pdr3 immunoprecipitation from cells expressing either *MGE1-FLAG* or *MTS^Ilv2^-MGE1-FLAG* grown under control and *PSD1^OE^* (4 hours) conditions. p1, Mge1-FLAG precursor. p2, MTS^Ilv2^-Mge1-FLAG precursor. m, mature. (**e**) *CIS1* mRNA levels in cells expressing wild-type *MGE1* and *MTS^Ilv2^-MGE1*, under control (empty vector) and *PSD1^OE^* (4 hours) conditions. *MTS^Ilv2^-MGE1* cells were reintroduced with wild-type *MGE1-mCherry* (*MTS^Ilv2^-MGE1*+*MGE1*) or *MGE1-NES-FLAG* (*MTS^Ilv2^-MGE1*+*MGE1-NES*). n=3 biological replicates; one-way ANOVA followed by Tukey's test. (**f**) same as (e) in *MTS^SU9^-MGE1* cells. (**g,h**) *CIS1* mRNA levels in *MGE1* or *MTS^Ilv2^-MGE1* cells in wild-type, *rho^-^* (g), or *tam41*Δ (h) backgrounds (analyzed at 30°C or 37°C for *tam41*Δ). n=3 biological replicates; one-way ANOVA followed by Tukey's test. (**i**) *CIS1* mRNA levels in *MGE1* or *MTS^Ilv2^-MGE1* cells carrying an empty vector or expressing the *aac2^A128P, A137D^* mutant (4 hours). n=3 biological replicates; one-way ANOVA followed by Tukey's test. (**a, e-i**) Data represent mean +/- SD; ns, not significant.

Next, we asked which region of Mge1 is responsible for activating Pdr3. The Mge1 precursor includes both the presequence peptide, which mediates mitochondrial import, and the mature protein which functions as an Hsp70 co-chaperone^32^. We first tested the ability of Mge1 mutants to activate the mitoCPR via overexpression. To determine whether the region corresponding to the mature Mge1 is responsible for Pdr3 activation, we overexpressed a ΔMTS-Mge1 truncated mutant. Unlike overexpression of full-length Mge1, which was sufficient to induce the mitoCPR, ΔMTS-Mge1 overexpression did not trigger this response (Fig. 4a and Extended Data Fig. 6a).

Consistent with the lack of nuclear accumulation observed for endogenously expressed ΔMTS-Mge1 (Fig. 2c), the overexpressed mutant displayed a diffuse cytoplasmic pattern rather than distinct nuclear localization (Extended Data Fig. 6b). To verify that ΔMTS-Mge1's inability to induce the mitoCPR did not stem from insufficient nuclear accumulation, we fused this mutant to a nuclear localization signal (NLS-ΔMTS-Mge1). The NLS restored nuclear localization but not *CIS1* induction, suggesting that Mge1's co-chaperone activity is not sufficient for mitoCPR signaling (Fig. 4a and Extended Data Fig. 6a-b). Interestingly, overexpression of the MTS of Mge1 alone (residues 1-44) fused to mCherry, but not that of Mss116 (residues 1-53), induced *CIS1* expression (Fig. 4a and Extended Data Fig. 6a).

To fully characterize the response triggered by the Mge1 presequence, we analyzed the transcriptome of cells overexpressing MTS^Mge1^-mCherry. To identify genes specifically affected by this MTS, we compared the transcriptomes of cells overexpressing MTS^Mge1^-mCherry and the control MTS^Mss116^-mCherry. A total of 35 genes were differentially upregulated by at least twofold in response to MTS^Mge1^-mCherry overexpression, all in a Pdr3-dependent manner, suggesting that Pdr3 is the primary transcriptional activator affected by this presequence (Fig. 4b and Supplementary Table 3-4). A comparison of the Pdr3-dependent transcriptional responses triggered by overexpression of MTS^Mge1^-mCherry and Psd1 showed a strong correlation across both conditions (Extended Data Fig. 6c and Supplementary Tables 4-5). Thus, MTS^Mge1^-mCherry is sufficient to induce the full repertoire of mitoCPR target genes.

To further investigate whether Mge1's MTS is indispensable for mitoCPR activation, we eliminated this peptide from the yeast genome. As Mge1's activity in the mitochondria is essential^32^, the import of this protein is required for cell viability. To preserve Mge1's mitochondrial targeting, we replaced its MTS with either the MTS of: 1. *Neurospora crassa* ATP-synthase subunit 9 (Su9), or 2. Ilv2, a mitochondrial protein reported to localize to the nucleus when not imported^18^. These chimera strains exhibited growth kinetics comparable to wild-type yeast (Extended Data Fig. 6d). Upon *PSD1^OE^*, the MTS^Ilv2^-Mge1 precursor accumulated, whereas the MTS^Su9^-Mge1 precursor did not, presumably due to the strong import efficiency of the Su9 MTS, rapid degradation, or both (Extended Data Fig. 6e)^37^. Thus, the *MTS^Su9^-MGE1* allele is not suitable for testing whether the Mge1 precursor requires its native MTS to bind and activate Pdr3.

To investigate whether MTS^Ilv2^-Mge1 can induce the mitoCPR, we first demonstrated that its precursor was detectable in isolated nuclei, suggesting that the MTS of Ilv2 can direct Mge1 to the nucleus (Fig. 4c). Notably, despite its nuclear localization, MTS^Ilv2^-Mge1 precursor did not interact with Pdr3 and failed to induce *CIS1* expression in response to *PSD1^OE^* (Fig. 4d-e and Extended Data Fig. 6f-g). As expected, *CIS1* upregulation was also blocked in *MTS^Su9^-MGE1* cells (Fig. 4f and Extended Data Fig. 6h). These data demonstrate that both *MTS^Ilv2^-MGE1* and *MTS^Su9^-MGE1* can be used as separation-of-function alleles that preserve cell viability but not Mge1-mediated activation of the mitoCPR. Reintroduction of a wild-type *MGE1* copy into the separation-of-function strains rescued the response to impaired protein import, demonstrating that Pdr3 function remained intact in these backgrounds (Fig. 4e-f and Extended Data Fig. 6g-h). In contrast, addbacks of *MGE1-NES* or plasma membrane-anchored *MGE1* failed to trigger the mitoCPR under stress conditions (Fig. 4e and Extended Data Fig. 6g and 6i).

Finally, we validated the role of Mge1's MTS in stress signaling by employing the *MTS^Ilv2^-MGE1* allele across multiple yeast models of impaired mitochondrial protein import. In addition to *rho-* cells, we used a deletion of *TAM41*, which encodes a CDP-diacylglycerol synthase required for cardiolipin biosynthesis and proper function of the *TIM23* translocase^38–40^. Reduced membrane potential and protein import rate are features of *tam41*Δ cells particularly at elevated temperatures^39,41^. We also included a model with inducible expression of the pathogenic *AAC2/ANT1* allele *aac2*^A128P, A137D^, a mutant of a mitochondrial carrier protein that arrests in TOM and inhibits protein import^23^. We detected the precursor form of Mge1 in all three models (Extended Data Fig. 6j). The mitoCPR has been previously found to be constitutively active in *rho^-^* and *tam41*Δ cells^21,36^. Indeed, *CIS1* mRNA levels were elevated in these models compared to wild-type cells as well as upon expression of *aac2*^A128P, A137D^ (Fig. 4g-i). However, mitoCPR activation was abolished in the *MTS^Ilv2^-MGE1* background in these three models, despite the presence of the MTS^Ilv2^-Mge1 precursor (Fig. 4g-i and Extended Data Fig. 6j). Altogether, these data indicate that the MTS^Mge1^ peptide is essential for mitochondria-to-nucleus signaling in response to both acute and chronic mitochondrial protein import defects.

## Defining essential features of MTS^Mge1^

**Extended Data Fig. 7:**
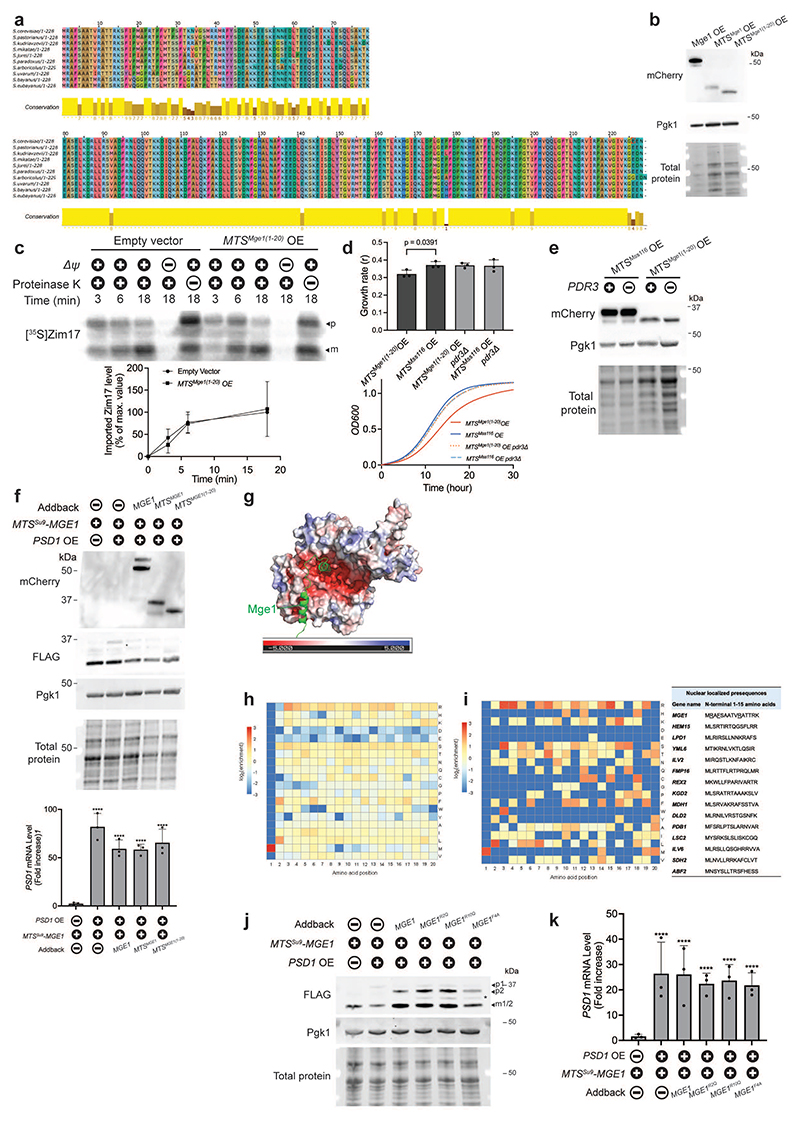
The N-terminus of Mge1 contains critical features for the mitoCPR activation. (**a**) Amino acid alignment and sequence conservation of Mge1 across 10 *Saccharomyces* species. (**b**) Immunoblot of overexpressed full-length and truncated Mge1-mCherry variants (4 hours galactose induction). OE, overexpression. (**c**) *In vitro* protein import assay using mitochondria isolated from control cells (empty vector) and cells overexpressing MTS^Mge1(1-20)^-mCherry for 4 hours. Mitochondria were incubated with radiolabeled ^35^S-Zim17 for the indicated durations. Membrane potential (Δψ) was dissipated by preincubation with antimycin A, oligomycin, and valinomycin. Quantification from three biological replicates is shown in the bottom panel; protein levels imported into control mitochondria at the final time point were set to 100%. p, precursor; m, mature. OE, overexpression. (**d**) Growth curves of cells overexpressing *MTS^116^-mCherry* or *MTS^Mge1(1-20)^-mCherry*, in the presence or absence of *PDR3*, were plotted on a semilogarithmic scale. Corresponding growth rate (r) was calculated from the growth curves of three biological replicates, each with three technical replicates; two-tailed t-test. OE, overexpression. (**e**) Immunoblot of overexpressed MTS^116^-mCherry and MTS^Mge1(1-20)^-mCherry 4 hours following galactose addition. OE, overexpression. (**f**) Upper panel- Immunoblot of the endogenously expressed addbacks (full-length Mge1-mCherry, MTS^Mge1^-mCherry, and MTS^Mge1(1-20)^-mCherry) into the *MTS^SU9^-MGE1-FLAG* background. Lower panel- Confirmation of *PSD1* overexpression by qPCR analysis for the samples used in the upper panel and in Figure 5c. n=3 biological replicates; one-way ANOVA followed by Tukey's test. OE, overexpression. (**g**) The electrostatic surface potential plot shows that the presequence of Mge1(residues 1-60, in green) fits into a negatively charged patch in Pdr3 (residues 86-856). (**h-i**) Heatmap of the amino acid enrichment across the first twenty amino acid of the MTSs from all yeast mitochondrial proteins (h) or previously reported nuclear-localized mitochondrial proteins (i). The enrichment score represents the frequency of a residue at a given position across the set weighted by the genome-wide amino acid frequencies. A table listing the N-terminal twenty amino acids of the mitochondrial presequences used in (i) is shown on the right. (**j**) Immunoblot of FLAG-tagged wild-type and mutants Mge1, expressed from the native *MGE1* promoter, in the *MTS^SU9^-MGE1-FLAG* background. The asterisk indicates a likely degradation product. p1, MTS^Su9^-Mge1-FLAG precursor; p2, Mge1-FLAG variants precursor; m1/2, mature form MTS^Su9^-Mge1-FLAG/Mge1-FLAG variants. OE, overexpression. (**k**) Confirmation of *PSD1* overexpression by qPCR analysis for the samples used in Figure 5f. n=3 biological replicates; one-way ANOVA followed by Tukey's test. OE, overexpression. (**c, d, f, k**) Data represent mean +/- SD; **** P ≤ 0.0001.

**Fig. 5:**
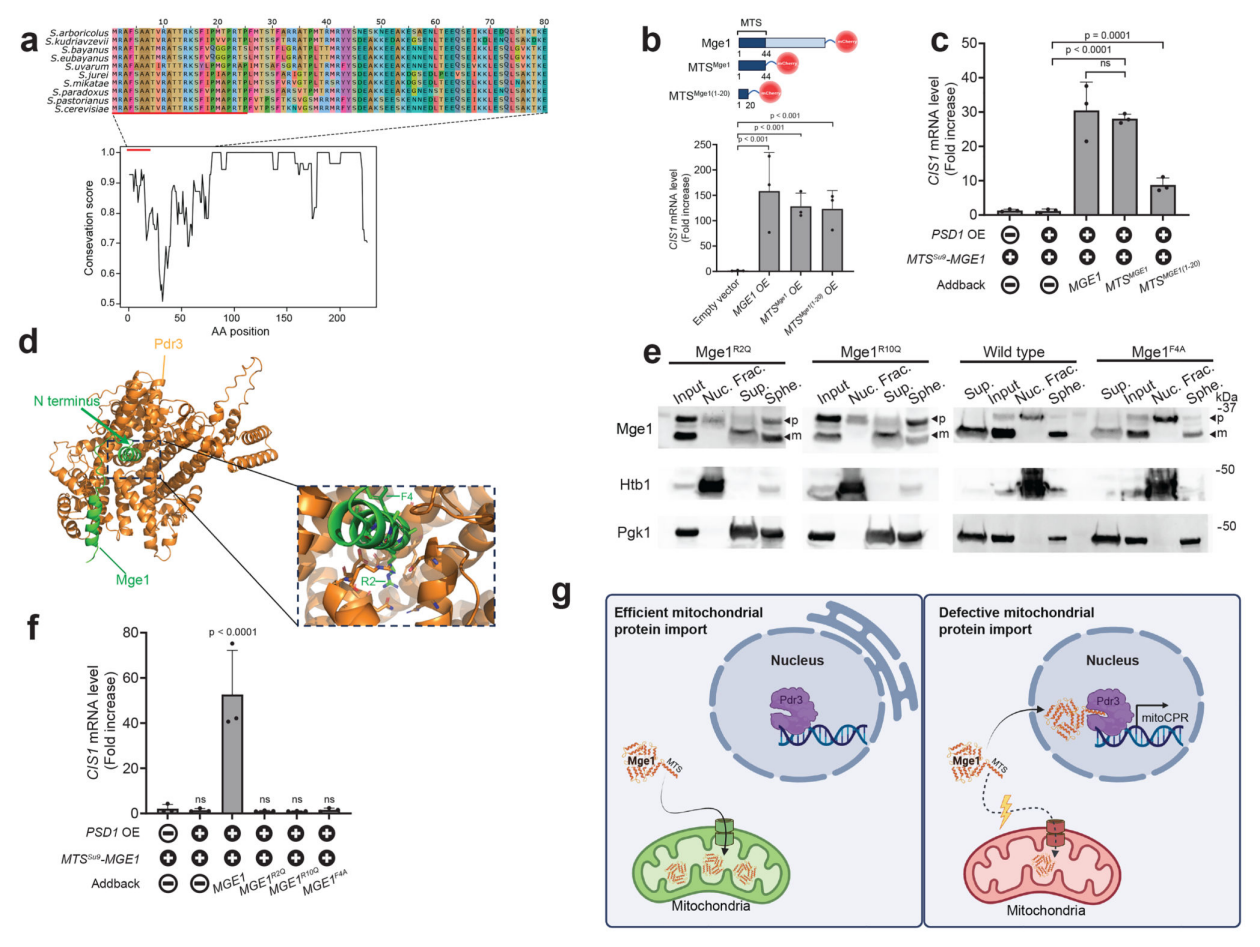
Specific residues in the Mge1's presequence are critical for mitoCPR activation. (**a**) Sequence conservation of Mge1 in *Saccharomyces* species. Bottom- Sliding window (5 amino acids) conservation score for full-length Mge1. Top- Amino acid alignment of the first 80 amino acids (AA). The red line represents the conserved N-terminus. (**b**) *CIS1* mRNA levels in empty vector cells or cells overexpressing *MGE1-mCherry, MTS^Mge1^-mCherry*, or *MTS^Mge1(1-20)^-mCherry* (4 hours). n=3 biological replicates; one-way ANOVA followed by Dunnett's test; OE, overexpression. (**c**) *CIS1* mRNA levels in *MTS^SU9^-MGE1-FLAG* cells under control (empty vector) or *PSD1^OE^* (4 hours) conditions, in the presence or absence of *MGE1-mCherry, MTS^Mge1^-mCherry*, or *MTS^Mge1(1-20)^-mCherry* (under the native *MGE1* promoter). n=3 biological replicates; one-way ANOVA followed by Tukey's test; OE, overexpression. (**d**) AlphaFold3 modeling of the interaction between Pdr3 (residues 86-856, in orange) and the N-terminus of Mge1 (residues 1-60, in green). (**e**) Wild-type and mutants Mge1 in cellular fractions from *PSD1^OE^* cells (4 hours). The *MGE1* genes were expressed from their native promoter in *MTS^Ilv2^-MGE1-mCherry* cells. Htb1-mCherry and Pgk1 serve as nuclear and cytosolic markers, respectively. Input and spheroplasts (Sphe.) represent total cell lysates, before and after cell wall digestion. Supernatant (Sup.) =post-nuclear supernatant; Nuc. Frac, nuclear fraction; p, precursor; m, mature. (**f**) No addback or addback of wild-type or mutant *MGE1-FLAG* (expressed from the *MGE1* promoter) were introduced into *MTS^SU9^-MGE1-FLAG* cells. *CIS1* mRNA levels were analyzed in empty vector and *PSD1^OE^* (4 hours) cells. n=3 biological replicates; one-way ANOVA followed by Dunnett's test; OE, overexpression. (**g**) A model for mitoCPR activation mechanism. Under basal conditions, the mitochondrial targeting sequence (MTS) of Mge1 mediates its import into the mitochondria. Under conditions with impaired import stress, Mge1 is translocated into the nucleus, where its MTS binds and activates Pdr3. (**b, c, f**) Data represent mean +/- SD; ns, not significant.

A conservation analysis revealed that, unlike the well-conserved mature protein, the presequence of Mge1 is poorly conserved even within *Saccharomyces* species (Fig. 5a and Extended Data Fig. 7a). Yet, a sliding-window analysis (five–amino acid window) identified higher homology across the first 25 residues of the MTS, suggesting a key functional role for this region (Fig. 5a and Extended Data Fig. 7a). Indeed, overexpression of Mge1's 20 N-terminal residues (*MTS^Mge1(1-20)^-mCherry*) induced *CIS1* expression to a similar extent as the full-length MTS (Fig. 5b and Extended Data Fig. 7b). While this 20-mer peptide was sufficient to induce the mitoCPR, it did not impair mitochondrial protein import (Extended Data Fig. 7c). This enabled us to assess the consequences of mitoCPR activation in the context of functional protein import. Overexpression of MTS^Mge1(1-20)^-mCherry reduced cellular fitness, a phenotype rescued by *PDR3* deletion (Extended Data Fig. 7d-e). These findings suggest that inappropriate activation of the mitoCPR can negatively affect cellular fitness.

To test whether Mge1's presequence alone can restore mitoCPR signaling, we reintroduced *MTS^Mge1^-mCherry* expressed from the native *MGE1* promoter into a separation-of-function strain. Expression of MTS^Mge1^-mCherry fully rescued *PSD1*-induced *CIS1* upregulation (Fig. 5c and Extended Data Fig. 7f). Moreover, expression of only the first 20 amino acids (*MTS^Mge1(1-20)^-mCherry*) partially restored *CIS1* induction, confirming that this peptide contributes to mitoCPR activation (Fig. 5c and Extended Data Fig. 7f). Together, our data indicate that the Mge1 presequence is both necessary and sufficient for triggering the mitoCPR.

AlphaFold3 structural modeling suggested that the Mge1 N-terminus potentially interacts with Pdr3^42^. The N-terminal 17 residues of Mge1 were predicted to form a short helix that binds a negatively charged pocket within Pdr3's regulatory middle homology region (MHR)(Fig. 5d and Extended Data Fig. 7g)^42,43^. In contrast, models of previously reported nuclear-localized mitochondrial precursors had low confidence ipTM values (<0.6) (Extended Data Table 1)^18^. Arginine residues at positions 2 and 10 of MTS^Mge1^ were predicted to form ionic interactions with Pdr3, while phenylalanine 4 aligned with a cavity in Pdr3's pocket, which could potentially contribute to binding specificity (Fig. 5d). Complementary sequence analysis of all yeast mitochondrial presequences (residues 1–20) revealed that arginines commonly occupy positions 3 and 4, but are rarely found at position 2 (Extended Data Fig. 7h). This observation, which is consistent with previous studies^6,7^, suggests that R2 may be a unique feature of MTS^Mge1^. A similar enrichment was observed among the mitochondrial precursors previously detected in the nucleus, with R2, R10 and F4 uniquely present in Mge1's presequence (Extended Data Fig. 7i)^7,18^. Given that presequences often form amphipathic α-helices^6^, the positioning of arginine within the helix may be critical for aligning with the proper negatively charged regions on Pdr3.

To test the functional relevance of residues R2, R10, and F4, we introduced point mutations into full-length *MGE1* and expressed the resulting mutants from the native *MGE1* promoter. Upon *PSD1^OE^*, the precursor forms of Mge1^R2Q^, Mge1^R10Q^, and Mge1^F4A^ accumulated and localized to nuclei (Fig. 5e and Extended Data Fig. 7j-k). However, unlike wild-type Mge1, none of these mutants restored mitoCPR activation in the *MTS^Su9^-MGE1* separation-of-function background (Fig. 5f and Extended Data Fig. 7k). Since a reduced abundance of Mge1^F4A^ was observed in total cell lysates, presumably due to instability, the possibility that its protein level was insufficient to activate the mitoCPR cannot be excluded (Extended Data Fig. 7j). Together, these results suggest that the Mge1 presequence contains a distinctive combination of features that are required for mitoCPR signaling.

## Discussion

Our work reveal a dual function for the mitochondrial Hsp70 co-chaperone, Mge1: inside mitochondria, mature Mge1 mediates protein import and folding^1,32^, whereas inefficient import allows its precursor to enter the nucleus and transmit a stress signal. This signaling role depends on Mge1's MTS, which activates Pdr3 (Fig. 5g). Since many mitochondrial defects impair protein import, we propose a novel function for this targeting sequence as mitochondrial health indicator^2–4,9^.

Previous studies identified the ribosome-associated co-chaperone Zuo1 as an activator of Pdr1, but not Pdr3^44^. A short hydrophobic peptide at Zuo1's C-terminus is sufficient for Pdr1 activation^44^, suggesting that changes in ribosome state could lead to release and nuclear translocation of Zuo1. Thus, both Pdr1 and Pdr3 are regulated by small peptides within specific co-chaperones that translocate to the nucleus under distinct stresses: Mge1 reflects mitochondrial damage, while Zuo1 presumably acts as a protein translation sensor^44^. Although Pdr1 and Pdr3 have overlapping target genes^28,45^, it is unclear whether their resulting transcriptional profiles are identical upon activation by Zuo1 or Mge1.

Pdr1 is central to multidrug resistance, and both Pdr1 and Pdr3 regulate ABC transporter expression^28,45^. Direct binding of xenobiotics to Pdr1/Pdr3's xenobiotic-binding domains (XBDs) was suggested to induce transcription^46^. It is plausible that while xenobiotics activate Pdr1/Pdr3 via an extrinsic pathway, Zuo1 and Mge1 act through intrinsic pathways, which respond to intracellular damage. Our *in-silico* analysis predicts that Pdr3's MHR domain mediates interaction with Mge1. Future studies could provide experimental validation for this region and the XBD in Mge1 binding.

Our findings suggest that Mge1's nuclear localization depends on its presequence. However, a canonical NLS could not be identified within the presequence or other regions of Mge1^33^. As presequences are typically rich in positively charged residues, MTS^Mge1^ might contain a cryptic NLS that can be exposed upon helix formation or through interaction with factors such as Kap123. Yet, the mechanism that prevents such exposure and prevents the nuclear import of Mge1 under non-stress condition remains unclear.

General accumulation of unimported mitochondrial precursors in the cytosol signals mitochondrial dysfunction and contributes to retrograde signaling^4,9,15,19,47^. Specific stress sensors such as ATFS-1, DELE1, and PINK1 directly induce stress responses when protein import is impaired^4,12,14,48–51^. While this is the first report linking Mge1 to nuclear stress signaling, the mitochondrial activity of Mge1 and its human orthologs (GrpELs) is known to be stress-responsive. The co-chaperone activity of Mge1/GrpEL is regulated by oxidation, enabling a dynamic control of protein import under conditions of elevated reactive oxygen species (ROS)^52,53^. Since ROS mediate inter-organellar communication and cytosolic quality control of mitochondrial precursors, they may also modulate unimported Mge1 and GrpELs during stress^19,54,55^.

It remains unclear why Mge1 is the only mitochondrial precursor that activates the mitoCPR. Such activation requires a combination of features, including stability outside mitochondrial, nuclear import, and ability to interact with Pdr3, that together may be unique to Mge1. Mge1 import may be also particularly responsive to mitochondrial dysfunction. A similar mechanism was shown for ATFS-1, whose “weak” MTS confers high sensitivity to import defects^50,51^.

Interestingly, arginine at position 2, found in MTS^Mge1^, is thought to decrease protein import efficiency^6^. However, several computational tools predict this MTS to be relatively strong^37,56^. Thus, careful experimental comparison is needed to define the strength of MTS^Mge1^. If revealed to be “strong”, one interpretation is that the mitoCPR is reserved for severe mitochondrial import disruptions. Alternatively, Mge1 nuclear import may be regulated by additional factors that can override its otherwise “strong” mitochondrial targeting.

Unlike the high conservation of Mge1 from bacteria to vertebrates^32,57^, Pdr3 has no identifiable sequence homolog in mammals. Whether functional homologs of Pdr3 exist in mammalian cells and how they respond to mitochondrial stress remain unclear. If such homologs exist, their lack of sequence homology to Pdr3 predicts that they would bind to unique peptides different from MTS^Mge1^. This is consistent with the fact that like other mitochondrial presequences, MTS^Mge1^ is poorly conserved between orthologs^58^. Regardless of whether an equivalent Pdr3/Mge1-dependent pathway exists in mammals, the function of unimported MTSs in stress signaling may be conserved. Genetic variants were identified within human mitochondrial presequences, which until now, could only be considered to alter import efficiency^59^. Our work raises the possibility that some variants could also affect cells’ ability to respond to mitochondrial dysfunction. Revealing how human MTSs regulate transcriptional programs will be the scope of future studies.

## Methods

### Yeast strains and growth conditions

All strains are derivatives of W303 (HW505) and are listed in Supplementary Table 6. Cells were grown overnight in YPD (1% yeast extract, 2% bactopeptone, 2% glucose) at 30°C to saturation, then diluted in fresh YPD (OD_600_ = 0.1) and grown until they reached logarithmic phase. To induce the *GAL1-10* promoter, cells were grown overnight at 30°C in synthetic drop-out (SD) medium (0.17% Yeast nitrogen base without amino acids (BD, 291940), 0.2% amino acid mix) containing 2% raffinose. Cells were then diluted to OD=0.2 in the same medium and recovered for 1.5 hours. Galactose was added to the recovered cultures to a final concentration of 1%. Cultures were harvested during logarithmic phase (OD=0.8-1).

Acute mitochondrial protein import stress, induced by *PSD1* overexpression was achieved using 2μ plasmid containing *P_GAL1-10_-PSD1*, while empty vector was used as a non-stress control. The mitochondrial ribosomal subunit *MRPL16* was deleted to obtain *rho-* cells, which were inviable on medium lacking a fermentable carbon source^60^. The *rho-* genotypes were confirmed by measuring the mRNA levels of *COX1*, a mitochondrial DNA encoded gene (Supplementary Fig. 1c)^61^. In addition, the inability of the *rho-* strains to grow on medium lacking a fermentable carbon source following mating with an *MRPL16 rho0* strain was confirmed.

For split-GFP experiments, the nuclear protein Pus1 tagged with mCherry and the N-terminal fragments of GFP (GFP^1-10^) was expressed from a centromeric plasmid under control of the *NOP1* promoter^62^. Mge1 or Mss116, fused to the complementary C-terminal β-strand of GFP (GFP^11^) were expressed from a 2μ plasmid driven by the *GAL1–10* promoter.

Amino acids 1-44 of Mge1 were excluded from the *ΔMTS-MGE1* truncated mutants^63^. The *MTS^Ilv2^-MGE1* and the *MTS^Su9^-MGE1* constructs were generated by fusing either the N-terminal 38 amino acids of Ilv2^7^ or the N-terminal 69 amino acids of *Neurospora crassa* ATP-synthase subunit 9 (Su9) to residues 45-228 of Mge1. *MTS^Su9^* is a commonly used targeting signal of the *Neurospora crassa* ATP-synthase subunit 9^64^. To generate strains carrying these separation-of-function *MGE1* alleles, a diploid strain heterozygous for *MGE1* deletion (*MGE1/mge1*Δ) was first constructed. Tetrad dissection confirmed that the *mge1*Δ haploid cells were not viable (Extended Data Figure 5a). Single-copy integration plasmids carrying *P_MGE1_-MTS^Ilv2^-MGE1* or *P_MGE1_-MTS^Su9^-MGE1* were inserted at the *HIS3* locus of the *MGE1/mge1*Δ diploid. Viable *mge1*Δ haploid spores expressing the respective MTS-fusion constructs were obtained by tetrad dissection. The *MGE1-NES* construct was generated by fusing the nuclear export signal of mammalian protein kinase inhibitor α (PKIA) to the C-terminus of *MGE1*^65^. The *NLS-ΔMTS-MGE1* construct was generated by fusing the nuclear localization signal of SV40 to the N terminus of *ΔMTS-MGE1* (residues 45-228).

Proteasomal degradation was inhibited using 40uM of MG132 (Sigma-Aldrich, 474790). This treatment was done in a strain background deleted of *PDR5* and *ERG6* to increase the drug's potency. Cycloheximide was added to a final concentration of 0.5mg/mL following 1-hour incubation with MG132.

### Yeast strain construction

All yeast genetic manipulations, including gene deletions and tagging were obtained accordingly: a wild-type strain was transformed with PCR-amplified cassettes containing gene-specific homology arms and selectable markers. Transformants were selected on appropriate selective media, and positive colonies were genotyped to confirm correct integration at the intended genomic locus. To generate strains with multiple modified alleles, validated single-mutant strains were crossed, followed by sporulation and tetrad dissection using micromanipulation. Spores were screened via replica plating on selective media to isolate haploid strains with the desired combination of genetic modifications. Gene deletions verifications are presented in Supplementary Figure 1.

### Auxin inducible-degradation assay

The auxin-inducible degron (AID) system was used to degrade the precursor of Mge1. Since the proteasome is absent from mitochondria, this system affects only precursors on their way to the organelles^66,67^. *MGE1* and *UBX2* were C-terminally tagged with Myc-AID at their endogenous loci in yeast strains expressing *OsTIR1* integrated at the *HIS3* locus^68,69^. For auxin-induced degradation of Mge1, cells were first subjected to a one-hour 1mM indole-3-acetic acid (IAA; Sigma-Aldrich, I5148) treatment, followed by *PSD1 overexpression* for 4 hours in the presence of IAA. For Ubx2 degradation, log-phase cultures were treated with 1 mM IAA for 1 hour. Protein depletion was assessed by immunoblotting using anti-Myc antibody.

### Anchor-away assay

The anchor-away system was used to sequester Mge1. This system enables rapamycin-induced heterodimerization of human *FKBP12* with proteins fused to the FRB domain of human *mTOR*^70,71^. Anchor-away experiments were done using yeast strains harboring the *tor1-1* mutation and deleted of *FPR1* in the presence or absence of the plasma membrane anchor Pil1-mCherry-*FKBP*^70^. In addition, *MGE1* was C-terminally tagged with FRB domain of human *mTOR* at the endogenous locus^70^. Galactose was added to induce *PSD1* overexpression simultaneously with either vehicle (DMSO) or 50 nM rapamycin (Sigma-Aldrich, R0395) treatment, and cells were harvested after 4 hours.

### Yeast whole genome screening

The screen was conducted using strains isogenic to BY4741, BY4742 or BY4743. Query strain, carrying the *P_CIS1_-LEU2* reporter (*LEU2* gene under the promoter of the mitoCPR target, *CIS1*) and the hygromycin B resistance cassette at the *CIS1* locus, was mated with the MATa *P_GAL_-ORF-HA* collection. This collection contains 5,100 yeast ORFs that were constructed into a *URA3* 2μ plasmid under the inducible *GAL1-10* promoter^72^. Heterozygous diploids were selected on a synthetic medium (0.17% Yeast nitrogen base without amino acids and Ammonium Sulfate (BD, 233520), 0.1% Glutamic acid, 2% glucose, 0.2% amino acid mix) lacking uracil and supplemented with hygromycin B (100 μg/ml, Roche, 10843555001). Since ammonium sulfate impedes the function of hygromycin B, synthetic medium containing this antibiotic was prepared with monosodium glutamic acid (MSG, Sigma, G1626) as a nitrogen source. A resulting array of heterozygous diploids was then transferred to galactose containing plates to induce overexpression (synthetic complete (SC) medium supplemented with 2% raffinose and 1% galactose). Expression of the *P_CIS1_-LEU2* reporter was assessed by measuring colony growth on galactose medium lacking leucin. The same array was grown on galactose medium in the presence of leucin to control for the effect of *GAL1*-induced overexpression on growth. The plates were scanned using a flatbed scanner (B11B231201, EPSON) at a resolution of 300 dpi.

### Screen data analysis

The “BALONY” automated computer-based scoring system was used to analyze digital images of colonies to generate an estimate of the relative growth rate based on pixel density^73^. To eliminate dead and sick colonies, strains with colony size smaller than 20 pixels on leucin-containing medium were excluded. To account for the variability of the average size from plate to plate, the obtained colony size values from each plate were normalized to a grand average of all the plates from the same treatment group. After normalization, mean Z-scores were calculated for each gene with colony size grown on the medium lacking leucin and the value obtained on leucin-containing medium. Finally, the mean Z score difference between the two-treatment group were calculated. The hit list was determined by sorting the mean Z-score difference from high to low among the strains that have a q-value lower than 0.05^74^.

### Immunoblot analysis

For immunoblot analyses, approximately 1.2 *OD_600_* units of cells were harvested and treated with 5% trichloroacetic acid overnight at 4°C. The TCA was removed by washing with 100% acetone, and the resulting cell pellet was air-dried. The cell pellet was lysed by beads beating in 100 μl of lysis 1xTE (50mM Tris–HCl at pH 7.5, 1 mM EDTA, 2.75 mM DTT). Samples were boiled in 1X SDS sample buffer for 5 min.

Samples were separated by SDS-PAGE containing stain-free dye (TGX Stain-Free FastCast acrylamide kits, BioRad), transferred onto nitrocellulose membranes, and subsequently probed with the following primary antibodies (all at 1:1000 dilution): anti-GFP (Sigma-Aldrich, mouse 11814460001), anti-V5 (Abcam, mouse AB27671), anti-FLAG (Sigma, mouse F1804), anti-mCherry(Abcam, rabbit AB213511), anti-HA (Biolegend, 901513), anti-Pgk1 (Abcam, ab113687). For detection of endogenous Mge1, Mge1 antisera was used (1:200 dilution; gifts from Dr. Nikolaus Pfanner, University of Freiburg). Secondary detection was performed using one of two antibody types: (1) DyLight™ 800 4X PEG-conjugated anti-mouse IgG (H+L) (1:15,000 dilution; NEB 5257) or (2) HRP-conjugated donkey anti-rabbit IgG (1:10,000 dilution; Cytiva NA9341ML). Fluorescent signals and total protein (via stain-free imaging) were detected using the ChemiDoc Imaging System (Bio-Rad). Band intensities were quantified using Image Lab software (Bio-Rad Laboratories) and normalized to total protein staining. All gels and blots are presented in Supplementary Figure 2.

### Mitochondrial isolation and protease protection assay

Cells were grown to logarithmic phase, collected by centrifugation and washed once with water. Wet weight of the pellet was recorded. Cells were then resuspended in DTT buffer (0.1 M Tris pH 9.4, 10 mM DTT) and incubated for 20 min at 30°C. Cell walls were disturbed by incubation in zymolyase buffer (1.2 M sorbitol, 20 mM K_2_HPO_4_ pH 7.4) containing 5 mg of Zymolyase 20T (amsbio, 120491-1) per 1 g cell pellet for 1 hour at 30°C. Dounce homogenization was used to lyse the cells in 0.6 Msorbitol, 10 mM Tris pH 7.4, 1mM EDTA, fatty acid free 0.2% BSA and 1 mM PMSF. Mitochondria were then isolated by differential centrifugation as described previously and resuspended in SEM buffer (0.25 M sucrose, 10 mM MOPS KOH pH 7.2 and 1 mM EDTA)^75^. Proteinase K was added to a final concentration of 25 mg/ml or 50 mg/ml for 5 min at 37°C and the reaction was stopped by the addition of 4 mM PMSF for 15 min on ice.

### *In organello* protein import assay

The coupled Transcription/Translation system (T7 Quick for PCR DNA, Promega) was used to express Mdh1, Zim17, Mge1, and Mge1-FLAG from a PCR template using primers listed in Supplementary Table 6. A *Saccharomyces cerevisiae* codon optimized templet was used for the amplification of *MGE1*. Precursor proteins were synthesized in reticulocyte lysate in the presence of 35S-L-methionine and 35S-L-cysteine (EasyTag EXPRESS^35^S Protein Labeling Mix, Revvity). Import into isolated mitochondria was performed in import buffer (3% (w/v) BSA, 250 mM sucrose, 80 mM KCl, 5 mM methionine, 5 mM MgCl2, 2 mM K_2_HPO_4_, 10 mM MOPS-KOH, pH 7.2, 4 mM NADH, 2 mM ATP, 5 mM creatine phosphate, 0.1 mg/ml creatine kinase) at 25 °C. The import reaction was stopped on ice or by addition of AVO (8 μM antimycin A, 20 μM oligomycin, 1 μM valinomycin). To dissipate Δψ, AVO was added to the mitochondria prior to the incubation with the proteins. Samples were treated with 25 μg/ml proteinase K for 15 min on ice, following by treatment with 2 mM PMSF for 5 min on ice. Mitochondrial were washed twice with SEM buffer and analyzed by electrophoresis on SDS-polyacrylamide gel electrophoresis. Dried gels were exposed to a storage phosphor screen (Cytiva, 28956475), which was then scanned using a Typhoon Trio scanner (Amersham).

### Membrane potential measurement

Mitochondria were resuspended in ice-cold import Buffer (3% (w/v) BSA, 250 mM sucrose, 80 mM KCl, 5 mM methionine, 5 mM MgCl2, 2 mM K_2_HPO_4_, 10 mM MOPS-KOH, pH 7.2) to a final protein concentration of 0.25 mg/mL. For membrane potential measurement, 100 uL of import buffer containing 1 μM of DiSC3(5) was added to a black-wall clear-bottom 96 well plate (Cellvis P96-1.5H-N), and fluorescence was monitored with a Varioskan FLASH plate reader and SkanIt Software (Thermo Scientific). After linear shaking for 5 seconds, fluorescence was measured for 1.5 min at a 5-second interval with excitation at 622 nm and emission at 670 nm. Resuspended mitochondria were then added to reach a final concentration of 0.05 mg/mL. The plate was shaken linearly for 5 seconds, and fluorescence was measured for 3 min with the same setting. Lastly, 2 μM of valinomycin was added to dissipate the membrane potential. After 5 seconds of linear shaking, fluorescence was measured for 1.5 min with the same setting. For each replicate, fluorescence intensity was normalized to the maximum intensity observed within that individual trace. Membrane potential was quantified by averaging the normalized fluorescence signal over the 3-min measurement window following mitochondria addition.

### Nuclei isolation

*PSD1*-overexpressing or *rho-* cells were grown as described above. For input samples, approximately 1.5 *OD_600_* units of cells were harvested and treated with TCA as described above (immunoblot analysis section). The following nuclei isolation procedure were modified from previous studies^18,76^. In brief, 80 *OD_600_* units of cells were collected and spheroplasted as described in the mitochondrial isolation section. Spheroplasts were then placed on ice, and resuspended in 10 ml of freshly prepared, ice-cold resuspension buffer (1.2 M sorbitol, 20 mM piperazine-*N*,*N’*-bis(2-ethanesulfonic acid) (PIPES, pH 6.8) and 1 mM MgCl_2_). Cells were pelleted at 1,300 ×*g* for 5 min at 4°C and resuspended in 5 mL of freshly prepared, ice-cold nucleus isolation buffer (0.25 M sucrose, 60 mM KCl, 14 mM NaCl, 5 mM MgCl_2_, 1 mM CaCl_2_, 15 mM morpholineethanesulfonic acid (MES hydrate, pH 6.6), 1 mM phenylmethylsulfonyl fluoride (PMSF), 0.8% Triton X-100). The re-suspended pellet was incubated on ice for 30 minutes, then pelleted at 1,900×*g* for 10 min at 4°C. A fraction of the supernatant (1%) was collected and incubated with SDS sample buffer at 95 for 5 minutes. The nuclei containing pellet was resuspended in 1 mL of nuclear IP buffer (50 mM Tris-HCl pH 7.5, 150 mM NaCl, 1 mM EDTA, 10% glycerol, 1% IGEPAL, 100 mM PMSF (added fresh), and protease inhibitor (cOmplete, Roche, 4693132001)). Intact nuclei were incubated with magnetic agarose beads at 4 for 2 hours for non-specific immobilizaion (Chromotek, bmab), as described previously^18^. Immobilized nuclei were then washed 4 times on ice with nuclear IP buffer by gentle pipetting. Nuclear-enriched extracts were eluted by incubating the beads in 2X SDS sample buffer at 95 for 5 minutes.

### Co-immunoprecipitation

Approximately 40 OD_600_ units of exponentially growing cells were pelleted and resuspended in 200 μl of lysis buffer (50 mM Tris-HCl pH 7.5, 250 mM NaCl, 1 mM EDTA, 5% IGEPAL, and protease inhibitor cocktail (cOmplete, Roche, 4693132001). Cells were mechanically lysed at 4°C with silica beads using a FastPrep-24 homogenizer (MP Biomedicals) for 5 cycles (6.5 m/s for 45 seconds, with 5-minute intervals between cycles). Cell lysate was cleared by centrifugation at 20,000 × g for 10 min at 4°C. Protein concentration was determined using a Bradford assay and 2 mg of total protein from each sample was adjusted to 1.5 mL with lysis buffer. Co-immunoprecipitation were done using either protein G magnetic beads (Fig. 2f and Extended Data Fig.4c) or V5-trap beads (Fig. 2i, 3d and 4d and Extended Data Fig.4b and 6f) as follows: 1. Protein G magnetic beads (Dynabeads, Invitrogen) were incubated with primary antibody for 30 min at room temperature, then mixed with lysates for 2.5 h at 4 °C. Beads were washed 5 times with cold wash buffer (1× PBS with 0.1% Tween-20), and bound proteins were eluted by boiling in 1× SDS sample buffer at 95 °C for 5 min. 2. V5-trap magnetic agarose beads (Chromotek, V5TMA) were pre-equilibrated according to the manufacturer's instructions and incubated with lysates for 1–2 hours at 4 °C. Beads were then washed three times with washing buffer (10 mM Tris-HCl pH 7.5, 150 mM NaCl, 0.05 % IGEPAL, 0.5 mM EDTA). For SDS-PAGE analysis, bound proteins were eluted with 1× SDS sample buffer and boiled at 95 °C for 5 minutes. For mass spectrometry, pulled-down proteins were eluted in simplified sample buffer (50 mM Tris-HCl pH 6.8 with 2% SDS) by incubating on ice for 2 minutes with gentle tapping.

### Sample processing for Mass Spectrometry (MS) analysis

Two independent co-IP experiments were performed in 3 biological replicates. For experiment 1, whole cell lysates (WCL) and co-IP eluates were analyzed: (1) *PSD1* overexpression, untagged Pdr3; (2) *PSD1* overexpression, V5-Pdr3; (3) control, V5-Pdr3. For experiment 2, only co-IP were analyzed for 4 conditions: (1) *PSD1* overexpression, untagged Pdr3, wild-type Mge1; (2) *PSD1* overexpression, V5-Pdr3, wild-type Mge1; (3) control, V5-Pdr3, wild-type Mge1; (4): *PSD1* overexpression, V5-Pdr3, MTS^Ilv2^Mge1 (One replicate from samples 2 and 3 were compromised during processing and removed). WCL (5μg) and the co-IP eluates underwent an SP3 trypsin digestion protocol^77^. Briefly, tris(2-carboxyethyl)phosphine) (TCEP) was added to a final concentration of 3mM and incubated for 20min, followed by the addition of 2-chloroacetamide to a final concentration of 55mM for 30min. All incubations were performed at 25°C on a thermomixer set at 900rpm, unless specified. Thereafter, Sera-Mag SpeedBeads™ Carboxyl Magnetic Beads (Thermofisher, 88817) were added in each sample at a 1:10 protein-to-bead ratio (1μg of proteins was guesstimated per co-IP eluate). 100% ethanol was then added at a 50:50 ratio and incubated for 10min. The supernatants were removed by placing the plate on a 96-well magnetic platform (Permagen Labware, S500) for 2min. The protein-bound beads were washed three times with 200μL of 80% ethanol. 200μL (or 100μL for WCL) 50mM HEPES (pH 8.0) with 1:25 trypsin-to-protein ratio (Promega V5113) was added to beads and incubated overnight (16-18h) at 37°C, 900rpm. For stage tipping, the digested samples were placed on a 96-well magnetic platform for 2min; the supernatants were then transferred into a 96-well PCR plate (Greiner, 652270) and acidified with trifluoroacetic acid (TFA) to a final concentration of 1%. The samples were then purified using AssayMAP C18 cartridge tips (Agilent, 5190-6532) and Stop-and-Go Extraction tipped (StAGE-tipping)^78^ based on the manufacturer's built-in protocol on a Bravo Automated Liquid Handling Platform and eluted with 100μL of 40% acetonitrile (ACN) and 0.1% TFA. The samples were dried in speedVac then reconstituted with 20μL of 0.5% ACN and 0.1% formic acid before MS analysis.

Processed peptides from WCL (100ng) and an estimated 30ng from co-IP samples were analyzed using DIA acquisition mode on an Orbitrap Exploris 480 coupled with Thermo EASY-nLC, an Aurora Series analytical column (25cm x 75μm 1.6μm C18; Ion Opticks) heated to 40°C with an integrated column oven (PRSO-V2, Sonation) and a Nanospray FlexTM ion source operated at 1900 V spray voltage and ion transfer tube was heated to 290°C. MS1 scans were acquired at a resolution of 60,000 over an m/z range of 380–985, with a maximum injection time of 25ms. MS2 scans were acquired at a resolution of 15,000 using 60 DIA windows covering a precursor range of 380–980m/z. Each window had a width of 10 m/z with a 1 m/z overlap and a maximum injection time of 40ms. MS2 scans were collected over an m/z range of 145–1450. The cycle time was set to 3 seconds and one MS1 scan was acquired after every 30 MS2 scans. The mass-to-charge ratio was calibrated based on three selected ions from PierceTM FlexMixTM Calibration Solution and the mass accuracy was typically within 2ppm and is not allowed to exceed 4ppm. The peptides were separated using an 87-min gradient with a 250nl/min flow rate with 1min of 2% Buffer B, followed by 45min gradient to 20%, 15min gradient to 32%, 5min gradient to 50%, 5min gradient to 95% Buffer B, and finished with 8min wash at 95% Buffer B, 2min gradient to 3% and 6min run at 3% Buffer B. The predicted spectral library was first generated in DIA-NN (version 2.0.2) using proteome database of the Saccharomyces cerevisiae strain S288C downloaded from Uniport (S288C-2023.06.26.fasta)^79^. Precursor ions in the library are allowed to contain up to 1 missed cleavage, N-term Methionine excision, and carbamidomethylation on cystine residues and set to have a peptide length of 7-30 amino acids, a precursor charge range of 1-4, and a precursor m/z range of 300-1800. All WCL or co-IP files were first analyzed as unrelated runs to determine optimal mass accuracy, MS1 accuracy, and scan window and batch analysis of all the samples was then performed with the optimized parameters. One replicate of Sample 1 in Experiment 1 was discarded due to apparent sample contamination. To determine candidate interactors of Pdr3, quantifications of peptide fragments was used with Significance Analysis of INTeractome (SAINTq) by comparing sample 1 (control) and sample 2 (bait) of the first co-IP experiment. Briefly, fragment IDs and quantifications generated by DIA-NN were first filtered and formatted for SAINTq analysis using in-house R scripts^80^. Only fragments with peptide Q value below 0.01, protein Q value below 0.05, peptide library Q value (batch) below 0.01, and library protein Q value (batch) below 0.01 were kept. The log_2_ of the maxLGQ intensities of the candidate Pdr3 interactors (BFDR < 0.0025, n=194) were then compared between stress and no stress conditions (samples 2 and 3) using an unpaired t-test using a Holm-Sidak p value correction in PRISM (version 10.3.1). For WCL, protein levels were compared between stress and no stress conditions (samples 2 and 3) for any proteins quantified in 3 replicates of one of the two conditions (n=4247) and missing values were imputed with random values from the bottom 1% data points in the dataset using imputeLCMD package in an in-house R script. Log_2_ of the median-normalized maxLGQ intensities were then compared using an unpaired t-test using a Benjamini and Hochberg FDR correction in PRISM. For the second co-IP, log_2_ of the maxLGQ intensities of Mge1 were compared between samples 2-4 using a one-way ANOVA.

### RNA isolation and quantitative PCR

Total RNA was isolated using the RNAspin Mini kit (Cytiva). RNA (750 ng) was used to generate cDNA using the Iscript Reverse Transcription Supermix (Bio-Rad). Quantitative PCR was performed using the PerfeCTa SYBR Green FastMix (Quantabio) or Supergreen qPCR Mastermix (Wisent Bioproducts, 800-435-QL) and amplified using QuantStudio 3 Real-Time PCR System (ThermoFisher Scientific). Primers sequences are listed in Supplementary Table 6. Raw Ct values were analyzed manually using the 2^−ΔΔCt^ method^81^. Briefly, the mean Ct of technical replicates were calculated for both the target gene and the reference gene (*ACT1*). ΔCt was obtained by subtracting the reference gene Ct from the target gene Ct. ΔΔCt was calculated by comparing the ΔCt of experimental samples to that of the control sample (e.g., ΔCt _*PSD1*-overexpression_ - ΔCt _empty vector_). Fold changes in gene expression were determined as 2^−ΔΔCt^, representing the normalized expression of the target gene in experimental samples relative to the control. Statistical analysis was performed using GraphPad Prism 9. Significant differences between pairs were detected using one-way ANOVA with multiple comparisons using Dunnett's test, unless otherwise specified in the figure legends.

### Gene expression analysis

For RNA expression analysis, Psd1, MTS^Mge1^, and MTS^Mss116^ were overexpressed for 4 hours. Total yeast RNA was isolated using the RNAspin Mini kit (Cytiva). Sample quality control was performed using the Agilent 2100 Bioanalyzer or the Agilent 4200 TapeStation. Qualifying samples were then prepped following the standard protocol for the Illumina Stranded mRNA prep (Illumina). Sequencing was performed on the Illumina NextSeq2000 with Paired End 59bp × 59bp reads. Sequencing data was demultiplexed using Illumina's BCL Convert. De-multiplexed read sequences were then aligned to the Saccharomyces cerevisiae (R64-1-1) reference sequence using DRAGEN RNA app on Basespace Sequence Hub. Gene-level read counts were quantified using the featureCounts function from the Rsubread package (version 2.23.2) in R (version 4.4.1). Differential expression analysis was conducted using DESeq2 (v1.48.1) with default parameters. RNA sequencing data can be accessed via the following link: https://www.ncbi.nlm.nih.gov/geo/query/acc.cgi?acc=GSE303345

### Fluorescent microscopy

Cells were grown overnight at 30°C in SD medium with 2% raffinose. Overnight cultures were diluted to OD=0.2 in the same medium and grown for 3 hours following addition of galactose (final concentration of 2%). Cultures were then grown for 4 hours to induce the *GAL1-10* promoter. Live-cell fluorescence images were acquired using a Leica THUNDER 3D cell imager (Leica Microsystems) with wide-field imaging. Images were taken with a HCX PLAPO 100X objective (Leica Microsystems) and a Leica K8 camera. Image analysis was performed using ImageJ software (1.54j).

### Chromatin immunoprecipitation

Harvested cells were crosslinked with formaldehyde for 20 min at room temperature and the reaction was quenched by the addition of 100 mM glycine. Cells were washed with FA lysis buffer (50 mM HEPES pH 7.5, 150 mM sodium chloride, 1 mM EDTA pH 7.6, 1% Triton X-100, 0.1% sodium deoxycholate), snap frozen and stored at −80 °C. Cells were lysed in cold FA lysis buffer with cOmplete Mini Protease Inhibitor Cocktail (Roche). Samples were homogenized with zirconia beads (BioSpec) using Mini-Beadbeater-96 (BioSpec). The chromatin fraction was subjected to shearing by 9 cycles of 30 seconds sonication followed by 30 seconds break using a Bioruptor Plus (Diagenode). Tagged proteins were immunoprecipitated using anti-V5 agarose beads (Sigma-Aldrich) or anti-FLAG M2 magnetic beads (Sigma-Aldrich) at room temperature for 2 h with rotation. Subsequently, the beads were washed with FA lysis buffer with 400 mM sodium chloride and a lithium chloride/detergent buffer (10 mM Tris pH 8, 250 mM lithium chloride, 0.5% NP-40, 0.5% sodium deoxycholate, 1 mM EDTA). Samples were reverse crosslinked overnight in TE buffer with 1% SDS at 65 °C, 500 rpm and treated with 80 μg/mL proteinase K (Thermo Scientific) at 37 °C for 2 h. Purified DNA fragments were quantified by quantitative PCR using PowerUp SYBR Green Master Mix (Applied Biosystems, A25742) and an Applied Biosystems 7500 Fast Real-Time PCR System (Thermo Fisher Scientific). ChIP signals were corrected by an input, and subsequently were normalized over the silent mating type cassette *HMR*. Primer sequences are listed in Supplementary Table 6.

### Growth Curve Analysis

Cells were grown to logarithmic phase and 0.02 *OD_600_* units were transferred to 96-well plate wells (Millipore Sigma, M9436). Cell growth was measured throughout 48 hours at 30°C with orbital shaking using BioTek Epoch Microplate Spectrophotometer for 48 hours. Data with appropriate time points were fitted to a linear equation, with exponential growth constants derived from the slopes of the linear equation using *growthcurver* package in R (version 4.4.1)^82^. Statistical analyses were performed using GraphPad Prism 9, with significant differences between groups determined by one-way ANOVA or Student's t-test, as specified in the figure legends.

### Evolutionary analysis

Genomes of different *Saccharomyces* were downloaded from NCBI for analysis of *MGE1* sequence evolution. For species with gene annotations, we blasted the *S. cerevisiae MGE1* (blastn v2.14.0+) to the transcriptome to identify the homolog which we then blasted back to the *S. cerevisiae* (R64) CDS sequences to ensure reciprocal best hit. For species without annotations, we located *MGE1*'s position in the genome by blasting with S. cerevisiae *MGE1*, followed by manual curation of the ORF. We used mafft (v7.505) --localpair --maxiterate 1000 to align the amino acid sequences^83^. Conservation score calculations and alignment visualization from the alignments were carried out with Jalview (v2.11.3.2)^84^.

### MTS sequences analysis

To assess amino acid composition trends within mitochondrial targeting sequences (MTSs), an enrichment analysis was performed across the first 20 amino acids of the MTSs from two protein sets: (1) all annotated yeast mitochondrial proteins^7^ and (2) a subset of nuclear-localized mitochondrial proteins previously reported in the literature^18^. These raw frequencies were then normalized by the genome-wide amino acid usage frequencies following previously described method^85^. Enrichment scores were visualized as heatmaps, with each cell representing the log2-transformed enrichment of a specific amino acid at a given position.

### Statistics and reproducibility

Sample size and replication number (*n*) for each experiment is specified in the figure legends. Statistical analyses were performed using GraphPad Prism 9 and Microsoft Excel. Details of statistical tests and significance thresholds used for data analysis are provided in the Methods or figure legends.

## Extended Data

**Extended Table 1:** ipTM scores for predicted interactions between Pdr3 and the N-terminal 50 residues of nuclear-localized mitochondrial proteins. The list of mitochondrial protein precursors was obtained from Shakya *et al*., 2021. Only N-terminal MTS-containing proteins are included.

Target	pTM	ipTM
Mge1	0.84	0.61
Rex2	0.84	0.36
Abf2	0.84	0.37
Fmp16	0.84	0.45
Kgd2	0.84	0.35
Mdh1	0.81	0.37
Sdh2	0.83	0.31
Ilv2	0.83	0.34
Lsc2	0.83	0.38
Pdb1	0.84	0.34
Yml6	0.83	0.3
Ilv6	0.83	0.34
Hem15	0.84	0.54
Dld2	0.83	0.31
Lpd1	0.81	0.28

## Supplementary Material

Supplementary Figure 1

Supplementary Figure 2

Supplementary Table 1

Supplementary Table 2

Supplementary Table 3

Supplementary Table 4

Supplementary Table 5

Supplementary Table 6

## Data Availability

All data supporting the findings of this study are available within the paper and its Supplementary Information. Supplementary Figure 1 contains verification of yeast strains described in the Methods section (“Yeast strain construction”). Full version of all gels and blots is provided in Supplementary Figure 2. Source data for the overexpression screen are provided in Supplementary Table 1. Mass spectrometry (associated with Fig. 3d and Extended Data Fig. 6f) have been deposited to the ProteomeXchange Consortium via the PRIDE^86^ with the dataset identifier PXD066688 (publicly available through: https://www.ebi.ac.uk/pride/archive/projects/PXD066688). Source data for the mass spectrometry are also provided in Supplementary Table 2. RNA sequencing data (associated with Fig. 4b and Extended Data Fig. 6c) can be accessed via the following link: https://www.ncbi.nlm.nih.gov/geo/query/acc.cgi?acc=GSE303345. Source data for these RNA-seq analysis are also provided in Supplementary Table 3-5. Source data behind all graphs are provided. There are no restrictions on data availability.
