# Supplementary material for: A direct role for a mitochondrial targeting sequence in signaling stress
Source: Nature. Author manuscript; Available in PMC 2026 Feb 4. (PMC7618714; doi:10.1038/s41586-025-09834-x)
Supplement: Supplementary Figure 1 [file EMS212253-supplement-Supplementary_Figure_1.pdf]

nature portfolio

https://doi.org/10.1038/s41586-025-09834-x

Supplementary information

A direct role for a mitochondrial targeting sequence in signalling stress

In the format provided by the authors and unedited

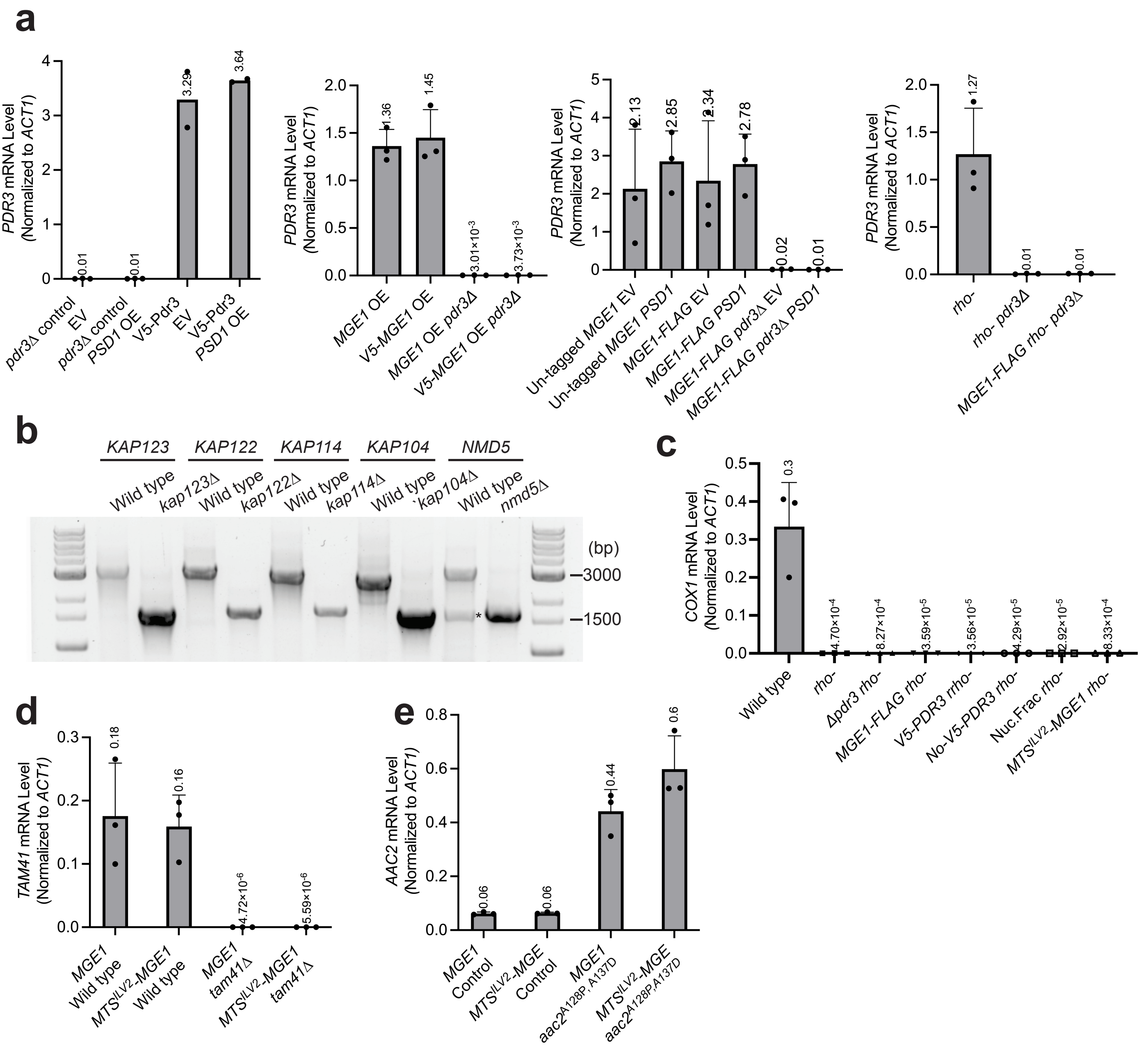

Supplementary Fig. 1| Verification of yeast strains. (**a**) QPCR analyses of *PDR3* mRNA levels in the *PDR3* deletion strains used in this study (Fig.1b, Fig.1d, Fig. 2g, Extended Data Fig. 1c, Extended Data Fig. 1h, Extended Data Fig. 4d and Extended Data Fig. 4f). Absolute mRNA levels of *PDR3* and the reference gene *ACT1* were calculated based on a standard curve. At least 2 biological replicates were tested for strains used in Fig. 1b and Extended Data Fig. 1c. For other strains, n = 3 biological replicates. (**b**) DNA was extracted from wild-type yeast and from strains carrying deletions of importin-β genes used in this study (n= 1 biological replicate; Fig 2d-e and Extended Data Fig. 3g). Open reading frames (ORFs) of the various importin-β genes were PCR-amplified and analyzed by gel electrophoresis (primer sequences are details in Supplementary Table 8). The size of the amplified product distinguished between the wild-type gene ORF and the *KanMX* deletion cassette, which was integrated by homologous recombination to replace the corresponding gene (*KanMX* deletion cassette-1560 bp, *KAP123*-3342 bp, *KAP122*-3246 bp, *KAP114*-3015 bp, *KAP104*-2757 bp, NMD5-3144 bp). The asterisk indicates a non-specific PCR product. (**c**) The mRNA levels of *COX1*, a mitochondrial DNA-encoded gene, were analyzed by qPCR to verify the genotype of the *rho-* strains used in this study (Fig. 2h, 2i, Fig. 4g, Extended Data Fig. 3e-k, Extended Data Fig. 6j). Absolute mRNA levels of *COX1* and the reference gene *ACT1* were calculated based on a standard curve. n=3 biological replicates. (**d**) Same as (a) showing *TAM41* mRNA level relative to *ACT1* in wild type cells and *tam41*Δ cells (Fig. 4h and Extended Data Fig. 6j). (**e**) *AAC2* mRNA level in cells expressing *aac2*$^{A128P,\ A137D}$ and control cells. Absolute mRNA level of *AAC2* and reference gene *ACT1* were calculated based on a standard curve. n=3 biological replicates. (Fig. 4i and Extended Data Fig. 6j). (**a, c-e**) Data are shown as mean +/− SD.
