# Supplementary material for: A direct role for a mitochondrial targeting sequence in signaling stress
Source: Nature. Author manuscript; Available in PMC 2026 Feb 4. (PMC7618714; doi:10.1038/s41586-025-09834-x)

Figure 2a

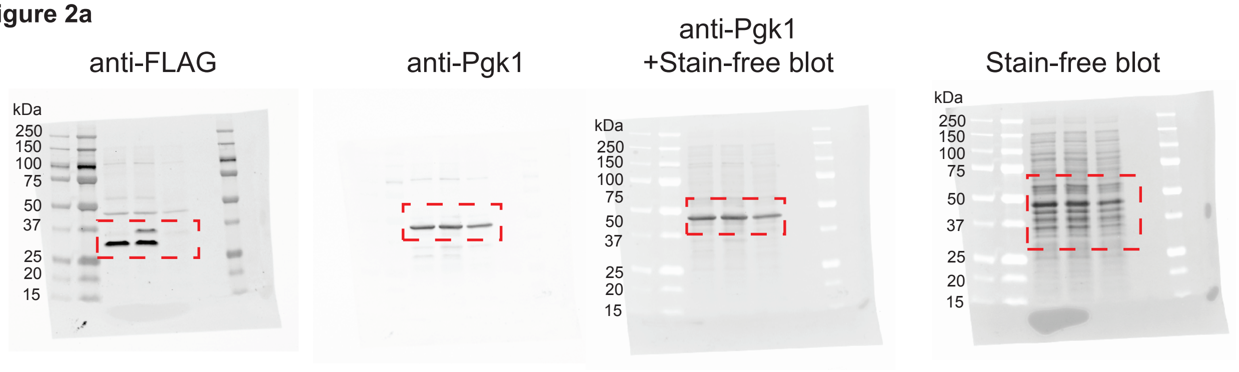

Figure 2b

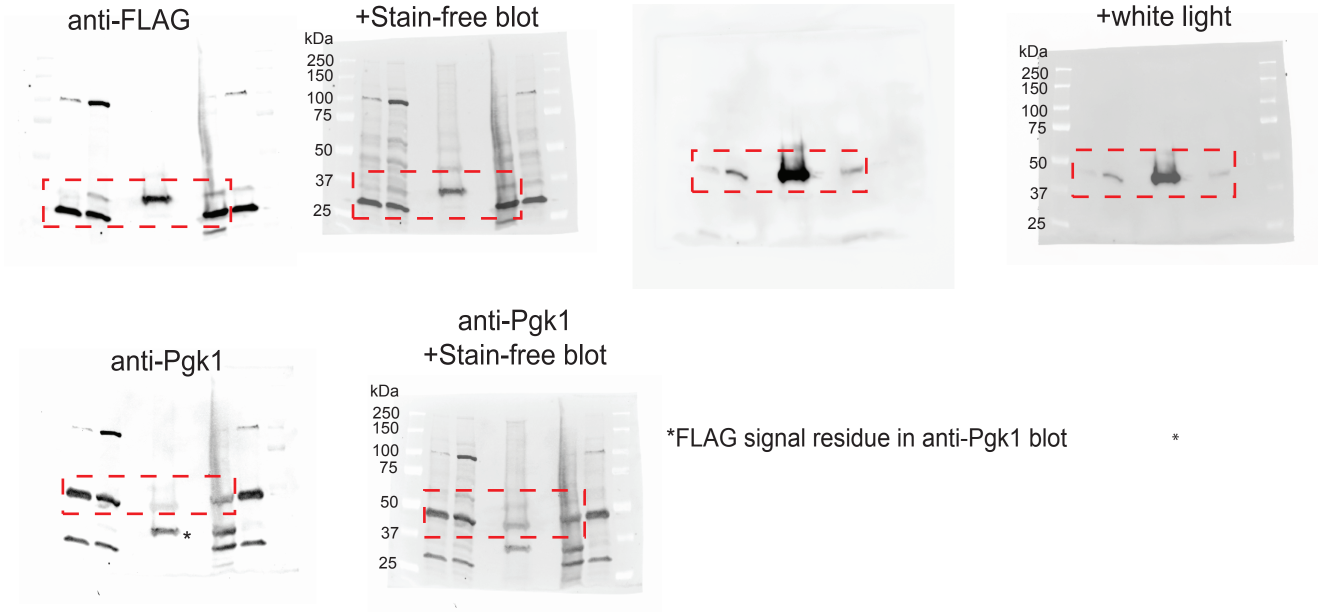

*FLAG signal residue in anti-Pgk1 blot

Figure 2c

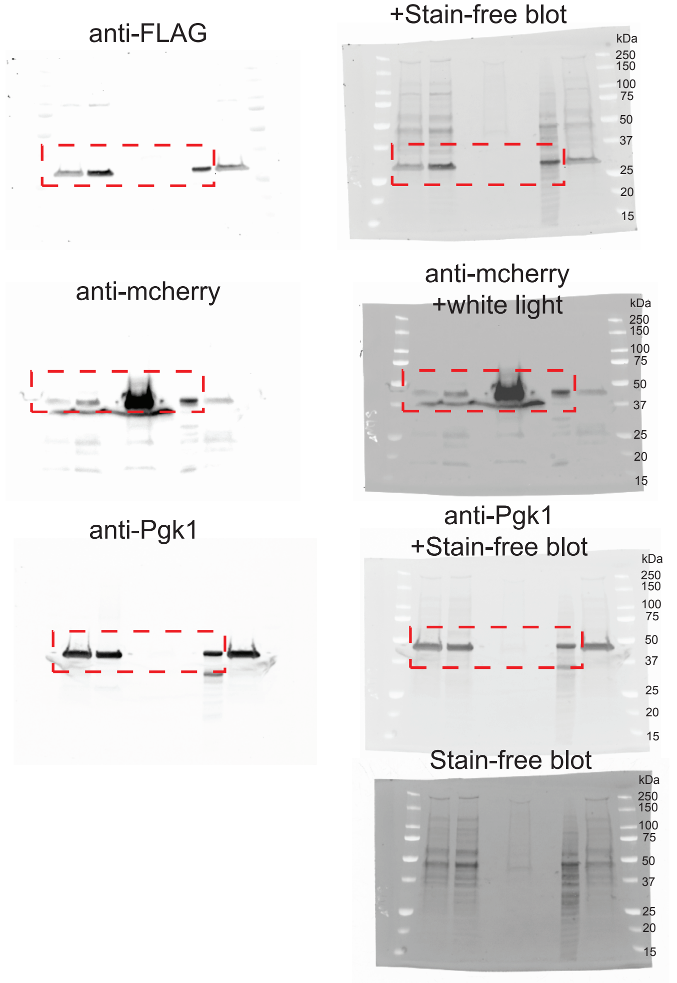

Figure 2e

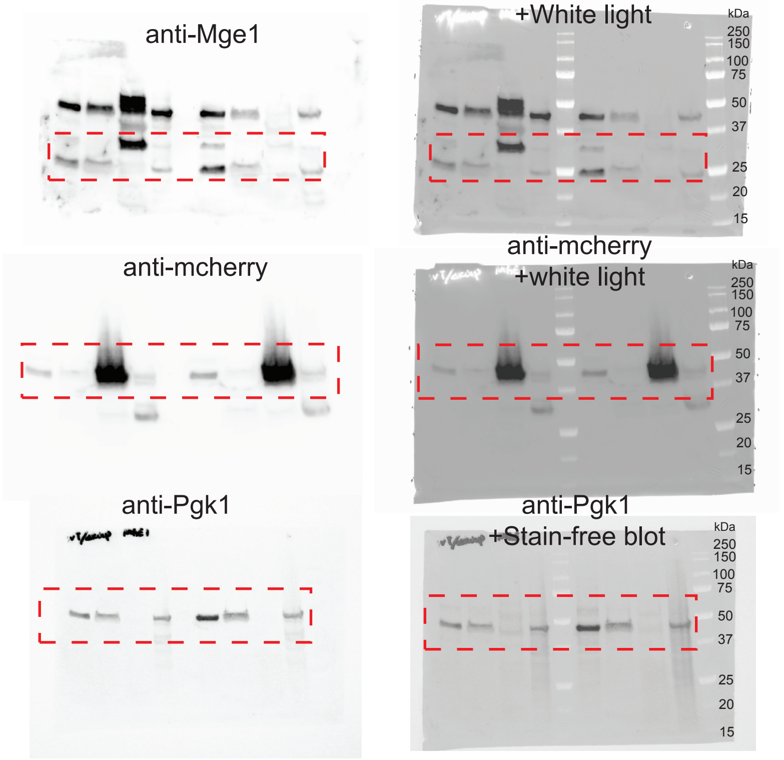

Figure 2f

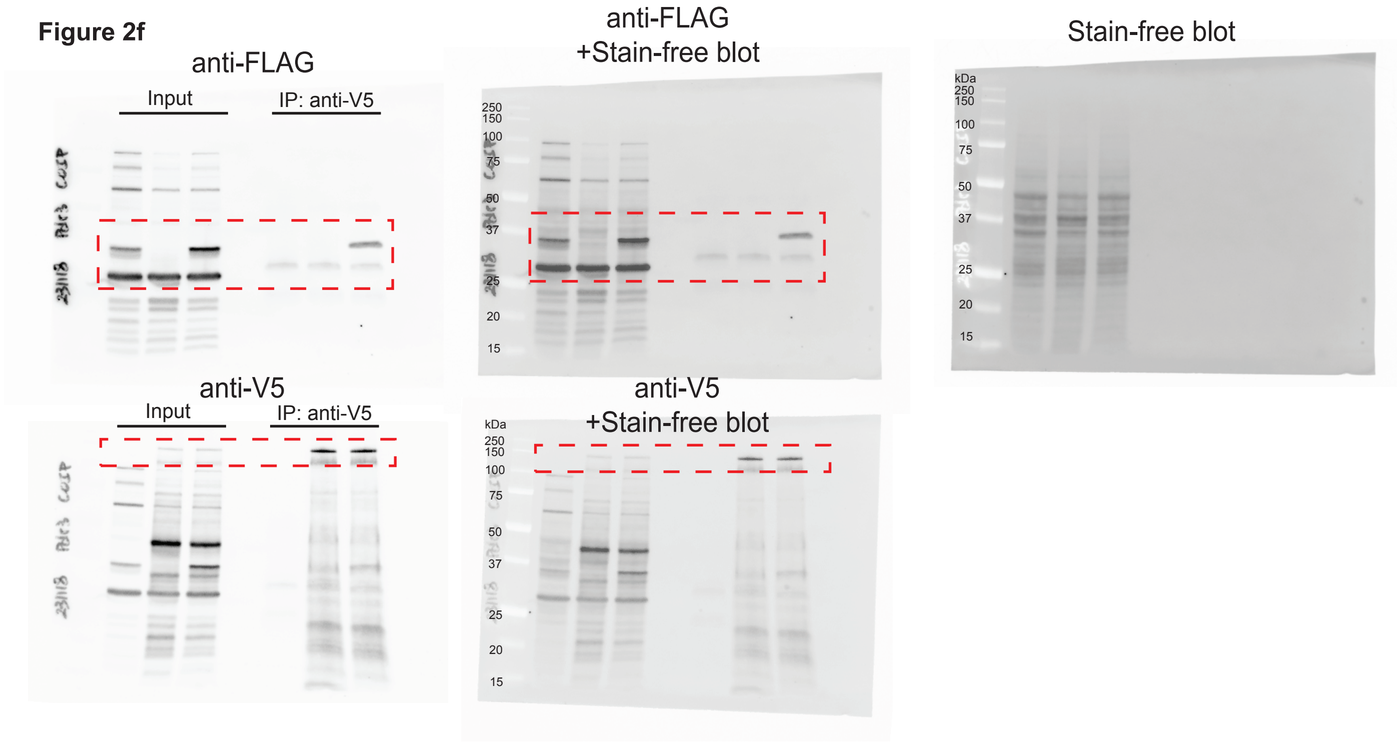

Figure 2h

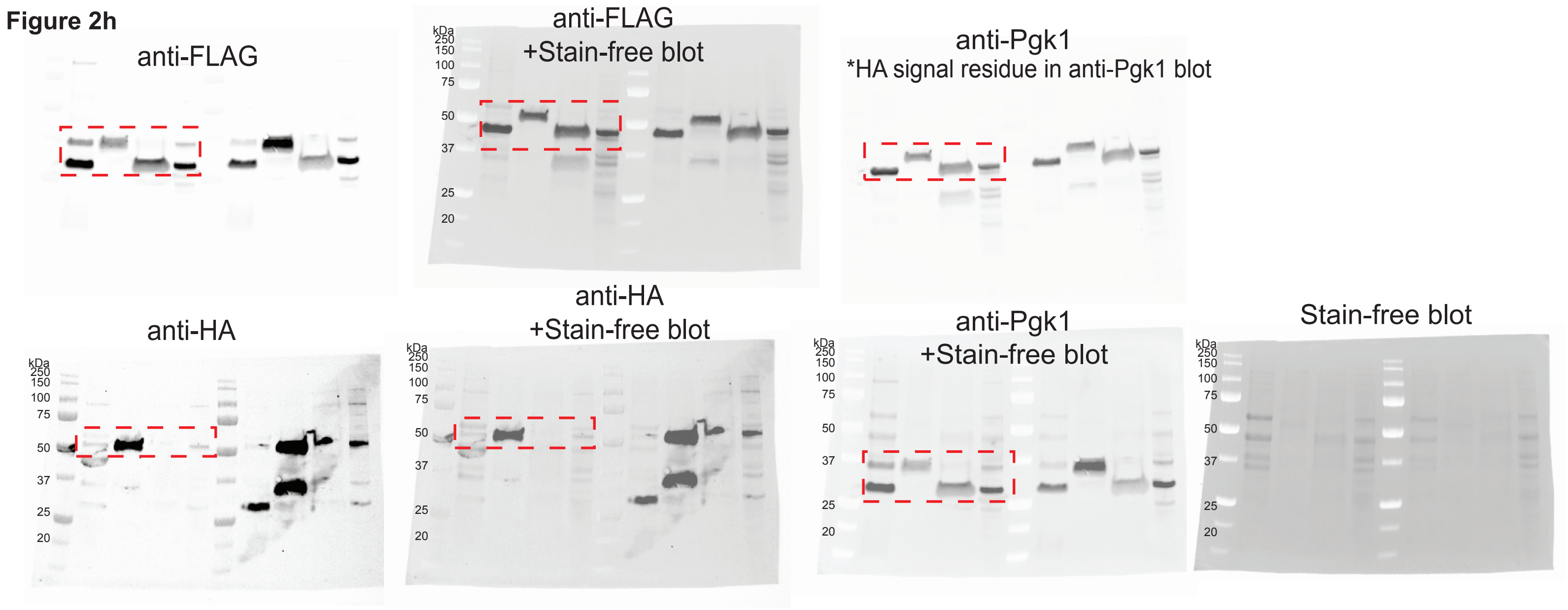

Figure 2i

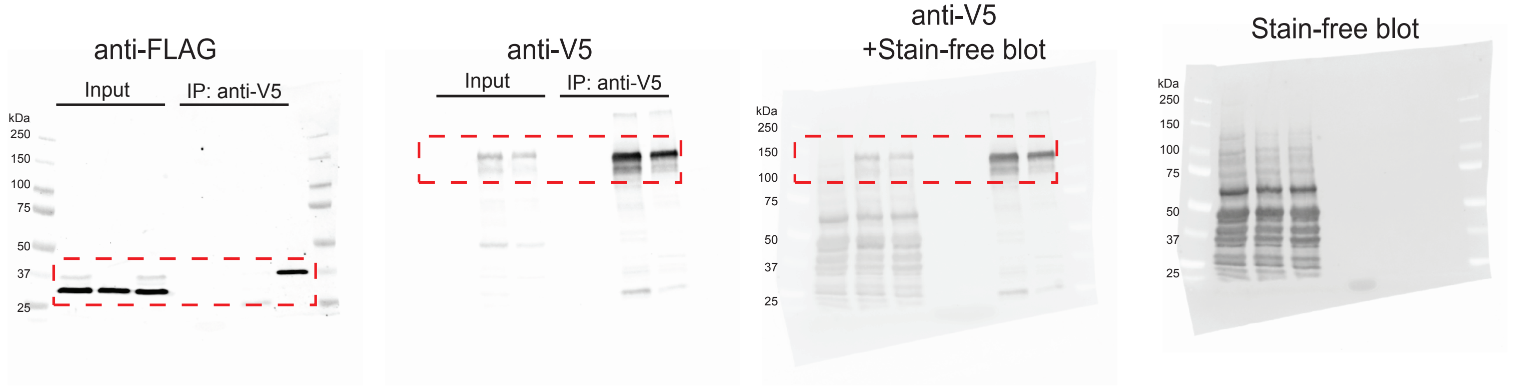

Figure 3a

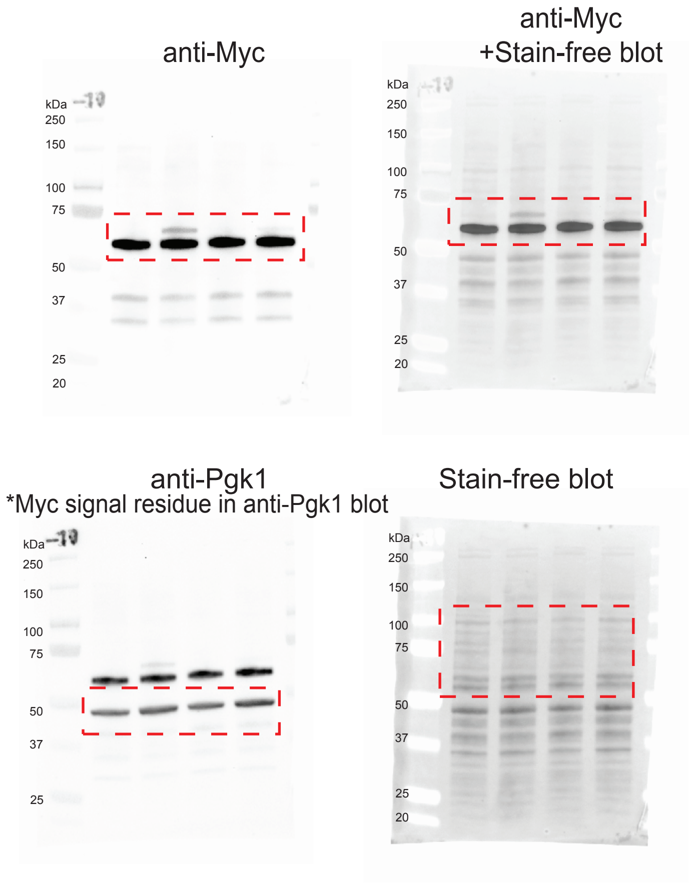

Figure 3e

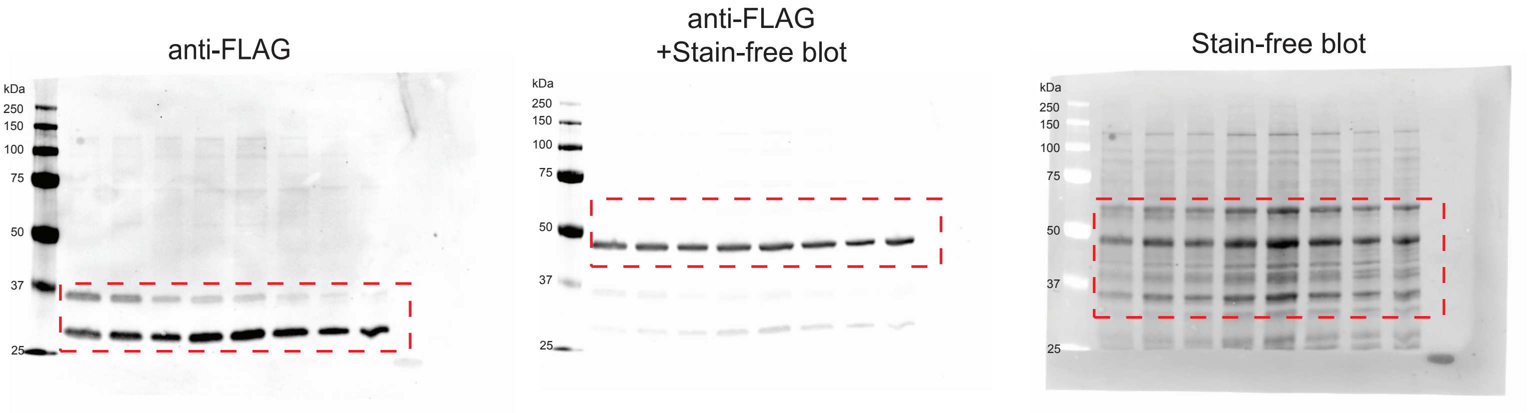

Figure 4c

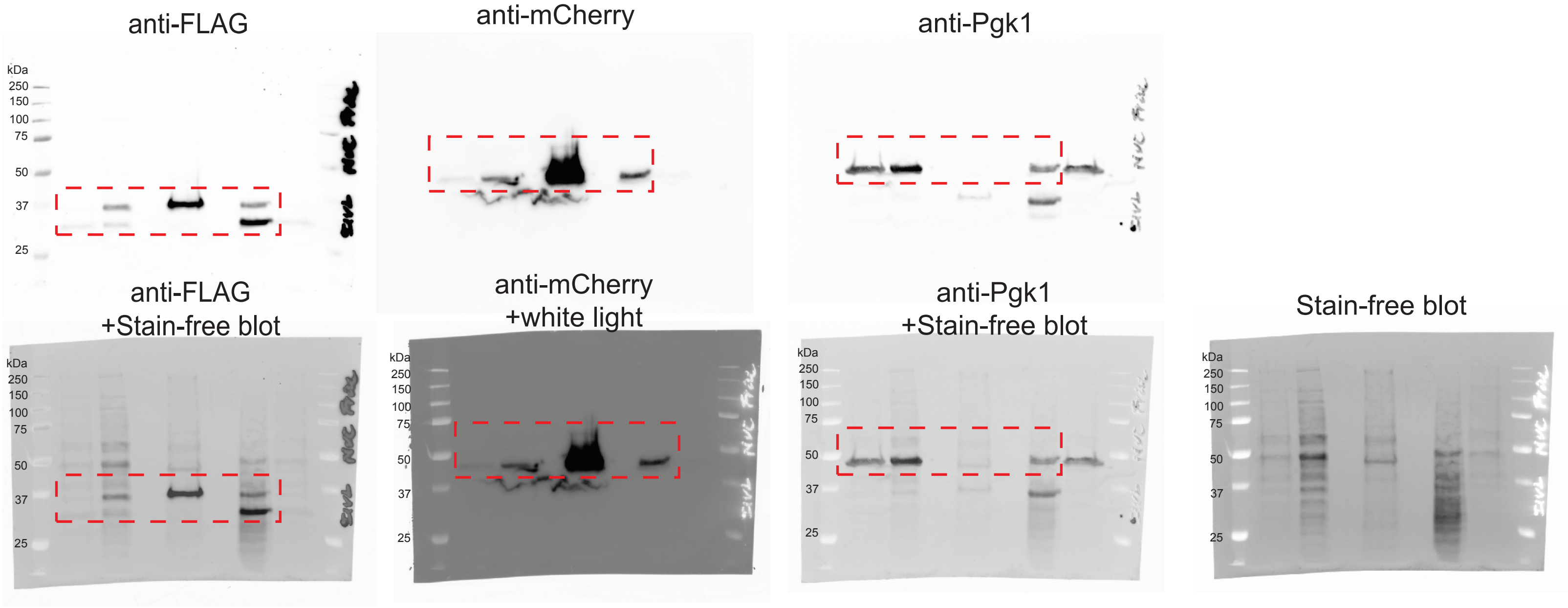

Figure 4d

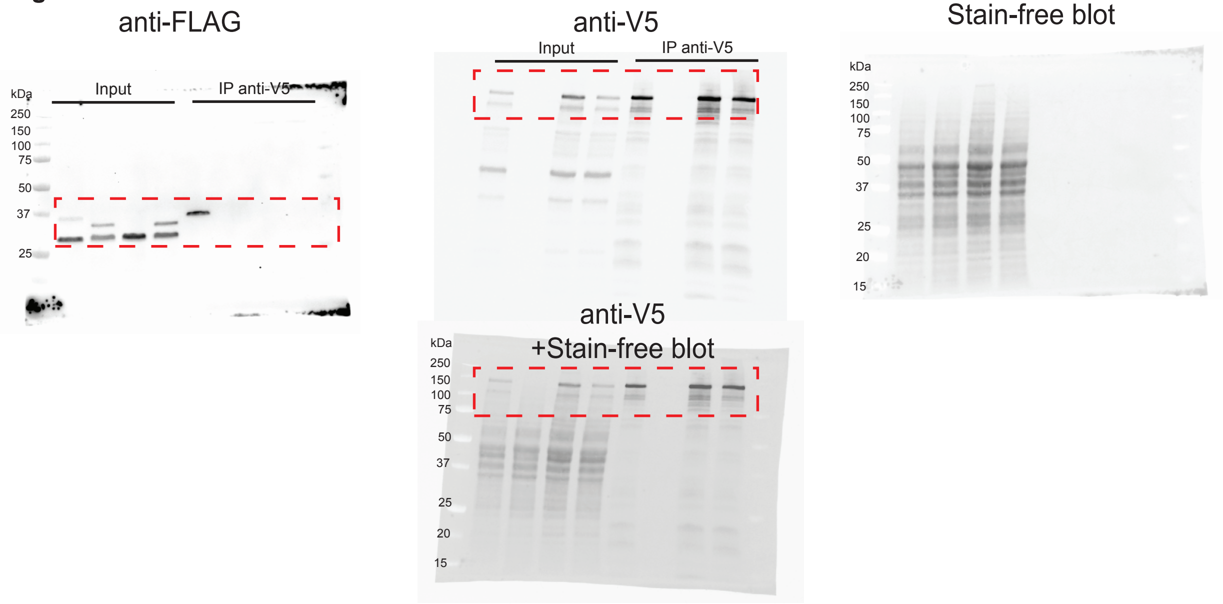

Figure 5e

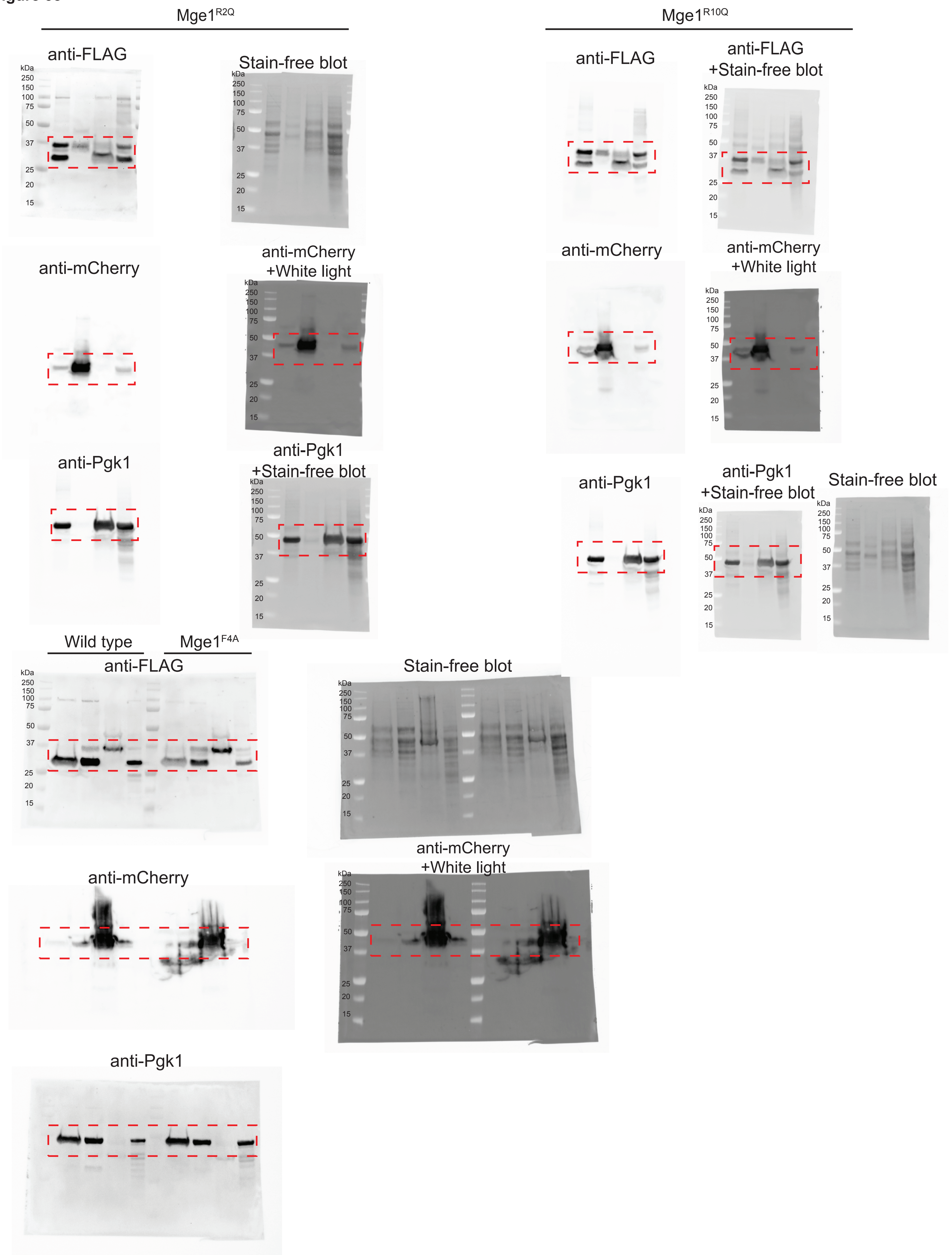
Mge1R2Q
anti-FLAG
Stain-free blot
anti-mCherry
anti-mCherry
+White light
anti-Pgk1
anti-Pgk1
+Stain-free blot
Mge1R10Q
anti-FLAG
anti-FLAG
+Stain-free blot
anti-mCherry
anti-mCherry
+White light
anti-Pgk1
anti-Pgk1
+Stain-free blot
Stain-free blot
Wild type
Mge1F4A
anti-FLAG
Stain-free blot
anti-mCherry
anti-mCherry
+White light
anti-Pgk1
kDa
250
150
100
75
50
37
25
20
15

Extended Figure 1h

anti-HA

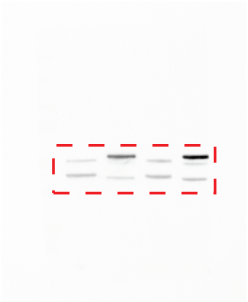

anti-HA
+Stain-free blot

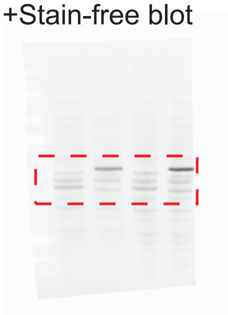

Stain-free blot

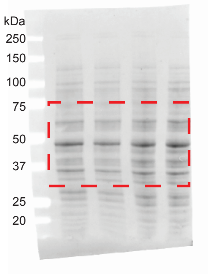

Extended Figure 3a

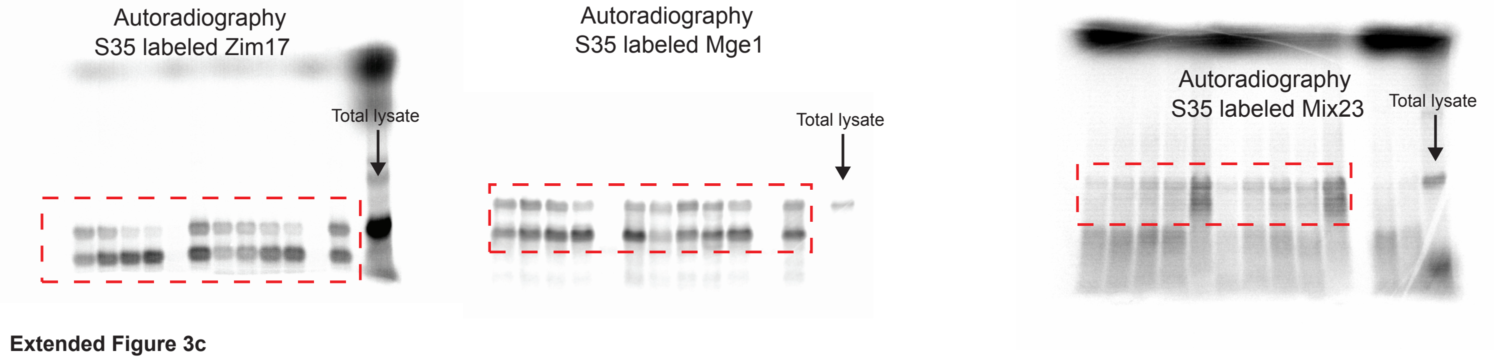

Extended Figure 3c

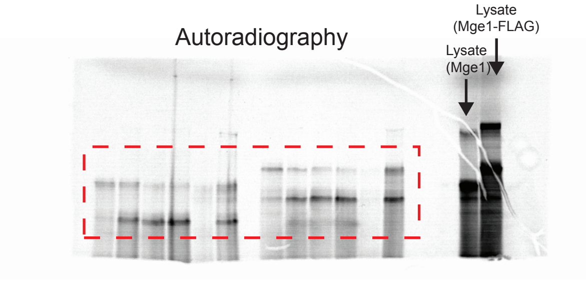

Extended Figure 3e

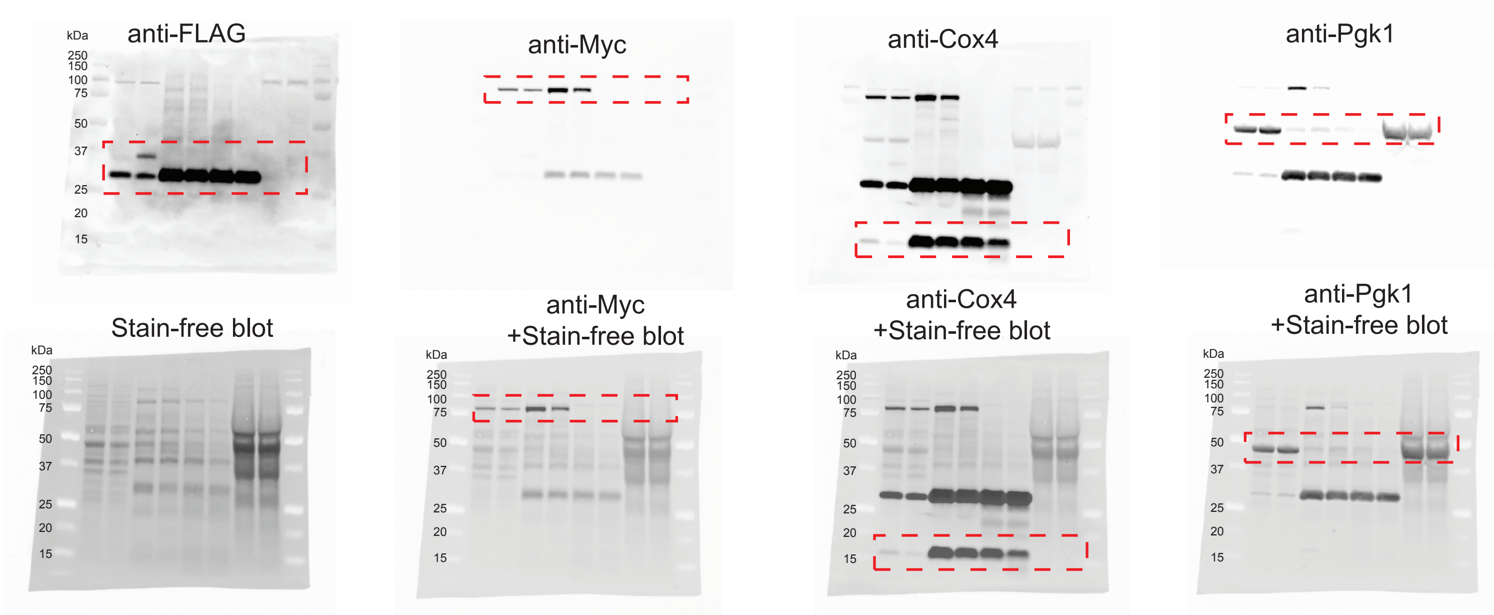

Extended Figure 3f

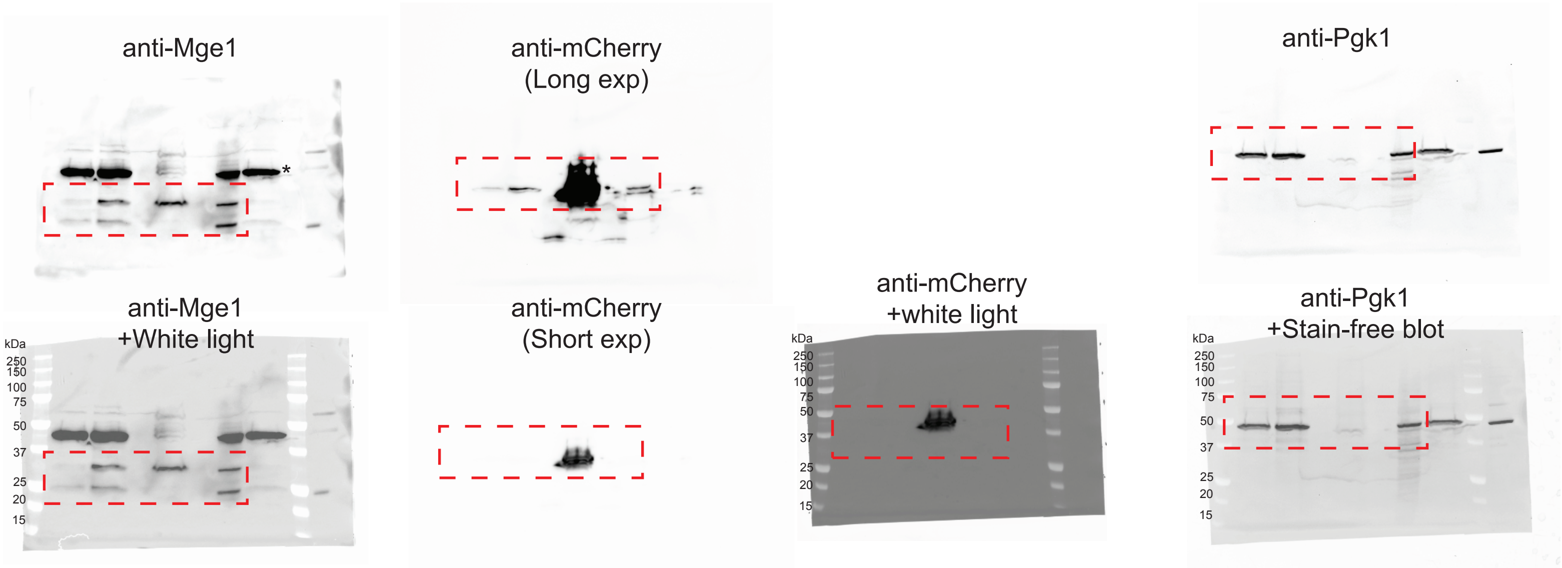

*Unspecific band from Mge1 antisera

Extended figure 4h

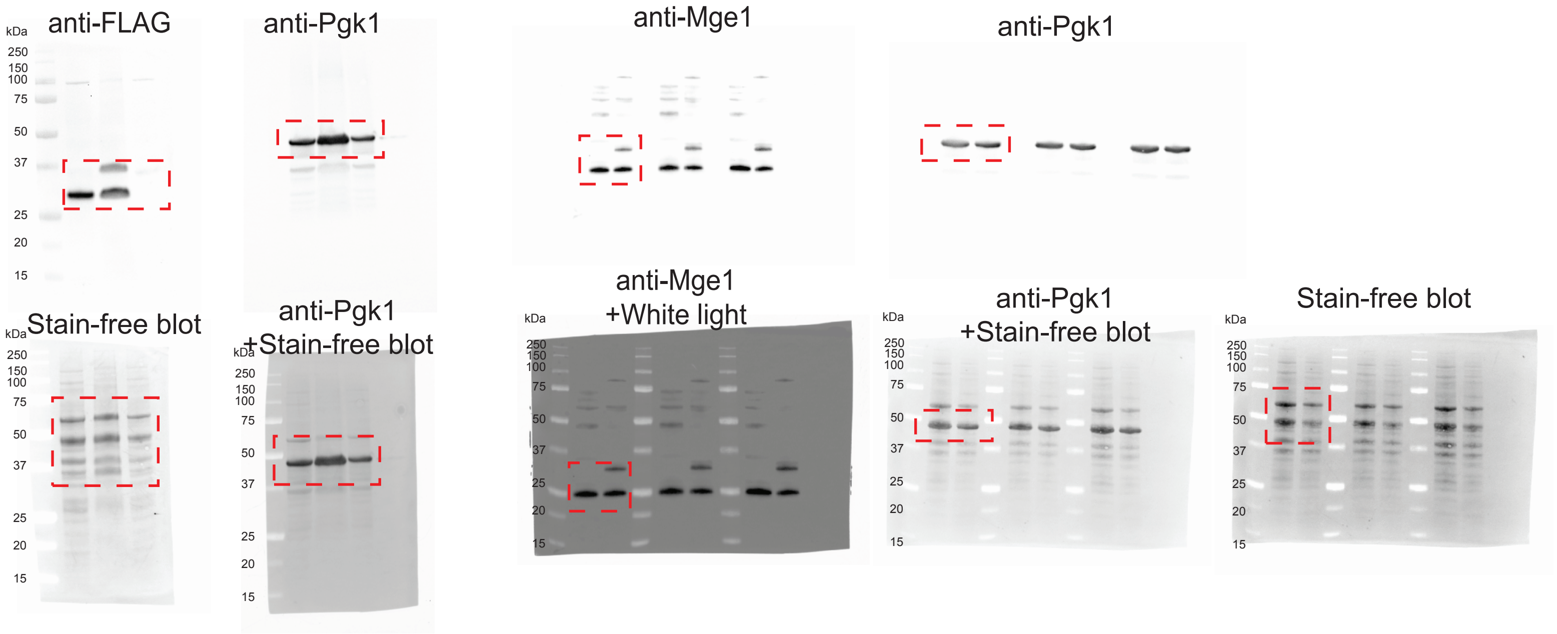

Extended figure 4i

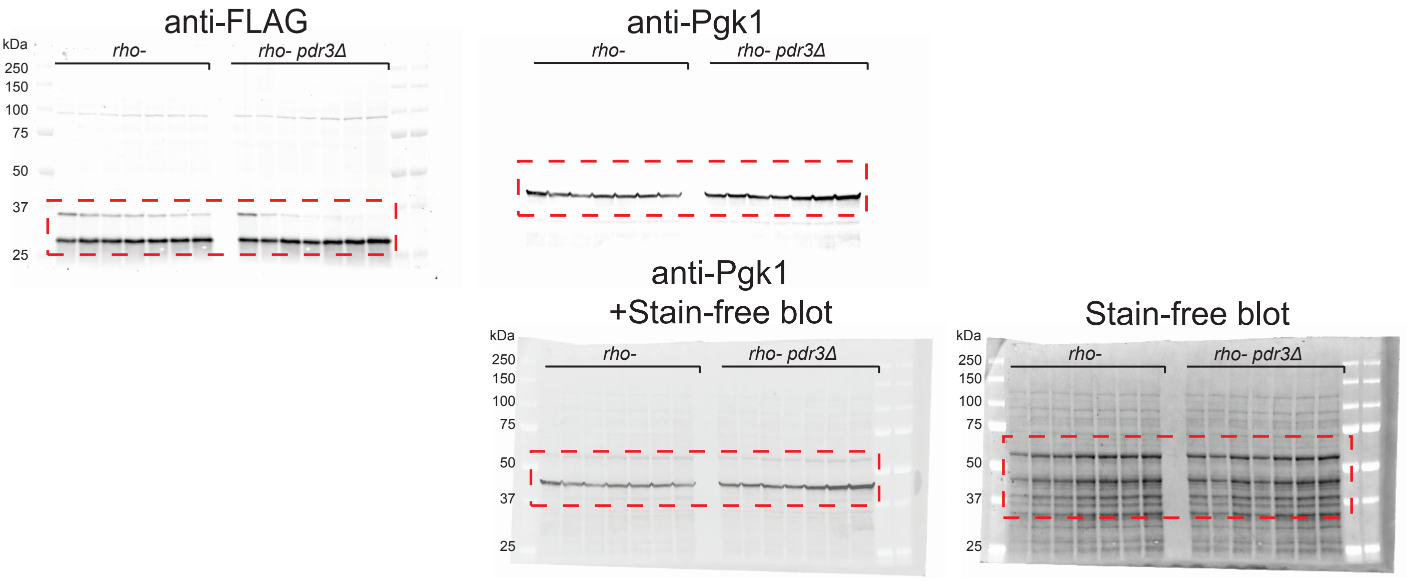

Extended figure 4j

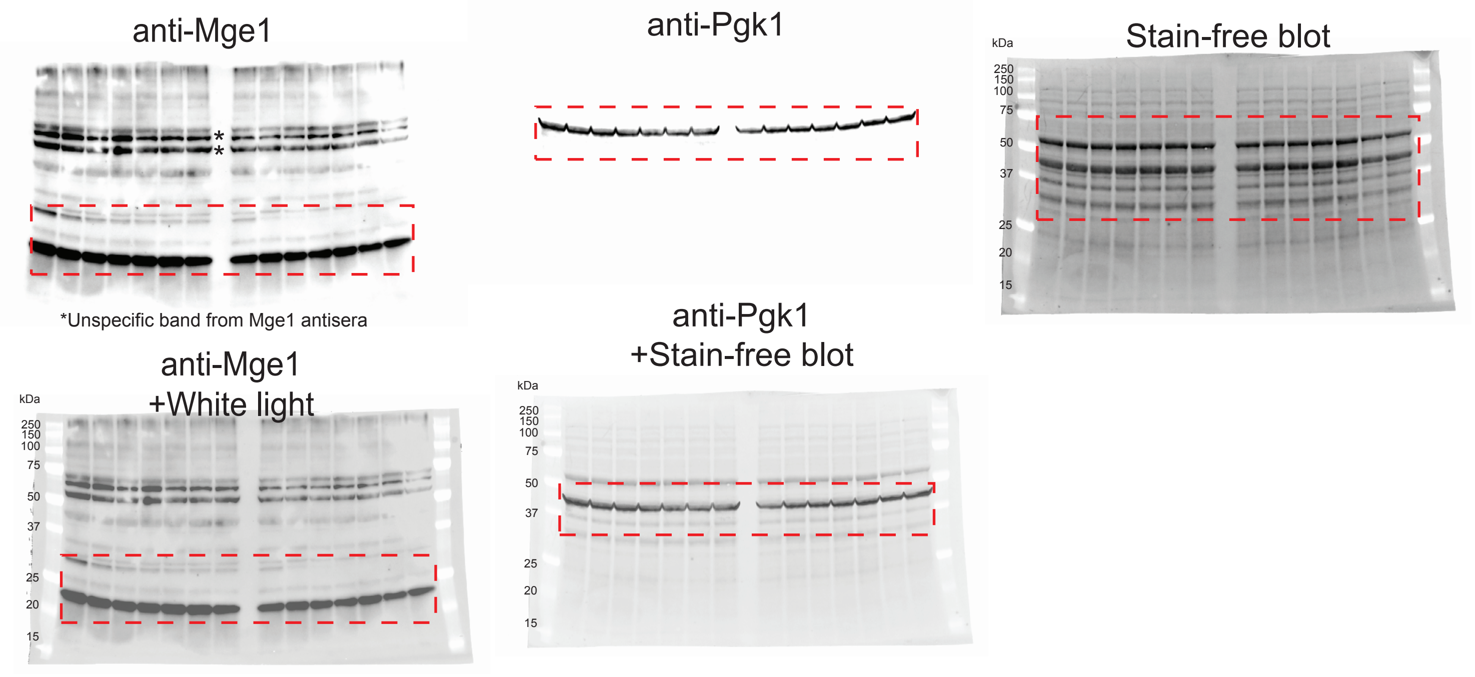

Extended figure 4k

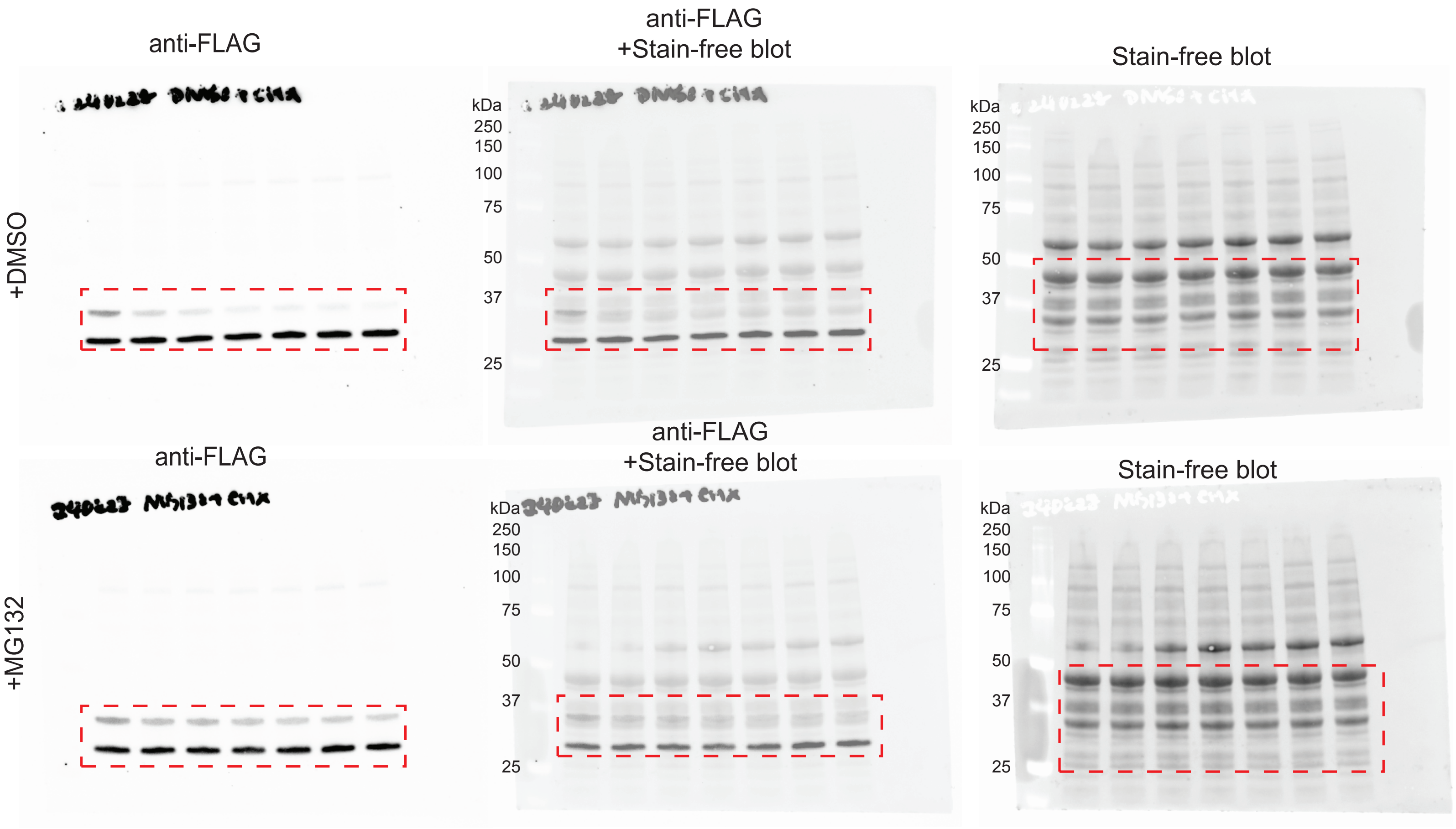

Extended figure 4b

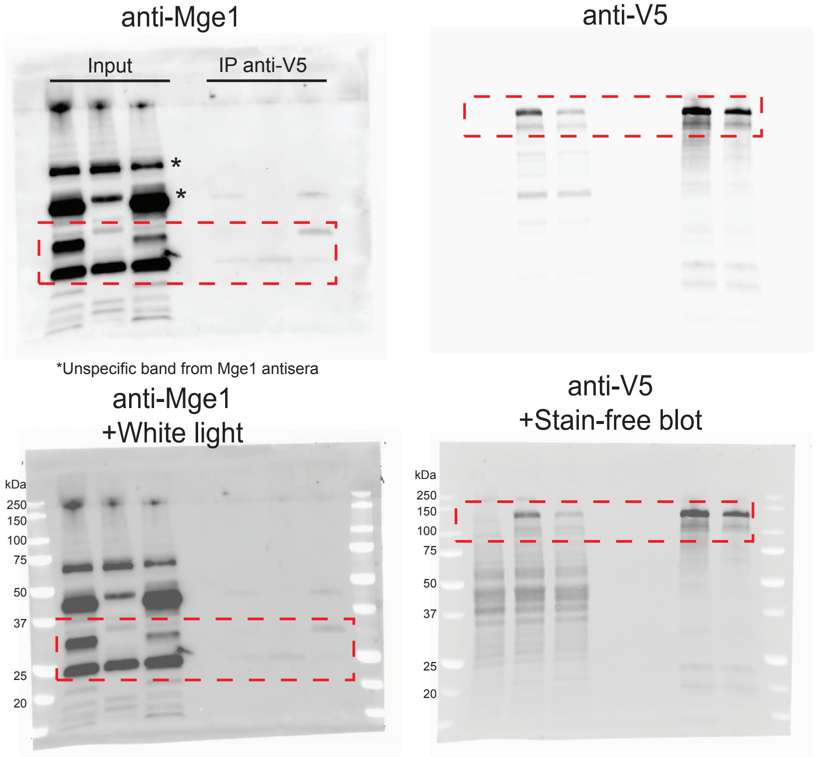

Extended figure 4c

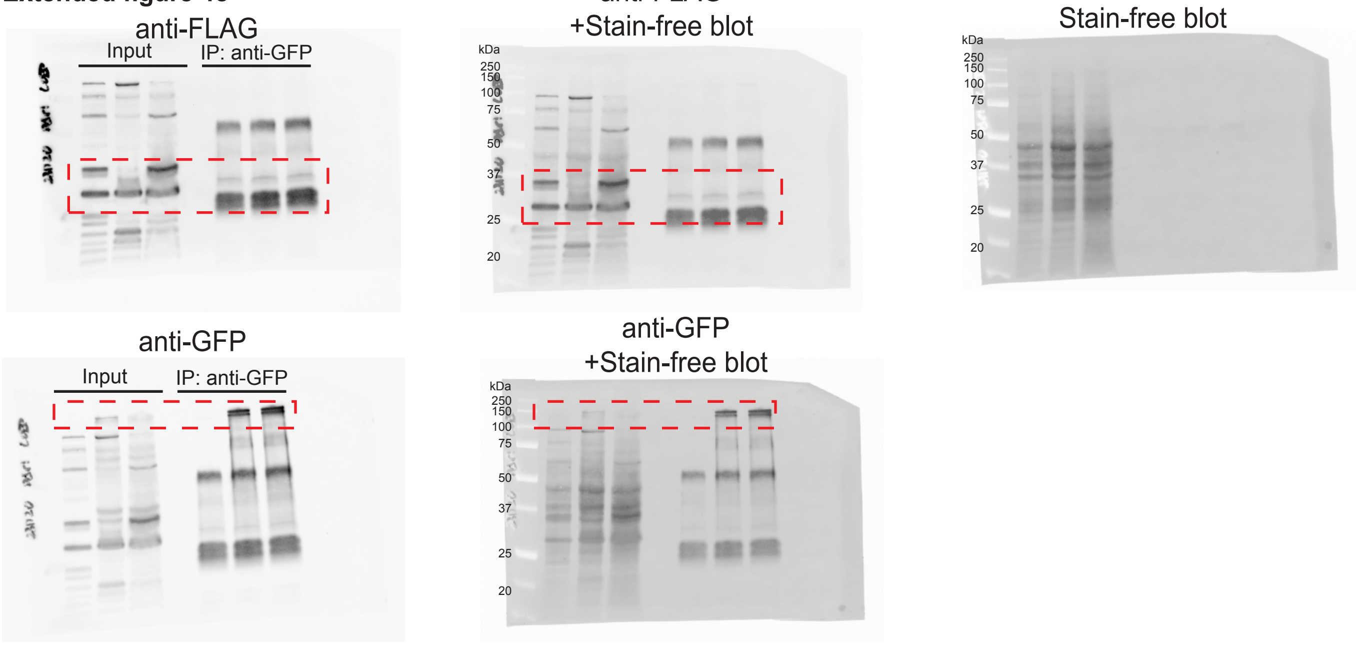

Extended figure 4g

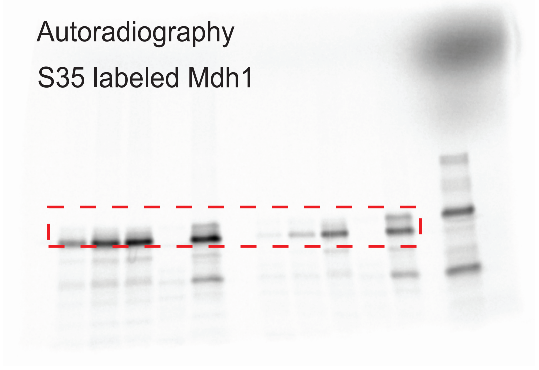

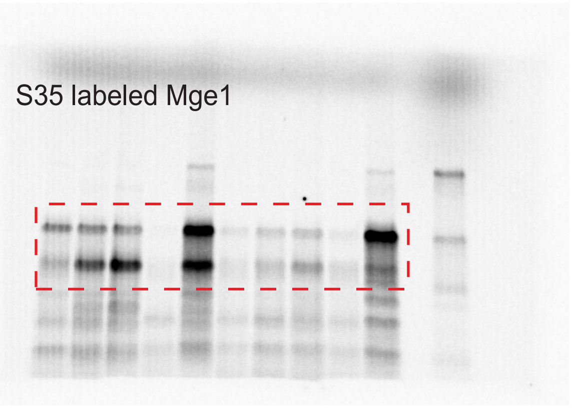

Extended Figure 5c

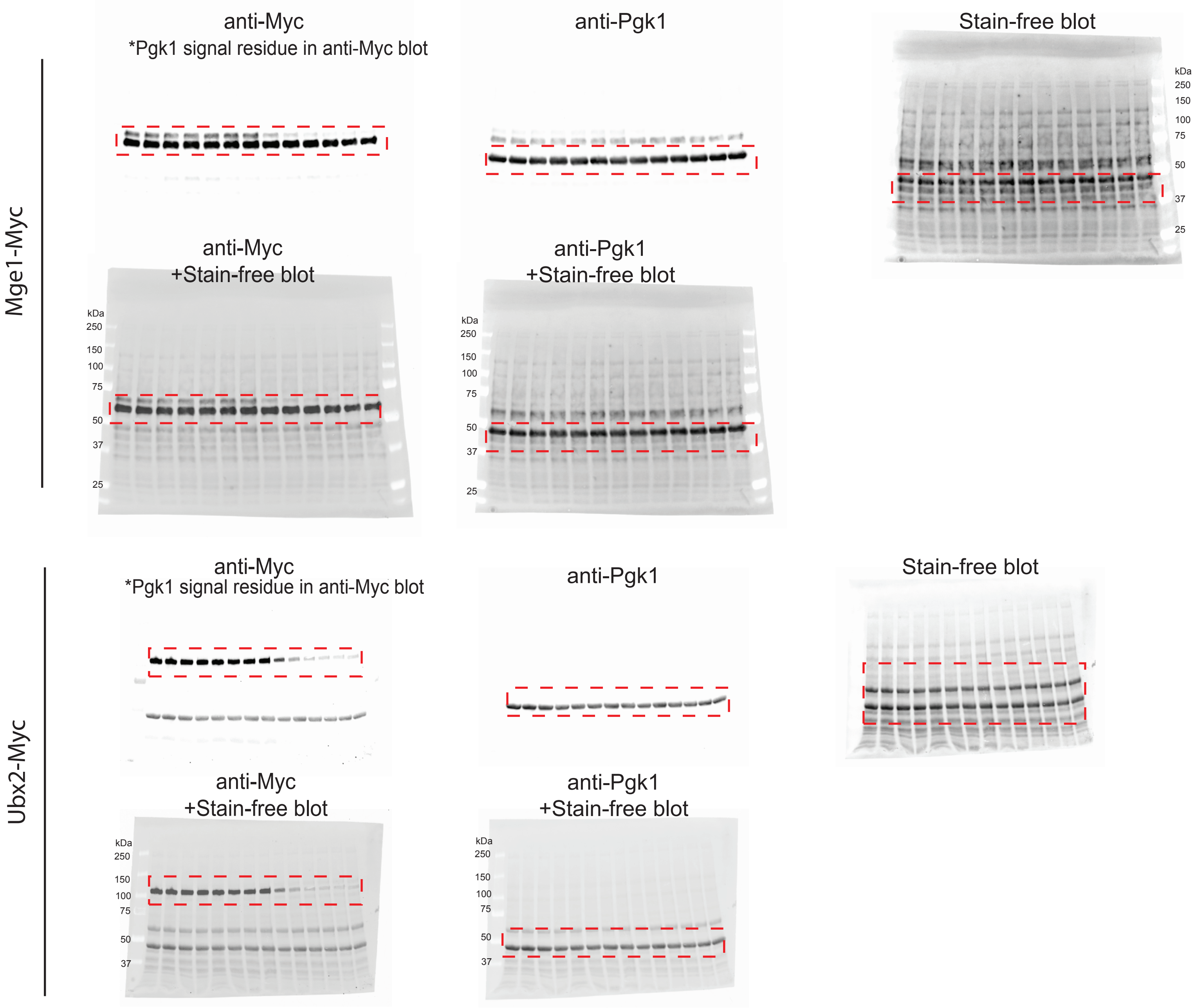

Extended Figure 5e

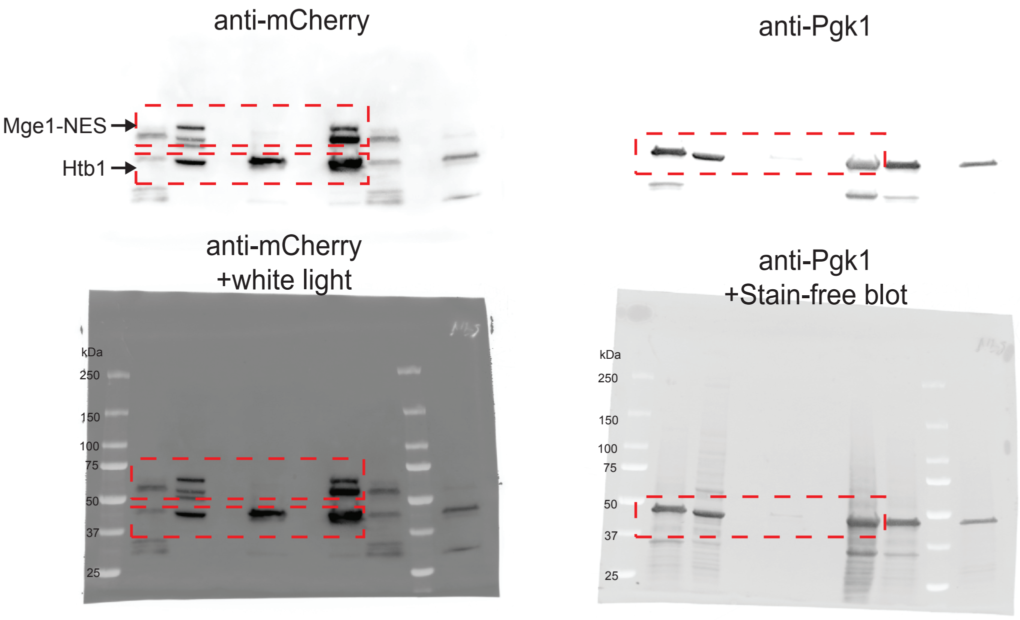

Extended Figure 6a

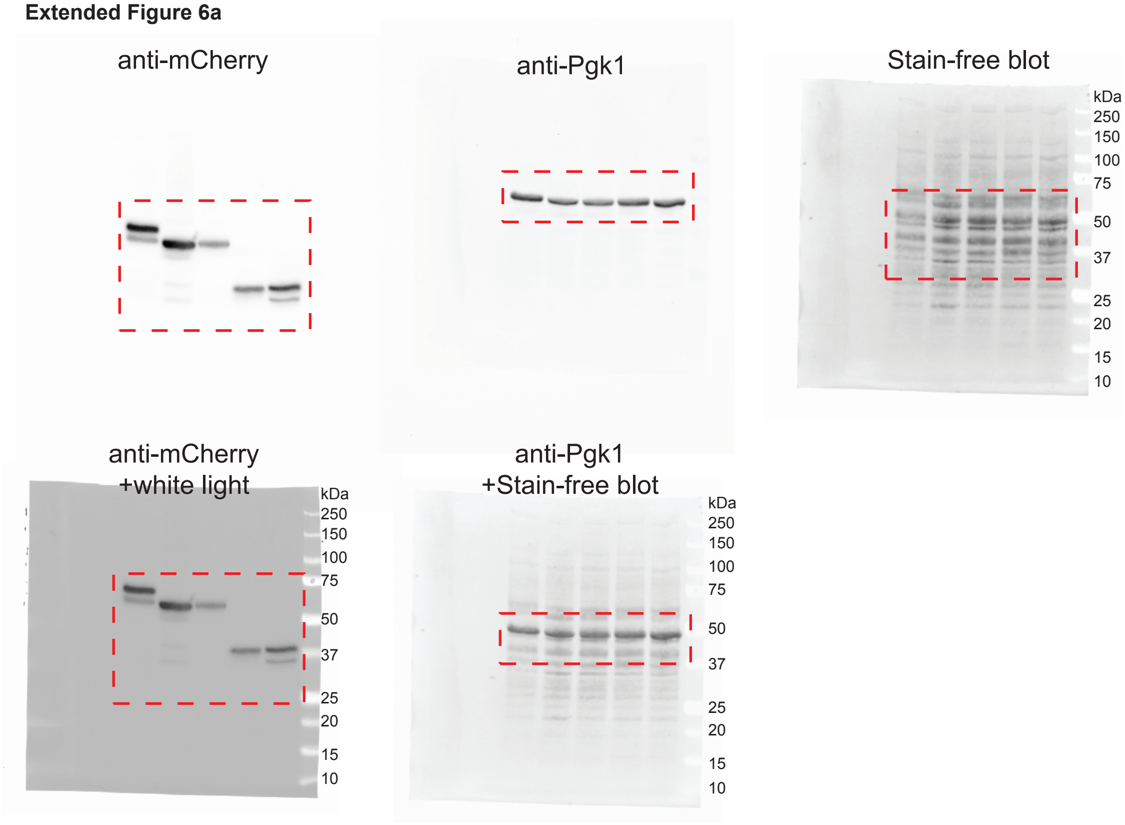

Extended Figure 6e

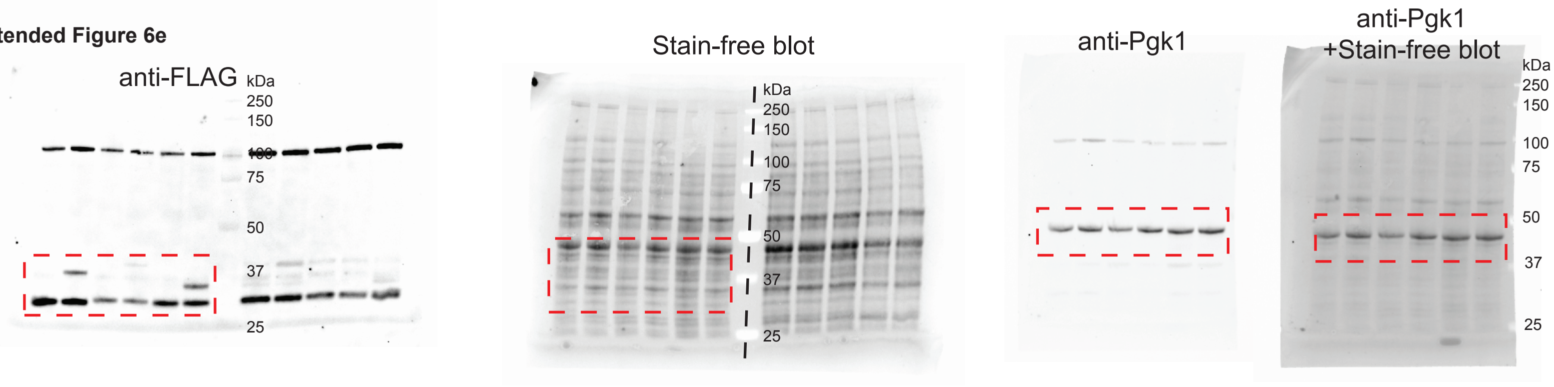

The blot was cut along the dashed line before blotting for Pgk1

Extended Figure 6g

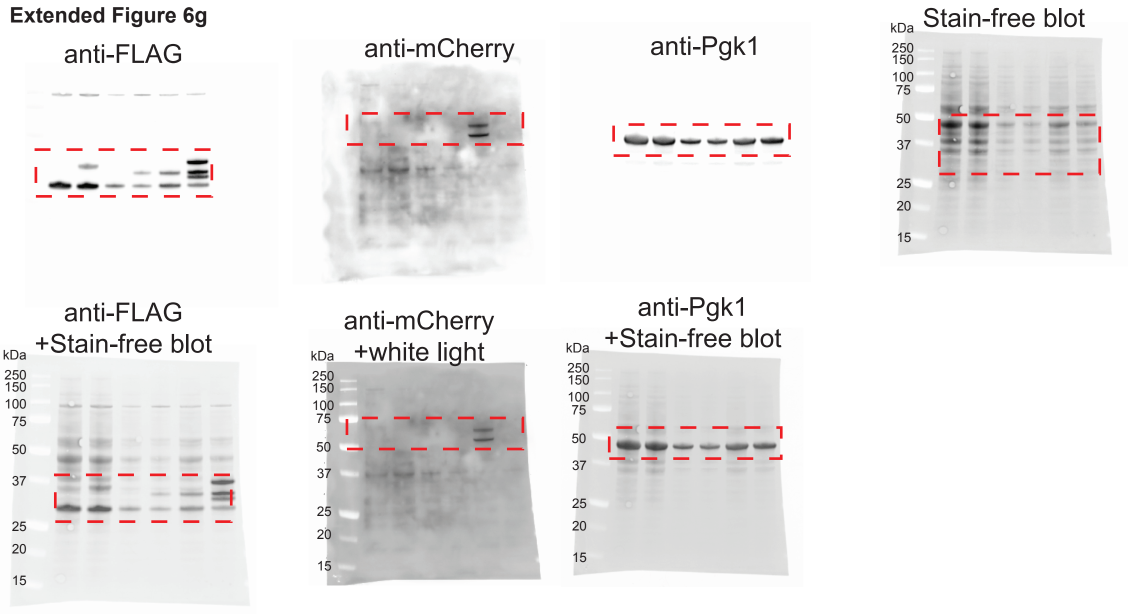

Extended Figure 6h

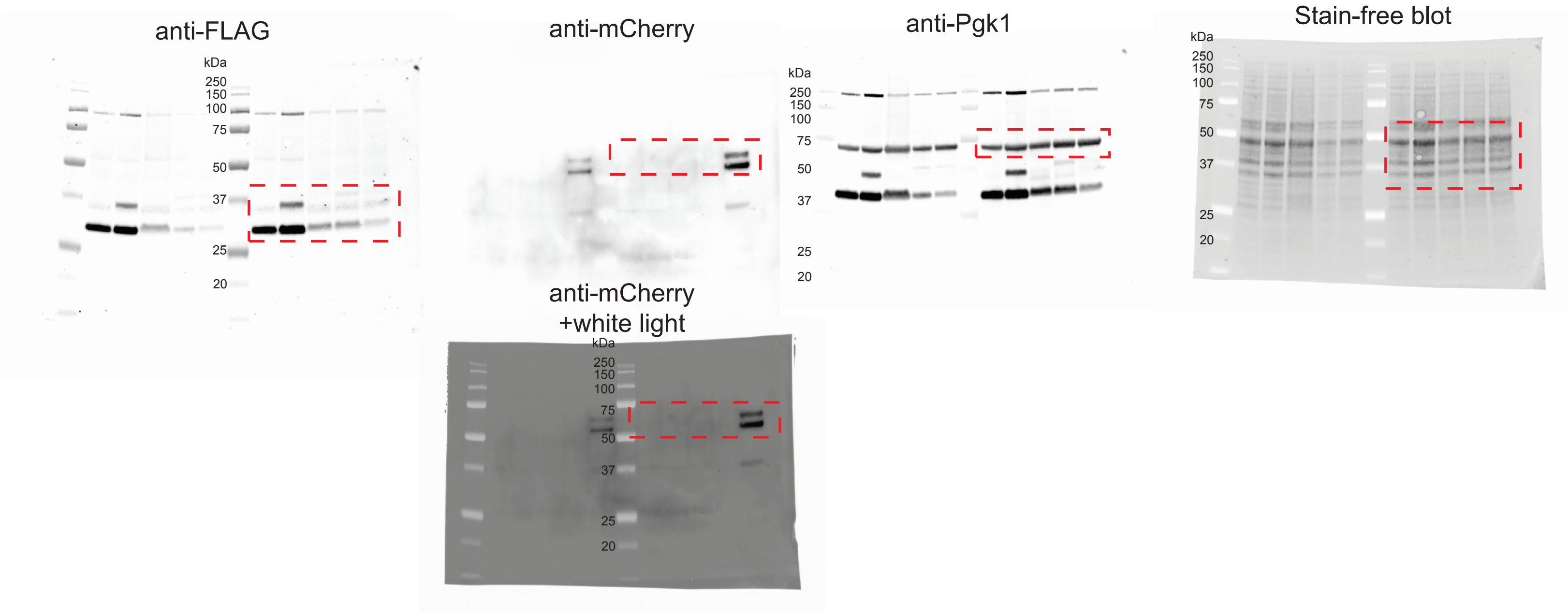

Extended Figure 6j

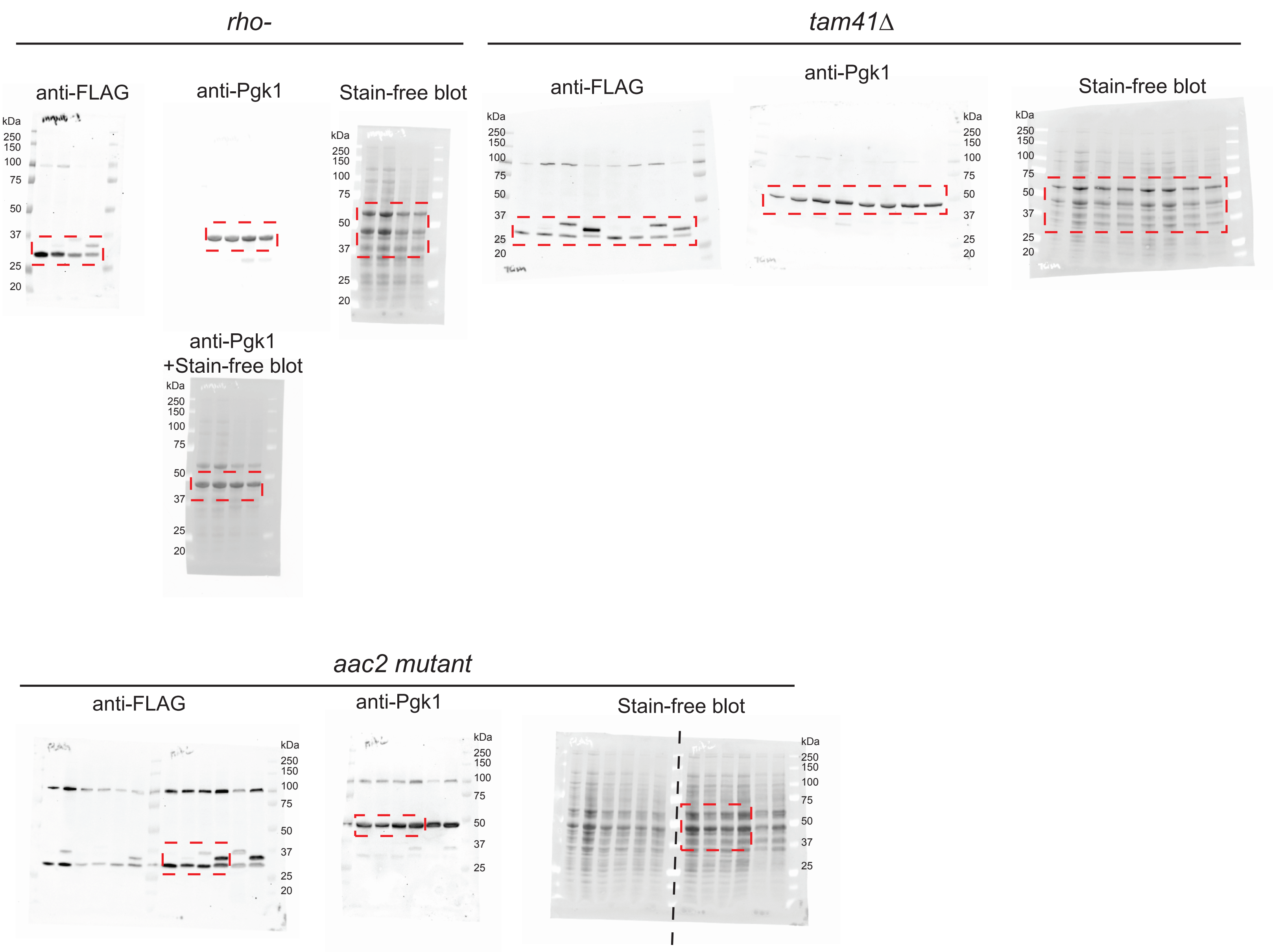

*The blot was cut along the dashed line before blotting for Pgk1

Extended Figure 7b

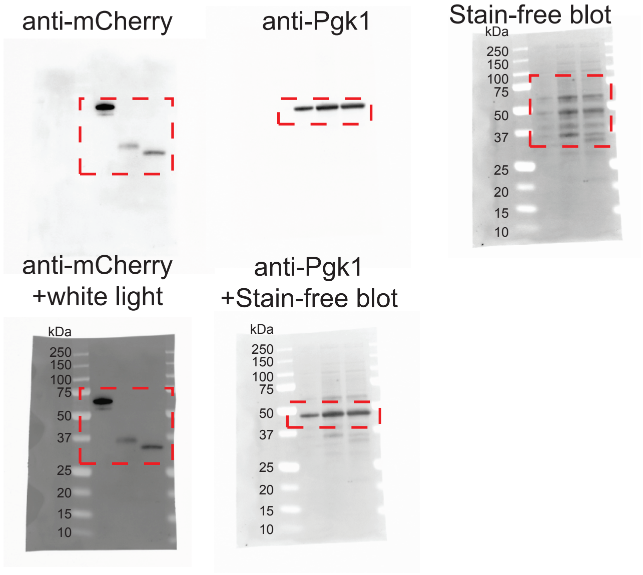

Extended Figure 7e

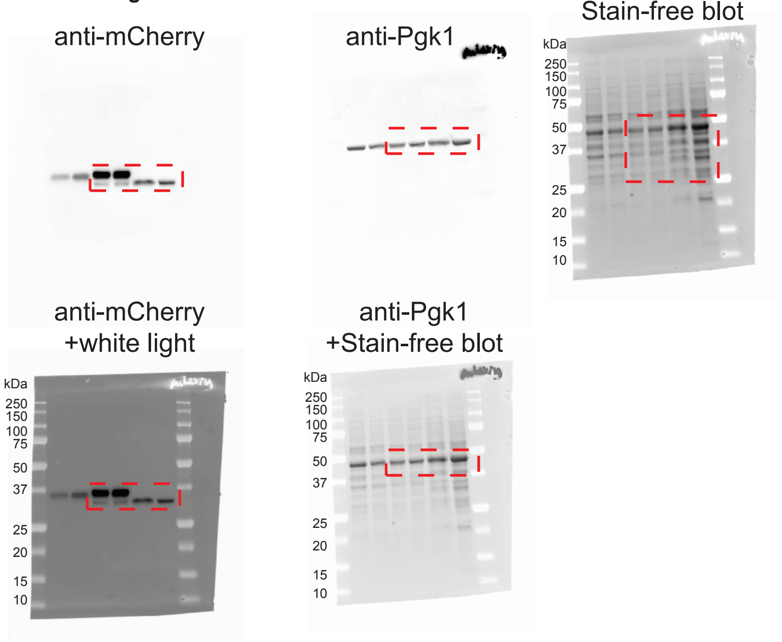

Extended Figure 7c

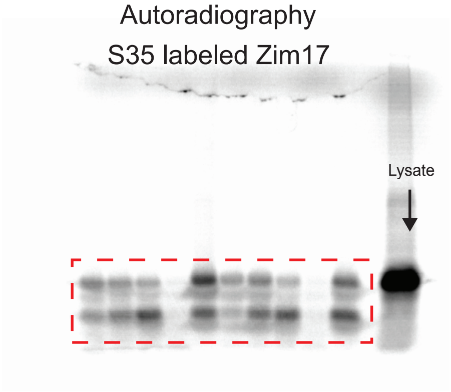

Extended Figure 7f

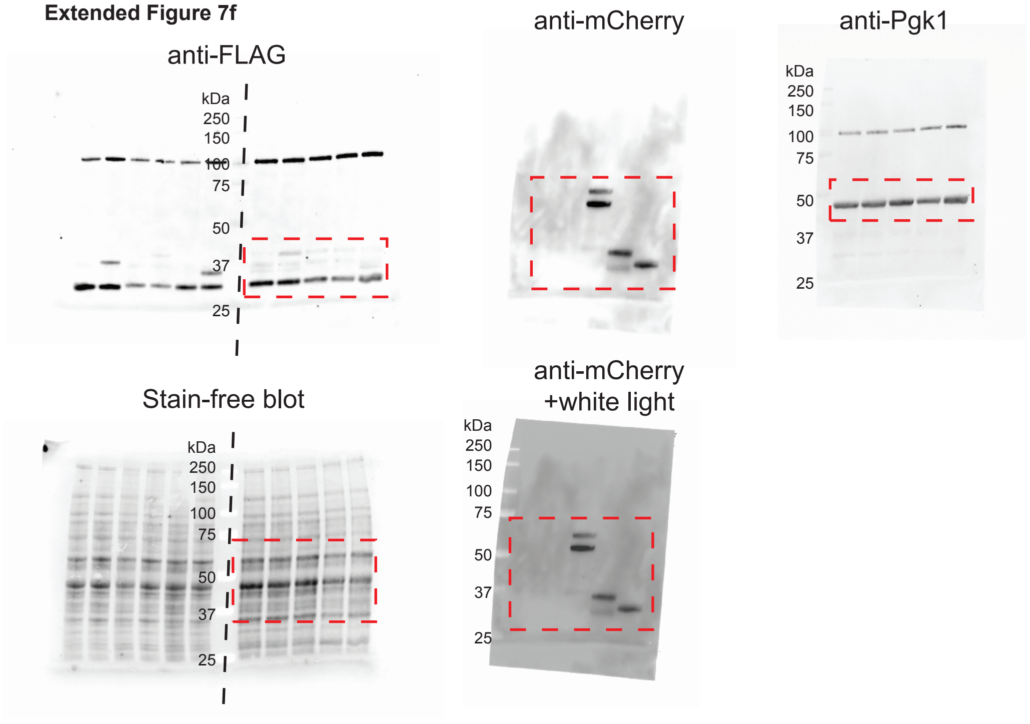

The blot was cut along the dashed line before blotting for mCherry

Extended Figure 7j

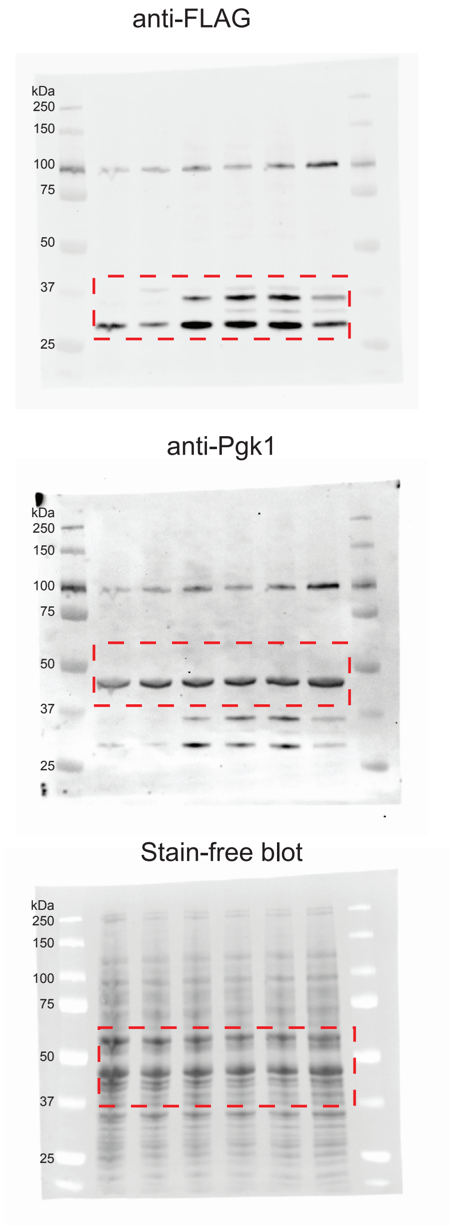

Supplementary Fig 1b

*unspecific PCR product

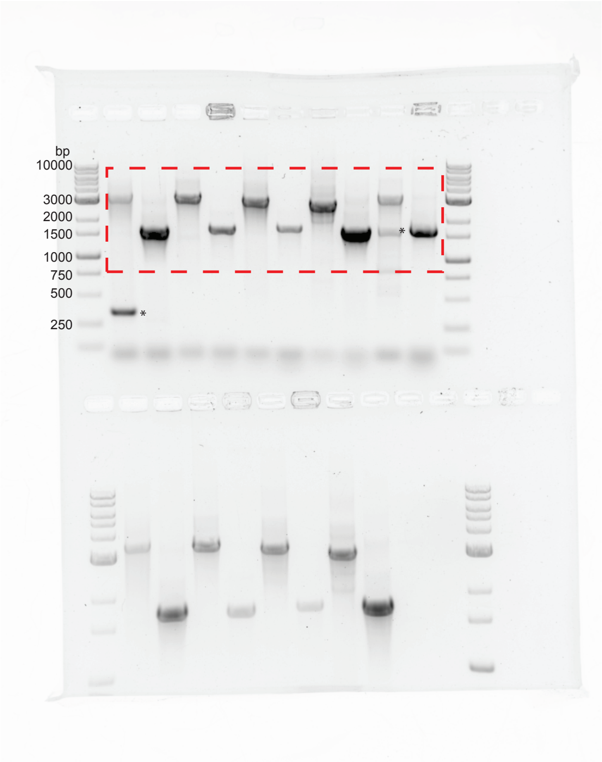

Supplement: Supplementary Figure 2 [file EMS212253-supplement-Supplementary_Figure_2.pdf]
